# Supplementary material for: Head-to-head containment performance testing of four commercially available closed-system transfer devices using the 2016 Draft NIOSH test protocol and 2.5% w/v 2-phenoxyethanol as challenge agent
Source: PLoS One. 2026 Nov 12;21(11):e0337729. doi: 10.1371/journal.pone.0337729 (PMC13759118; doi:10.1371/journal.pone.0337729)
Supplement: Supplementary file 1 [file pone.0337729.s002.pdf]

Reviewer's Comments

OVERALL FIRST IMPRESSIONS

Tone & Audience Fit:
The tone is appropriately academic and aligns well with the intended audience—healthcare professionals, pharmacists, occupational safety experts, and researchers working in oncology and hazardous drug handling. The language is formal but not unnecessarily complex, which is suitable for a journal like *PLOS ONE*.

Opening Hook:
The abstract begins effectively by stating the objective clearly: assessing the containment performance of four CSTDs using the 2016 Draft NIOSH protocol and 2-POE as a surrogate. However, the introduction could benefit from a more engaging opening sentence or paragraph that emphasizes the real-world stakes of inadequate CSTD performance (e.g., occupational exposure risks).

ABSTRACT REVIEW

Strengths:

- Clear statement of objectives.
- Concise description of methodology.
- Summary of key findings is well articulated.
- Conclusions are directly tied to the study's purpose.

Weaknesses:

- Missing context on why 2.5% w/v 2-phenoxyethanol was selected: This should be briefly explained in the abstract, especially since it's central to the test method.
- Statistical significance vs. practical relevance: While statistical differences were noted between some CSTD types, the abstract does not clarify what those differences mean in practice (e.g., magnitude of exposure risk).
- Lack of mention of sample size or replicates: Readers may want to know how many trials were conducted per device.

Suggested Improvements:

"This study evaluated the containment performance of four commercially available closed-system transfer devices (CSTDs) using the 2016 Draft NIOSH universal test protocol and 2.5% w/v 2-phenoxyethanol (2-POE), a semi-volatile surrogate selected for its representative behaviour compared to hazardous drugs."

Add:
"Results are based on [X] replicate tests per device, with statistical comparisons made using [method]."

INTRODUCTION

Flow & Logical Development:

The introduction builds up to the research question logically, starting with background on hazardous drugs, moving into CSTD classifications, then discussing prior protocols leading to the 2016 NIOSH draft.

Strengths:

- Solid foundation on the importance of CSTDs.
- Good explanation of the limitations of earlier testing methods.
- Clear rationale for adopting the 2016 NIOSH protocol.

Areas for Improvement:

- Repetition of content: Some paragraphs repeat similar points about IPA volatility and surrogate selection across different sections. Consolidate these ideas for better flow.
- Need for clearer transition: Between the discussion of surrogate selection and the actual methodology used in this study. A sentence such as "Given these considerations, we selected 2-POE at a concentration of 2.5% w/v…" would help bridge the gap.

Suggested Edit:

"While IPA has been widely used historically, its high volatility makes it unsuitable for accurately simulating the vapor release characteristics of most hazardous drugs. In response, the 2016 Draft NIOSH protocol proposed the use of semi-volatile surrogates, including 2-phenoxyethanol (2-POE). Based on these guidelines, we selected 2.5% w/v 2-POE as the challenge agent for our comparative assessment of four CSTDs."

METHODS SECTION

Overall Assessment:

The methods section is generally thorough and follows the 2016 Draft NIOSH protocol closely. However, several areas require clarification or expansion to meet the standards expected in a peer-reviewed publication.

Key Issues:

1. Surrogate Selection Justification

- What led to the choice of 2.5% w/v? Is this concentration within the range recommended by NIOSH or derived empirically?
- Was there any pre-study compatibility testing with the tested CSTD materials? As noted in the literature (e.g., Simplivia white paper), some solvents can degrade tubing or seals.

2. Device Testing Protocol

- Were all manipulations performed strictly according to IFUs? If so, specify whether deviations were recorded or monitored.
- Were negative controls included? For example, baseline chamber contamination levels before each test.

3. Analytical Methods

- Clarify ATD-GC-MS parameters: What were the temperature settings, desorption times, column type, etc.? Without this detail, reproducibility is compromised.
- Calibration curves: Were they generated fresh for each batch of samples? How was linearity confirmed?

4. Sample Size and Replication

- How many replicates were run per device? This is essential for interpreting statistical confidence in results.
- Were outliers excluded? If yes, under what criteria?

5. Statistical Methods

- Which statistical tests were used? T-tests, ANOVA, non-parametric equivalents?
- Was multiple comparison correction applied? Given the number of pairwise comparisons, this should be addressed.
- Define what constitutes a statistically significant difference (e.g., $p < 0.05$).

Suggested Additions:

"All testing procedures were performed strictly in accordance with manufacturer instructions for use (IFU) to ensure validity. Deviations, if any, were documented and reviewed for potential impact on containment integrity."

"To assess repeatability, five replicate tests were conducted for each CSTD model. Outliers were identified using the Grubbs' test and removed only when statistically justified."

"Chromatographic separation was achieved using a DB-5MS capillary column (30 m × 0.25 mm × 0.25 µm; Agilent Technologies) with helium as the carrier gas at a constant flow rate of 1.2 mL/min. Calibration was verified daily using freshly prepared standards."

RESULTS SECTION

Strengths:

- Clear presentation of data.
- Tables summarize findings effectively.
- Statistical outcomes are reported where applicable.

Areas for Clarification:

- Figure/Table Correspondence: Ensure every figure and table is explicitly referenced in the text and that their captions are self-explanatory.
- Units and precision: Make sure units (e.g., ppbv) are consistent throughout and that decimal places reflect measurement precision.

Example:

"As shown in Table 2, ChemoLock™ barrier demonstrated significantly lower vapour leakage than Tevadaptor™ air-cleaning (p = 0.008)."

DISCUSSION

Strengths:

- Well-grounded in previous literature.
- Interpretation of results aligns with known properties of CSTD technologies.
- Acknowledges limitations of the current methodology.

Weaknesses:

- Overemphasis on protocol validation: While important, the discussion should center more on the implications of the observed differences among the CSTDs.
- Limited interpretation of practical significance: Beyond statistical significance, what do the differences mean for healthcare worker exposure? Are the measured concentrations below regulatory thresholds?

Suggested Revisions:

"The observed differences in vapour containment among the tested CSTDs suggest that not all systems offer equivalent protection against occupational exposure. These findings support the need for standardised, performance-based evaluation methods such as the 2016 Draft NIOSH protocol."

"Further research is needed to correlate laboratory-measured vapour leakage with real-world exposure scenarios to determine whether these differences translate into clinically meaningful reductions in risk."

CONCLUSION

Strengths:

- Directly addresses the research objective.
- Succinct and clear.

Suggestion:

Consider rephrasing the conclusion to emphasise the broader implication:

"These findings underscore the importance of adopting standardised, semi-volatile surrogates and quantitative testing methodologies to ensure reliable and comparable assessment of CSTD performance in protecting healthcare workers."

FIGURES AND TABLES

General Observations:

- All tables appear clear and relevant.
- Figures (if present) must be checked for resolution and labelling consistency.

To Do:

- Confirm that all figures are labelled with axis titles, units, and legends.
- Ensure that statistical annotations (e.g., asterisks for p-values) are consistent and explained in the caption.

LIMITATIONS SECTION

A dedicated limitations section is missing. Include one after the discussion to address:

- Potential influence of surrogate concentration on device behaviour.
- Absence of real-world validation.
- Limited scope of CSTD models tested.
- Possible variability in operator technique despite adherence to IFUs.

FINAL COMMENTS

While the manuscript presents scientifically relevant data and follows a rigorous testing protocol, it currently falls short of PLOS ONE's standards for clarity, methodological transparency, and ethical disclosure. The following critical issues must be addressed before publication is warranted:

Major issues preventing recommendation for publication

1. Incomplete or Inconsistent Methods Section

- Lack of reproducibility details: Key aspects such as GC-MS parameters (e.g., column type, temperature ramping), surrogate preparation, and calibration procedures are either missing or underdeveloped.

- Insufficient statistical reporting: It is unclear which tests were used to compare device performance (e.g., ANOVA, t-test), whether multiple comparisons corrections were applied, or how outliers were handled.

Recommendation: Expand the Methods section with full technical specifications and statistical methodology. Ensure that another lab could replicate your work using only this description.

2. Missing or Inadequate Limitations Section

- The study lacks a dedicated limitations section, which is essential for contextualising findings and acknowledging constraints.
- No discussion is provided on:
  - Whether the selected surrogate concentration (2.5% w/v 2-POE) affects CSTD integrity or behavior differently than actual hazardous drugs.
  - Whether operator variability was controlled or monitored.
  - The generalizability of findings beyond the four tested CSTDs.

Recommendation: Add a standalone Limitations section addressing these points and others related to internal and external validity.

3. Inconsistent Data Reporting and Figure/Table Integration

- Some figures and tables are referenced incorrectly or not at all in the main text.
- Units and formatting (e.g., ppbv, µL) are inconsistent across sections.
- There is no clear statement confirming whether raw data will be made publicly available, despite PLOS ONE's strict open-data policy.

Recommendation: Conduct a comprehensive review of all figures and tables to ensure:

- Every one is explicitly cited in the text.
- Captions are self-explanatory.
- Units are consistent and SI-compliant.
- A Data Availability Statement is clearly included, indicating where data can be accessed.

4. Ethical and Funding Disclosures Require Clarification

- Although the authors state that "no competing interests exist," they also disclose funding from ICU Medical, which provided materials and devices used in the study. This creates a potential conflict of interest that needs explicit acknowledgment.

- There is ambiguity around the role of the funder: While the authors claim the funders had no influence, this should be stated more transparently.

Recommendation: Revise the Competing Interests and Funding Disclosure sections to fully align with PLOS ONE’s requirements. Consider including the following language:

“The funders had no role in study design, data collection and analysis, decision to publish, or preparation of the manuscript.”

5. Abstract and Conclusions Need Tightening

- The abstract lacks key information about sample size, statistical methods, and practical implications.
- The conclusion emphasizes protocol validation over real-world relevance.

Recommendation:

- Clarify what the differences between CSTD types mean for occupational safety.
- Emphasize how this study fills a gap in published evidence.
- Reframe conclusions to guide healthcare institutions in interpreting containment performance.

Strengths that support eventual acceptance

Despite these issues, the manuscript has several strengths that make it worth revising for eventual publication:

- Scientific merit: It addresses an important gap by comparing air-cleaning and barrier CSTDs using a semi-volatile surrogate under a standardised protocol.
- Rigorous methodology: Adherence to the 2016 Draft NIOSH protocol is commendable.
- Potential impact: Findings may inform purchasing decisions and regulatory guidance if interpreted carefully
