# Supplementary material for: Head-to-head containment performance testing of four commercially available closed-system transfer devices using the 2016 Draft NIOSH test protocol and 2.5% w/v 2-phenoxyethanol as challenge agent
Source: PLoS One. 2026 Nov 12;21(11):e0337729. doi: 10.1371/journal.pone.0337729 (PMC13759118; doi:10.1371/journal.pone.0337729)
Supplement: Supplementary file 3 [file pone.0337729.s004.docx]

31^st^ October, 2025

Editor-in-Chief

Email: [plosone@plos.org](mailto:plosone@plos.org)

*Plos One*

Dear Editor,

On behalf of myself and my co-authors, I respectfully request your consideration of the accompanying revised manuscript, entitled “Head-to-head containment performance testing of four commercially available closed-system transfer devices using the 2016 draft NIOSH test protocol and 2.5% w/v 2-phenoxyethanol as challenge agent,” for publication as an original research article in *Plos One*.

The authors previously published an article in *Plos One* based on methodology developed by the same authors in collaboration with the UK Health and Safety Executive (Ian Pengelly, HSE) and submitted to CDC NIOSH in 2015 which later became adopted by CDC NIOSH as the 2016 draft NIOSH protocol for assessment of containtment performance of CSTDs.

Wilkinson *et al* published original research in 2018 on the development, validation and application of the 2016 draft NIOSH test protocol for assessing the containment performance of four CSTDs using the NIOSH surrogate 2.5% w/v 2-phenoxyethanol (2-POE) in MilliQ water as diluent [7].

The manuscript submitted in January 2025 has previously been reviewed by two independent reviewers at *Plos One* and was accepted for publication following review. However, due to a technical issue with uploading a file to support copyright use of an image (figure 3) the manuscript was then withdrawn by *Plos One* and the authors were requested to make a re-submission. The attached revised manuscript is the re-submitted manuscript which has now been subjected to further review by two reviewers.

**Please see below the responses to the latest reviewers’ questions:**

1. Is the manuscript technically sound, and do the data support the conclusions?

The manuscript must describe a technically sound piece of scientific research with data that supports the conclusions. Experiments must have been conducted rigorously, with appropriate controls, replication, and sample sizes. The conclusions must be drawn appropriately based on the data presented.

Reviewer #1: Yes

Reviewer #2: Partly

Author Response: Review #2 Please provide further clarification to support your statement that the research is only partly robust.

The authors methodology is already published in Plos One (2018) and was adopted by the CDC NIOSH in 2016 forming what is now known as the 2016 draft NIOSH protocol for CSTDs. This methodology allows for quantitative measurement of a released challenge agent by employing time weighted average (TWA) collection of the surrogate 2-phenoxyethanol (2-POE) on to a sorbent tube from a closed chamber of fixed volume 125 Litres. The methodology is therefore superior to the predecessor 2015 draft NIOSH protocol proposed by the NIOSH which measured Isopropyl alocohol (IPA) surrogate in a flow-through setup which results in mixing and dilution of any released IPA material from the CSTDs tested in the chamber [5]. The authors methodology adopted by the CDC NIOSH in 2016 is a protocol that was based and therefore aligns with the UK Health and Safety Executive method mdhs104 (see reference 8 below) for assessment of volatile organic compounds using sorbent tubes, solvent desorption or thermal desorption and gas chromatography [8]. Air sampling methods form the basis of many standard analytical methods used by the United Kingdom (UK) Health and Safety Executive (HSE) and the US Occupational Safety and Health Administration (OSHA) for monitoring levels of volatile and semi-volatile materials in the environment and therefore has precedence in the literature and within regulated environments.

I hope that the above references to the methodology satisfy the reviewer and editor that this is scientifically robust and has passed many different levels of internal and external review by experts in the field. The authors developed the protocol in conjunction with the UK leading experts in occupational health and safety at the UK Health and Safety Executive (Ian Pengelly, HSE UK).

2. Has the statistical analysis been performed appropriately and rigorously?

Reviewer #1: Yes

Reviewer #2: Yes

Author Response: There is no response needed to this question.

 3. Have the authors made all data underlying the findings in their manuscript fully available?

The [PLOS Data policy](http://track.editorialmanager.com/CL0/http:%2F%2Fwww.plosone.org%2Fstatic%2Fpolicies.action%23sharing/1/010f0197ac6b2902-4a37b94e-3639-4bd7-854e-276225653697-000000/RQP1KyuZctRxKMo7OftttNwpZRSyM5UvKBP1YKL5RXs=217) requires authors to make all data underlying the findings described in their manuscript fully available without restriction, with rare exception (please refer to the Data Availability Statement in the manuscript PDF file). The data should be provided as part of the manuscript or its supporting information, or deposited to a public repository. For example, in addition to summary statistics, the data points behind means, medians and variance measures should be available. If there are restrictions on publicly sharing data—e.g. participant privacy or use of data from a third party—those must be specified.

Reviewer #1: Yes

Reviewer #2: No

Response: Review #2 This is not correct as all data has been made available. The authors used commercially available proprietary CDS software from Thermo Scientific (Chromeleon v7.3) to collect the RAW instrument data which could be made available if requested but is not readable outside of the Chromeleon v7.3 environment. Anyone that is skilled in the art would know that RAW instrument data can not be manipulated outside of the CDS software.

4. Is the manuscript presented in an intelligible fashion and written in standard English?

PLOS ONE does not copyedit accepted manuscripts, so the language in submitted articles must be clear, correct, and unambiguous. Any typographical or grammatical errors should be corrected at revision, so please note any specific errors here.

Reviewer #1: Yes

Reviewer #2: Yes

Author Response: There is no response needed to this question.

**Author Summary:**

The author has satisfied all of the above reviewer requirements and provided a robust rebuttal to the Reviewer #2 question that the manuscript described a technically sound piece of scientific research based on published scientific methodology adopted by internationally recognized government agencies involved in occupational health and safety research – the CDC NIOSH (USA) and the UK HSE.

More detailed rebuttals (where relevant) are provided below

5. Review Comments to the Author

Please use the space provided to explain your answers to the questions above. You may also include additional comments for the author, including concerns about dual publication, research ethics, or publication ethics. (Please upload your review as an attachment if it exceeds 20,000 characters)

Reviewer #1: The study is thoroughly done
Design is appropriate
Statistics suitable
Conclusions are in accordance with data and acceptable
No ethical problems or plagiarism seen
It is a long paper and could be shortened for faster readability

Response: Review #1 The comment regarding the length of the article is a reasonable comment. The authors have made efforts to reduce the length of the article without removing key arguments and important information. The submitted manuscript is of extreme importance in the context of NIOSH testing of CSTD medical devices in providing the only published protocol that can generate quantitative release data and results on CSTD containment. There is still considerable misinformation and misunderstanding within the scientific community on what constitutes an appropriate surrogate to employ in the testing of CSTDs. Many researchers are still employing the 2015 draft NIOSH test protocol despite the CDC NIOSH stating that 70% IPA is not an appropriate surrogate to use. The authors have themselves provided comments to the CDC NIOSH regarding incompatibility between medical devices and 70% IPA (registered on the federal docket at CDC NIOSH). Given the importance of using an appropriate surrogate such as 2-POE to generate meaningful containment data, the authors felt the length of the manuscript was suggested by using it as a forum for wider discussion around surrogate selection and the criteria used to select an appropriate surrogate. Currently there is a paucity of data from researchers applying this latest 2016 draft protocol with most new published works being based on the 2015 draft NIOSH protocol which does not provide robust quantitative data. As such the authors believe that the manuscript length is justified as it makes a significant and valuable contribution to the discussion around appropriate surrogate selection for use in NIOSH testing of CSTDs and advances the understanding of science in this area.

Reviewer #2: OVERALL FIRST IMPRESSIONS
Tone & Audience Fit:
The tone is appropriately academic and aligns well with the intended audience—healthcare professionals, pharmacists, occupational safety experts, and researchers working in oncology and hazardous drug handling. The language is formal but not unnecessarily complex, which is suitable for a journal like PLOS ONE.
Opening Hook:
The abstract begins effectively by stating the objective clearly: assessing the containment performance of four CSTDs using the 2016 Draft NIOSH protocol and 2-POE as a surrogate. However, the introduction could benefit from a more engaging opening sentence or paragraph that emphasizes the real-world stakes of inadequate CSTD performance (e.g., occupational exposure risks).

Response: Review #2 The comment regarding the hook is a valid one, however there are many tens of scientific papers published on CSTDs and the occupational health risks around handing of hazardous drugs that the authors took a conscious decision that this area has already been covered extensively by other authors. Given the length of the manuscript is already considerable (see comment above review #1) the authors did not want to add additional words without significant justification. The authors have included words and language and made the case for the need for CSTD technology to prevent accidental exposure of health workers to hazardous drugs and readers familiar with this area will be aware of those cited articles. The manuscript is already lengthy and so for brevity reasons the authors would like to resist making further changes that could add to the word count but thank the reviewer for their comments.

ABSTRACT REVIEW
Strengths:
• Clear statement of objectives.
• Concise description of methodology.
• Summary of key findings is well articulated.
• Conclusions are directly tied to the study’s purpose.
Weaknesses:
• Missing context on why 2.5% w/v 2-phenoxyethanol was selected: This should be briefly explained in the abstract, especially since it's central to the test method.

Response: Review #2 In connection with the context on why 2.5% w/v 2POE was selected for use in the testing. This is covered elsewhere in the previous publication by the same authors in Plos One (2018) and published in the 2016 draft NIOSH protocol which is also cited by the authors and is an official US published method. As such the authors believe that the reviewer’s comment is not correct. The authors also cite the basis of selection of 2-POE as coming from the UK Health and Safety Executive research and cite this pivotal work which has received little attention to date. The authors make it quite clear why the selected 2-POE as surrogate as “it more closely resembles real hazardous drugs” in terms of volatility and vapour pressure values. This is a key message, and the poor suitability of other highly volatile NIOSH surrogates such as IPA and ethanol is also clearly addressed by the authors. The authors respectfully do not accept this criticism but thank the reviewer for their comment on this important point.

For further corroboration See Page 14

“It was largely on the basis of this work that Pengelly *et al* of the UK Health and Safety Executive and Wilkinson *et al* recommended to NIOSH the inclusion of 2-POE as a candidate for NIOSH to consider in its surrogate selection in the 2016 Draft NIOSH universal protocol [28,14].“

See references 28 and 14 in main manuscript document.

• Statistical significance vs. practical relevance: While statistical differences were noted between some CSTD types, the abstract does not clarify what those differences mean in practice (e.g., magnitude of exposure risk).

Response: Review #2 The quantitative data reported shows the level of containment of each CSTD as evaluated or put more simply how “closed” the CSTD technologies are when evaluated using the 2016 draft NIOSH protocol. The authors discuss how this data allows the end user, health worker and researchers to assess the different CSTD devices and rank them in a hierarchical order based on their release values for 2-POE. The acceptance criteria is provided by the authors and each CSTD is assessed as to whether it resulted in above or below measurable levels of released 2-POE. The authors state that it is not possible to correlate releases of 2-POE with an exposure risk from actual releases of hazardous drugs but there is a relationship between CSTDs that release more 2-POE and those that would release more hazardous drug during operation. As such the CSTDs with the lowest 2-POE release values represent the safest technologies to use and it is the objective of the manuscript to provide robust scientific data on the containment of different CSTD devices and the authors believe that goal is achieved in this publication. At this present time many researchers and healthcare workers are using data from the previous 2015 draft NIOSH protocol based on the releases of 70 IPA as surrogate which is not scientifically robust or quantitative data and as such the authors believe that the manuscript submitted provides better quality data for health workers to make an informed decision as to which CSTD technology to use in their institution.

The authors has included the new sentence in the introduction “However, not all CSTDs offer the same level of operator protection and it is therefore necessary to assess the containment of each CSTD technology using a scientific robust methodology.” To satisfy the reviewer requirements and the author would like to thank the reviewer for this input and suggested amendment.

• Lack of mention of sample size or replicates: Readers may want to know how many trials were conducted per device.

Response: Review #2 This is not correct as all data has been made available and clearly shows that all tests were repeated for each CSTD technology tested I replicate where n=4 (four tests for both Test 1 and Test 2).
Suggested Improvements:
“This study evaluated the containment performance of four commercially available closed-system transfer devices (CSTDs) using the 2016 Draft NIOSH universal test protocol and 2.5% w/v 2-phenoxyethanol (2-POE), a semi-volatile surrogate selected for its representative behaviour compared to hazardous drugs.”
Add:
“Results are based on [X] replicate tests per device, with statistical comparisons made using [method].”

Response: Review #2 This change has been made to satisfy the reviewer #2 in another location in the conclusion however the author has overtly stated four replicates were performed in the abstract objectives; the abstract methods section and now added to the conclusion for additional clarity. Also n=4 made more overt in method section of the manuscript. Four replicates are stated clearly in the results section and in Table 4 and in the conclusions. The authors hope that this satisfies the reviewers requirements and thanks them for their suggestion.

INTRODUCTION
Flow & Logical Development:
The introduction builds up to the research question logically, starting with background on hazardous drugs, moving into CSTD classifications, then discussing prior protocols leading to the 2016 NIOSH draft.
Strengths:
• Solid foundation on the importance of CSTDs.
• Good explanation of the limitations of earlier testing methods.
• Clear rationale for adopting the 2016 NIOSH protocol.

Author Response: Review #2 This is valuable feedback thank you. Nothing more to add here.

Areas for Improvement:
• Repetition of content: Some paragraphs repeat similar points about IPA volatility and surrogate selection across different sections. Consolidate these ideas for better flow.

Response: Review #2 The reviewer points out there is repetition of the arguments over surrogate selection specifically around the use of IPA based on volatility. This is required when providing the rationale for using 2-POE as surrogate (see earlier comments above). Volatility of surrogates is an important theme and hence is repeated to ensure a clear rationale for selection of a semi-volatile surrogate for more representative containment data in the 2016 draft NIOSH protocol. The authors felt that it was necessary to reinforce these fundamental arguments based on the physicochemical properties of different surrogates used in both the 2015 draft NIOSH protocol (IPA) and in the 2016 draft NIOSH protocol (2-POE for example). The arguments that underpin surrogate selection need to be made because the use of semi-volatile as opposed to highly volatile surrogates (such as IPA) is a major plank of the progress made when developing the most recent update to the 2016 draft NIOSH test protocol. The authors believe that in a few different locations it was necessary to revisit the issue of IPA volatility because it is a major scientific limitation in the 2015 draft NIOSH protocol which the CDC NIOSH discuss in detail in the updated 2016 draft NIOSH protocol. Unfortunately since 2016 a number of publications have appeared in the literature that continue to employ the high volatility 70% IPA surrogate and this has led to much confusion in the scientific community. The authors have made significant efforts to reinforce the arguments made by the CDC NIOSH in why IPA is not relevant or could lead to erroneous and incorrect outcomes when used in the NIOSH protocol for CSTDs.

• Need for clearer transition: Between the discussion of surrogate selection and the actual methodology used in this study. A sentence such as “Given these considerations, we selected 2-POE at a concentration of 2.5% w/v…” would help bridge the gap.
Suggested Edit:
“While IPA has been widely used historically, its high volatility makes it unsuitable for accurately simulating the vapor release characteristics of most hazardous drugs. In response, the 2016 Draft NIOSH protocol proposed the use of semi-volatile surrogates, including 2-phenoxyethanol (2-POE). Based on these guidelines, we selected 2.5% w/v 2-POE as the challenge agent for our comparative assessment of four CSTDs.”

Author Response: Review #2 This is a reasonable request and the authors accept the suggested change of wording. The Authors have inserted the below paragraph to satisfy the reviewer comment and thank the reviewer for this suggestion.

“IPA has been widely used in recent publications [34-39] its high volatility makes it unsuitable for accurately simulating the vapor release characteristics of most hazardous drugs. In response, the 2016 Draft NIOSH protocol proposed the use of semi-volatile surrogates, including 2-phenoxyethanol (2-POE) [15]. Based on these guidelines and previous work published on the use of 2-POE by the UK Health and Safety Executive (HSE) [21,28], we selected 2.5% w/v 2-POE as the challenge agent for our comparative assessment of four CSTDs.”

METHODS SECTION
Overall Assessment:
The methods section is generally thorough and follows the 2016 Draft NIOSH protocol closely. However, several areas require clarification or expansion to meet the standards expected in a peer-reviewed publication.

Response: Review #2 The methods are taken directly from the cited and published work of CDC NIOSH for the 2016 draft NIOSH test protocol without any major changes. The methods are also cited in the previous publication by the same authors in Plos one (2018) reference [21] in the manuscript. For reasons of brevity the details are in supplementary materials and again there is a reference to this in the methods section for readers who wish to find this additional information.

Key Issues:
1. Surrogate Selection Justification
• What led to the choice of 2.5% w/v? Is this concentration within the range recommended by NIOSH or derived empirically?

Response: Review #2 The comment on surrogate selection is dealt with elsewhere by the authors in thie rebuttal document and is not covered here in detail.

Regarding the concentration of 2-POE selected and whether this is justified – this level of information is not provided by the 2016 draft NIOSH test protocol document. The concentration selected was derived empirically and is the highest concentration of this surrogate that could be achieved in aqueous solution without the solution reaching saturation. The authors have added a form of words to explain and justify the selected concentration for 2-POE. It is also a concentration at which a small volume of liquid release circa ~100 nL can be detected and quantified using the developed analytical methods allowing sufficiently good sensitivity of detection of small liquid releases of 2-POE. The Authors wish to thank the reviewer for their comment here and hope that this amendment satisfies this requirement.

The authors have made the following insertion of text in the methods section of the manuscript:

“The selection of a surrogate 2-POE concentration of 2.5% w/v has been discussed by the authors previously [21] and represents a clinically relevant concentration of hazardous drug whilst maintaining adequate solubility for the surrogate in aqueous solution.”

• Was there any pre-study compatibility testing with the tested CSTD materials? As noted in the literature (e.g., Simplivia white paper), some solvents can degrade tubing or seals.

Response: Review #2 This is an excellent point made by the reviewers and yes the authors have considered chemical compatibility issues for not only 2.5% w/v 2-POE but also a range of other proposed surrogate materials. Notably empirical studies have been performed (not reported by the authors in the present study) and these have found that there is no detectable incompatibility between a solutions containing 2.5% w/v 2-POE in aqueous solution and a variety of different plastics used in the construction of medical devices including those CSTDs tested in the manuscript. This is relevant at the concentration of 2.5% w/v 2-POE as used in the study. This is in contrast with the use of 70% IPA(aq) and 100% Ethanol, both proposed surrogates for NIOSH testing and both of which are contra indicating demonstrating significant chemical incompatibility with medical devices used in the preparation and administration of hazardous drugs. The authors thank the reviewer for this highly relevant and important point and hope that this satisfies the requirements.

Page 23 – changes are as follows in the methos section

“The selection of a surrogate 2-POE concentration of 2.5% w/v has been discussed by the authors previously [21] and represents a clinically relevant concentration of hazardous drug whilst maintaining adequate solubility for the surrogate in aqueous solution. It is also a concentration of surrogate that has been demonstrated to have no chemical incompatibility issues with different plastics employed in various medical devices including CSTDs. In contrast the authors have observed significant chemical incompatibility issues with a range of polymeric materials and medical devices when used in conjunction with the surrogate 70% IPA (unpublished data).”

2. Device Testing Protocol
• Were all manipulations performed strictly according to IFUs? If so, specify whether deviations were recorded or monitored.

Response: Review #2 IFUs were followed for each device with no deviations.

• Were negative controls included? For example, baseline chamber contamination levels before each test.

Response: Review #2 Yes baseline monitoring was performed as well as more general background monitoring to ensure sufficiently low levels of 2-POE had been achieved and were consistently attained before each test. This is stated in the methods.

3. Analytical Methods
• Clarify ATD-GC-MS parameters: What were the temperature settings, desorption times, column type, etc.? Without this detail, reproducibility is compromised.

Response: Review #2 This data is available in the supplementary materials and in the previous publication cited by the authors reference [21].

• Calibration curves: Were they generated fresh for each batch of samples? How was linearity confirmed?

Response: Review #2 Fresh calibration curves were run in full for each batch of samples tested and linearity of response was confirmed using the CDS software Chromeleon v7.3. which is a gold standard within quality control and quality assurance laboratories and is regulated by strict internal software algorithms/ rules.

4. Sample Size and Replication
• How many replicates were run per device? This is essential for interpreting statistical confidence in results.

Response: Review #2 This is stated and there were four replicates tested for each device. Due to the cost and complexity of conducting these type of tests we performed the required number of replicates as required by 2016 draft NIOSH protocol however in an ideal world it would have been nice to have obtained more replicate data for improved confidence. Where the data was always below LOQ there is no need or relevance for applying further statistical treatments to the data.

• Were outliers excluded? If yes, under what criteria?

Response: Review #2 No outliers were found and all data was included.

5. Statistical Methods
• Which statistical tests were used? T-tests, ANOVA, non-parametric equivalents?

Response: Review #2 Linear regression was used to calculate concentrations of 2-POE using algorithms within the CDS software Chromeleon 7.3 software. The line of best fit was applied that had the optimum regression coefficient and when calibrators were injected provided suitable levels of recovery (90-110%). As the data was below LOQ then further statistical treatments of the data was not appropriate or relevant. All data is reported as being below LOQ or more properly below LLOQ.

• Was multiple comparison correction applied? Given the number of pairwise comparisons, this should be addressed.
• Define what constitutes a statistically significant difference (e.g., p < 0.05).
Suggested Additions:
“All testing procedures were performed strictly in accordance with manufacturer instructions for use (IFU) to ensure validity. Deviations, if any, were documented and reviewed for potential impact on containment integrity.”
“To assess repeatability, five replicate tests were conducted for each CSTD model. Outliers were identified using the Grubbs’ test and removed only when statistically justified.”
“Chromatographic separation was achieved using a DB-5MS capillary column (30 m × 0.25 mm × 0.25 μm; Agilent Technologies) with helium as the carrier gas at a constant flow rate of 1.2 mL/min. Calibration was verified daily using freshly prepared standards.”

**Author Responses:**

As stated above the data always reported below LLOQ data and so further statistical treatment was not appropriate. No pairwise test was performed because the numbers are below LLOQ and so not meaningful.

The authors have added the following bullet point in the methods section:

“All testing procedures were performed strictly in accordance with manufacturer instructions for use (IFU) for each CSTD technology evaluated to ensure validity.”

The authors have referred the reader to the supplementary materials section for full instrument operational details such as temperatures, columns, flow rates etc. This information is supplied in full.

RESULTS SECTION
Strengths:
• Clear presentation of data.
• Tables summarize findings effectively.
• Statistical outcomes are reported where applicable.
Areas for Clarification:
• Figure/Table Correspondence: Ensure every figure and table is explicitly referenced in the text and that their captions are self-explanatory.

Author Response: The figures and tables are explicitly references in the manuscript.

• Units and precision: Make sure units (e.g., ppbv) are consistent throughout and that decimal places reflect measurement precision.

Author Response: The figures are consistent in units and precision reported.

Example:
“As shown in Table 2, ChemoLock™ barrier demonstrated significantly lower vapour leakage than Tevadaptor™ air-cleaning (p = 0.008).”

Author Response: The authors politely do not know where this data is cited from. The main results are presented in table 4. Also the releases are not just vapour but a total release from liquid, aerosol and vapour. All data is presented to 2 decimal places or three significant figures.

DISCUSSION
Strengths:
• Well-grounded in previous literature.
• Interpretation of results aligns with known properties of CSTD technologies.
• Acknowledges limitations of the current methodology.
Weaknesses:
• Overemphasis on protocol validation: While important, the discussion should center more on the implications of the observed differences among the CSTDs.

Author Response: The authors didn’t wish to over interpret the data because all devices demonstrated below LOQ release data. The authors do discuss the implications in terms of even the air-cleaning Chemolock CSTD providing equivalent containment performance to the physical barrier device of ChemoLock showing that the air-cleaning technology is equally effective.

• Limited interpretation of practical significance: Beyond statistical significance, what do the differences mean for healthcare worker exposure? Are the measured concentrations below regulatory thresholds?

Author Response: There are no regulatory thresholds for exposure to hazardous drugs and this is part of the wider problem when it comes to deriving acceptance criteria for CSTD technologies and the authors accept this limitation. However, the authors are using a highly sensitive analytical method and procedures and are able to demonstrate below quantifiable release performance from a range of CSTDs which supports their use when handling hazardous drugs. In the absence of any other containment data this is a step in the right direction. The data offers significant advantage over similar data reported using 70% IPA as surrogate because those data could reflect chemical incompatibility between the surrogate and the item under test. The fundamental requirement of any scientific experiment or test should be that the test conditions or equipment do not influence or effect the outcome of the experiment. In the case of 70% IPA this is not the case but in the submitted manuscript the authors have confidence that the containment data is robust and reliable and does reflect how these CSTD technologies would perform when used to handle hazardous drugs. This is a significant contribution to this area of scientific research.

Suggested Revisions:
“The observed differences in vapour containment among the tested CSTDs suggest that not all systems offer equivalent protection against occupational exposure. These findings support the need for standardised, performance-based evaluation methods such as the 2016 Draft NIOSH protocol.”
“Further research is needed to correlate laboratory-measured vapour leakage with real-world exposure scenarios to determine whether these differences translate into clinically meaningful reductions in risk.”

Author Response: The authors thank the reviewer for the above discussion and helpful suggestion of revision and has taken this onboard and offers the below revision:

“The observed differences in containment of 2-POE among the tested CSTDs suggest that not all CSTD technologies offer equivalent protection against occupational exposure. These findings support the need for standardised, performance-based evaluation methods such as the 2016 Draft NIOSH protocol.

Further research is needed to attempt to correlate laboratory-measured releases of a semi-volatile surrogate such as 2-POE with real-world exposure scenarios to determine whether these differences translate into clinically meaningful reductions in risk. The authors suggest that this type of investigation must be performed in a controlled laboratory environment where background levels of 2-POE can be controlled and monitored. One of the major limitations of hospital pharmacy or ward-based measurements of hazardous drugs from surface wipe studies is the absence of any controls and lack of reliability when attempting to assess the impact of an intervention such as a CSTD when this is against an unknown level of background surface contamination (including cross contamination) from hazardous drugs used in the hospital environment. As such it is theoretically possible to conduct a surface wipe intervention study for employing a CSTD versus no CSTD and obtain data showing that a CSTD increases exposure to hazardous drugs based on the amount of background cross-contamination on surfaces of devices handled in the hospital.”

The authors hope that the revision meets with the satisfaction of the reviewer.

CONCLUSION
Strengths:
• Directly addresses the research objective.
• Succinct and clear.

Suggestion:
Consider rephrasing the conclusion to emphasise the broader implication:
“These findings underscore the importance of adopting standardised, semi-volatile surrogates and quantitative testing methodologies to ensure reliable and comparable assessment of CSTD performance in protecting healthcare workers.”

Author Response: The authors wish to thank the reviewer for their valued contribution and suggested change and have added the below sentence to the final conclusion.

“These findings underscore the importance of adopting standardised, semi-volatile surrogates as provided by the 2016 draft NIOSH protocol such as 2-POE and quantitative testing methodologies to ensure reliable and comparable assessment of CSTD performance in protecting healthcare workers.”

FIGURES AND TABLES
General Observations:
• All tables appear clear and relevant.
• Figures (if present) must be checked for resolution and labelling consistency.
To Do:
• Confirm that all figures are labelled with axis titles, units, and legends.
• Ensure that statistical annotations (e.g., asterisks for p-values) are consistent and explained in the caption.

Author Response: this is done.

LIMITATIONS SECTION
A dedicated limitations section is missing. Include one after the discussion to address:
• Potential influence of surrogate concentration on device behaviour.

Author Response: this is done.
• Absence of real-world validation.

Author Response: this is done.
• Limited scope of CSTD models tested.

Author Response: this is done.
• Possible variability in operator technique despite adherence to IFUs.

Author Response: this is done.

We the authors would like to once again thank the reviewers for their time and attention and confirm that all of the reviewers questions have been addressed in full and believe that the work described is now in a suitable form for consideration for publication as an original research article and this manuscript is approved by all authors for publication, has not been previously published, and is not under consideration for publication by another journal.

Thank you for your kind consideration.

There have been no significant developments in the area which this manuscript addresses since its original submission in 2022 and there is still no update from NIOSH on the finalization of the draft 2016 NIOSH protocol.

However, there is considerable confusion within the pharmacy community because the earlier draft 2015 NIOSH protocol is still available and being used to assess the vapour containment of CSTDs. There is a growing body of evidence that demonstrates a contra-indication between 70% IPA, the surrogate employed in the 2015 draft NIOSH protocol and a wide range of medical devices used in administration of oncolytic drugs which results in failure of those devices.

It is therefore the opinion of the authors that it is increasingly important to provide robust scientific evidence of an alternative test protocol such as the 2016 draft NIOSH protocol in conjunction with valid semi-volatile surrogates such as 2-POE with robust assessment for additional commercially available CSTDs. This is the purpose of the submitted revised manuscript.

Sincerely,

Alan-Shaun Wilkinson Ph.D

Biopharma Stability Testing Laboratory Ltd.

BioCity Nottingham,

Innovation R3,

Pennyfoot Street

Nottingham, NG1 1GF

United Kingdom

Email: [alan@biopharmatesting.co.uk](mailto:alan@biopharmatesting.co.uk)

Tel: (+)447841204528

**Financial Disclosure Statement:**

The author ASW received funding from ICU medical (SA) to support the research study including the supply of the commercially available closed system devices that were tested as part of the study.

"The funders had no role in study design, data collection and analysis, decision to publish, or preparation of the manuscript."
