# Supplementary material for: Head-to-head containment performance testing of four commercially available closed-system transfer devices using the 2016 Draft NIOSH test protocol and 2.5% w/v 2-phenoxyethanol as challenge agent
Source: PLoS One. 2026 Nov 12;21(11):e0337729. doi: 10.1371/journal.pone.0337729 (PMC13759118; doi:10.1371/journal.pone.0337729)
Supplement: Supplementary file 4 [file pone.0337729.s005.docx]

PONE-D-25-12716R1

Head-to-head containment performance testing of four commercially available closed-system transfer devices using the 2016 Draft NIOSH test protocol and 2.5% w/v 2-phenoxyethanol as challenge agent.

Dear PLOS One

Please find below the authors rebuttal responses to reviewers questions for the above manuscript.

Please find included the following items and files:

- A letter that responds to each point raised by the academic editor and reviewer(s). This letter is a separate file labelled 'Response to Reviewers'.
- A marked-up copy of the latest manuscript that highlights changes made to the original version. This file is labelled 'Revised Manuscript with Track Changes'.
- An unmarked version of the revised paper without tracked changes. This is a separate file labelled 'Manuscript'.

**Authors Responses to Reviewers Questions:**

**Comments to the Author (ASW)**

1. If the authors have adequately addressed your comments raised in a previous round of review and you feel that this manuscript is now acceptable for publication, you may indicate that here to bypass the “Comments to the Author” section, enter your conflict of interest statement in the “Confidential to Editor” section, and submit your "Accept" recommendation.

Reviewer #2: All comments have been addressed

Reviewer #3: (No Response)

Reviewer #4: All comments have been addressed

Reviewer #5: All comments have been addressed

Reviewer #6: All comments have been addressed

ASW Response: Nothing to address here.

2. Is the manuscript technically sound, and do the data support the conclusions?

The manuscript must describe a technically sound piece of scientific research with data that supports the conclusions. Experiments must have been conducted rigorously, with appropriate controls, replication, and sample sizes. The conclusions must be drawn appropriately based on the data presented.

Reviewer #2: Yes

Reviewer #3: (No Response)

Reviewer #4: Yes

Reviewer #5: Yes

Reviewer #6: Yes

ASW Response: Nothing to address here.

3. Has the statistical analysis been performed appropriately and rigorously?

Reviewer #2: Yes

Reviewer #3: (No Response)

Reviewer #4: Yes

Reviewer #5: Yes

Reviewer #6: No

ASW Response: Nothing to address here. Reviewer 6 has responded No. The author respectively would like to respond to reviewer 6 to say that as all recorded values for 2-POE concentration were below the lower limit for quantitation (<LLOQ) then there is no valid statistical treatment of the data possible. The only statistical analysis that is pertinent and has been followed and included by the authors is the fitting of the analytical calibration data for 2-POE using linear regression analysis.

4. Have the authors made all data underlying the findings in their manuscript fully available?

The [PLOS Data policy](http://www.plosone.org/static/policies.action#sharing) requires authors to make all data underlying the findings described in their manuscript fully available without restriction, with rare exception (please refer to the Data Availability Statement in the manuscript PDF file). The data should be provided as part of the manuscript or its supporting information, or deposited to a public repository. For example, in addition to summary statistics, the data points behind means, medians and variance measures should be available. If there are restrictions on publicly sharing data—e.g. participant privacy or use of data from a third party—those must be specified.

Reviewer #2: Yes

Reviewer #3: (No Response)

Reviewer #4: Yes

Reviewer #5: Yes

Reviewer #6: Yes

ASW Response: Nothing to address here.

5. Is the manuscript presented in an intelligible fashion and written in standard English?

PLOS ONE does not copyedit accepted manuscripts, so the language in submitted articles must be clear, correct, and unambiguous. Any typographical or grammatical errors should be corrected at revision, so please note any specific errors here.

Reviewer #2: Yes

Reviewer #3: (No Response)

Reviewer #4: Yes

Reviewer #5: Yes

Reviewer #6: Yes

ASW Response: Nothing to address here.

6. Review Comments to the Author

Please use the space provided to explain your answers to the questions above. You may also include additional comments for the author, including concerns about dual publication, research ethics, or publication ethics. (Please upload your review as an attachment if it exceeds 20,000 characters)

Reviewer #2: Thank you for addressing the comments. You have thoroughly addressed all prior concerns with clear, thoughtful revisions that significantly strengthen the manuscript. The introduction now provides a more cohesive rationale for the study, and the methods section has been expanded with essential technical details—particularly regarding analytical procedures, replication, and statistical analysis—ensuring reproducibility. The inclusion of a dedicated limitations section adds necessary context to the findings, enhancing transparency and scientific rigor. Clarifications around surrogate selection, data handling, and device testing protocols align well with PLOS ONE’s standards. The discussion appropriately interprets results in light of real-world implications for healthcare worker safety and CSTD performance evaluation. All figures and tables are now accurately referenced and clearly presented, with consistent units and annotations. Most importantly, the authors have maintained a balanced, evidence-based tone throughout, avoiding overstatement while still highlighting the study’s value in enabling direct comparison between air-cleaning and barrier CSTD technologies. The financial disclosure and competing interests statements are now fully transparent, confirming that the funders had no role in study design or data interpretation. These revisions have transformed the manuscript into a robust, well-executed study that makes a valuable contribution to occupational safety in healthcare settings

Author Response: Thank you Reviewer 2 for your thoughtful and balanced review of the manuscript and recent changes and for recommending this to go forward for publication. We appreciate your time in reading the revised manuscript and diligence in review.

Reviewer #3: This manuscript presents a head-to-head containment performance comparison of four commercially available Closed System Transfer Devices (CSTDs) using the 2016 Draft NIOSH test protocol and 2.5% w/v 2-phenoxyethanol (2-POE) as a surrogate. The study is methodologically sound, builds upon previously published and NIOSH-adopted protocols, and provides quantitative containment data for both physical barrier and air-cleaning CSTDs. The conclusions are well-supported by the data. However, the manuscript is excessively long and repetitive, particularly in the Introduction and Discussion sections. Several clarifications and improvements in data presentation, statistical reporting, and focus are needed before publication in this journal.

Author Response: Thank you Reviewer 3 for your balanced responses which are very supportive of this being a scientifically robust piece of research. Reviewer 3 comments regarding the length of the manuscript are on point and 100% aligned with a reviewer in the previous round of peer review. Unfortunately, although the excessive and repetitive sections were removed especially in the introduction and discussion sections along with clarifications and improvements in data presentation the version with these changes submitted does not appear to have been reviewed. The attached is a re-submission and so hopefully all of the changes that should have been included last time are now completed in this submission. We would like to thank you again for raising this point because it allowed us to identify this issue.

As mentioned above in our rebuttal to Reviewer 6 in terms of statistical reporting there is no appropriate statistical treatment that can be applied to the results data as all data is below the limit of quantitation (<LLOQ) and as such the numbers cannot be subject to any further statistical treatment. All results data were found to be below the limit at which the instrument can attribute a value using the external calibration curve. The author wishes to thank the reviewer once again for their time and consideration in reviewing the revised article.

Introduction
The introduction is generally sound but could be more focused. Currently, it provides extensive background on general populations before narrowing to the target group. I suggest tightening the narrative to more directly foreground the study population and research gap. Additionally, please incorporate recent, relevant references to strengthen the theoretical foundation, such as:

Author Response:

The author appreciates the reviewer recommendations and the revised manuscript has now been tightened up with significant removal of surplus text and discussion. We hope that this satisfies the reviewer. The below recommended articles suggested by the reviewer to strengthen the theoretical foundations are mostly all relating to Iranian oil field work and are not relevant to the subject matter for the submitted manuscript. One is related to shift work related disorders in a hospital and is therefore not considered relevant to the subject of the manuscript.

DOI: 10.18502/ijph.v52i10.13854

Iran J Public Health. 2023 Oct;52(10):2157-2168. doi: 10.18502/ijph.v52i10.13854.

Workplace Risk Factors Assessment in North-Azadegan Oil Field Based on Harmful Agents Risk Priority Index (HARPI)

DOI: 10.3923/rjmsci.2016.111.115

Phenol Removal from Aqueous Environment by Adsorption onto Pomegranate Peel Carbon

November 2016Electronic physician 8(11):3248-3256

DOI: 10.3233/WOR-220077

**Semi-quantitative risk assessment for workers exposed to occupational harmful agents in an oilfield in Iran**

doi: 10.3233/WOR-203165

Shift working disorders among nurses of Tehran hospital and its related factors in 2016

https://www.makhillpublications.co/public/files/published-files/mak-rjas/2016/7-547-553.pdf

Relationship between Job Content and Demographic Variables with Musculoskeletal Disorders among Nurses in a University Hospital, Qom Province, 2014

In summary the authors thank the reviewer for their input and recommendations and have tightened up the submitted manuscript but have decided not to include the suggested references on the grounds that they are not relevant to the subject matter.

At the continue 10 major and minor comment. : Major Comments
\### **10 MAJOR COMMENTS**

1. **Excessive Length & Repetition**
The manuscript is unnecessarily long (over 120 pages), with significant repetition, especially regarding the rationale for surrogate selection and criticisms of the 2015 NIOSH protocol. The authors should consolidate repetitive sections (e.g., surrogate selection arguments) and move detailed methodological descriptions to Supplementary Materials.

Author Response: The revised manuscript has been significantly shortened with large sections of text and discussion removed especially on surrogate selection. The author previously made these alterations but for some reason the long version was reviewed. This is now addressed fully in the revised manuscript.

Hopefully the revised text meets the reviewers expectations for reduced word count and is now less than 30 pages in length. The author has removed details on surrogate selection and the critique of the 2015 draft NIOSH protocol. The authors view remains that as the 2015 Draft NIOSH protocol related publications are increasing at pace it is important to highlight the lack of scientific rigour and inability of this protocol to quantify releases of Isopropyl alcohol as surrogate because the results generated by researchers employing this protocol are meaningless and voided. This is actually quite dangerous for those making decisions based on containment performance from the 2015 Draft NIOSH test protocol. That said the author is happy to remove this critique and it is now removed from the manuscript.

There is some contradictory feedback between this reviewer and other reviewers with respect to placement of additional details for methodology with some reviewer comments requesting more detail within the main body of the article and some asking for this to be placed in supplementary materials which is where most of the details sit currently. The author view is that there is a good balance in detail supplied in the main body of the article to enable reproduction of the testing with references to additional sources of information included and the supplemental materials. It would be impossible to please all of the reviewers on this point but I hope that I have satisfied the current Reviewer in achieving the correct balance of detail for the methodology and thank them once again for their input here.

2. **Lack of Clear Research Hypothesis & Objectives**
While the objective is stated in the Abstract, the main text does not clearly articulate a testable hypothesis. The study appears descriptive rather than hypothesis-driven. A clearer framing of the research question would strengthen the paper.

Author Response: The author thanks the reviewer for their opinion and view on the strength of the objective however the objectives are clearly set out as being a head to head comparison of the containment performance of four commercially available closed system transfer devices which is very clear. The hypothesis is simply can each type of tested CSTD device contain the surrogate 2-POE. I do not think that this needs to be re-framed and this would not improve the article. The null hypothesis for the study is that the CSTDs under test are not able to completely contain the 2-POE surrogate whilst carrying out NIOSH Task 1 and Task 2 which is obvious. Also due to the word count restriction adding in more word is simply not an option without removing key aspects of the work. On balance we have left it as it was. I hope that this is acceptable.

3. **Insufficient Statistical Detail**
The authors state that “no statistically significant difference was found,” but they do not report the specific statistical tests used, p-values, or effect sizes. Since all results were below the LOQ, the appropriateness of statistical comparisons should be justified or clearly stated that formal testing was not performed.

Author Response: The author thanks the reviewer for this comment and I believe is suggesting as does the author that as all values were recorded as being below LLOQ then there is no appropriate statistical treatment that can be applied to the results data. It is therefore fair to state that there was no difference found between the containment performance of the two types of CSTD evaluated because they both gave below LLOQ values. The only statistical tests employed in the study were linear regression for the calibration curve for 2-POE response versus amount and calculation of means of each cohort of replicates for each CSTD and NIOSH Task. Any other test such as ANOVA would only have been appropriate if the test data had produced above LLOQ i.e above quantifiable limits release data.

4. **Data Availability Statement is Inadequate**
The authors claim data are available “within the manuscript and Supporting Information,” but the raw instrument data (Chromeleon files) are not accessible. This violates PLOS ONE’s data policy. They should deposit raw data in a public repository or provide processed data in a usable format (e.g., CSV).

Author Response: The author thanks the reviewer for this comment. The Author has made all processed data available in the manuscript and as the reviewer has pointed out proprietary data handling software in Chromeleon CM7.3 (very high cost) was used to collect and process the data. This software is proprietary and is designed to work within a highly regulated laboratory environment under strict data handling protocols. As such it is not possible to access the RAW instrument data which would also be extremely difficult to handle or file transfer due to the extremely large file sizes involved in CDS mass spectrometry data. Attempts to copy the data may also lead to the possibility of corruption of the RAW data or inclusion of “mistakes” and this might lead to inaccuracies and for third party researchers to then perform compromised analysis on the compromised data. All processing was done using the internal algorithms inside of Chromeleon CM7.3 which is an industry standard rather than in third party programs such as excel. The authors therefore believe that sufficient sharing of the CDS processed data has performed and the data as it is, has been made available and put in the public domain in accordance with Plos one data policy. Also given that the data provides limited opportunity for re-processing due to it being below LLOQ and in many cases below LOD the lack of RAW data is not disadvantaging the scientific community engaged in this type of work from benefiting from the data. The authors have also checked all of the main scientific data repositories and the only ones available do not accept Chromeleon data files or GCMS data files unless linked to metabolics studies which are able to be filed.

5. **Overemphasis on Protocol Validation**
A large portion of the Discussion is devoted to defending the 2016 NIOSH protocol and critiquing other methods. While context is important, the focus should shift toward interpreting the *results* of this study and their implications for CSTD selection and healthcare worker safety.

Author Response: The author thanks the reviewer for this comment. This has been addressed and the text removed including any critique of the earlier 2015 Draft NIOSH test protocol and a focus more on the actual study results data and implications for healthcare workers. We would like to thank the reviewer for their comment on this aspect of the manuscript.

6. **Limited Discussion of Practical Significance**
The clinical or occupational health implications of the findings are underdeveloped. What does “below LOQ” mean in terms of real-world exposure risk? How should end-users interpret these results when selecting a CSTD?

Author Response: The author thanks the reviewer for this comment which makes a valid point. Unfortunately it is impossible for the author to comment on the exposure risk to healthcare workers when below LLOQ concentrations of 2-POE (the surrogate for hazardous drug materials) are reported. The fact that we do not yet have instrumentation capable of detecting at significantly below parts per billion volume levels (ppbv) does not mean that these levels could not be causing harm. We simply do not know. The key conclusions the authors make are that with the four different CSTDs evaluated in the study they all gave below LLOQ results and so can be regarded as being equally capable of offering a level of protection to healthcare workers when the CSTDs tested are used with hazardous drugs as compared with an open system of needle and syringe. We thank the reviewer for this very thought provoking commentary and on another platform/ forum, if word count and circumstance allowed there should be a larger discussion around what these studies mean in terms of protection and real-world exposure risk involving hazardous drugs. The authors are keen to point out that there is a good rationale for using a semi-volatile material such as 2-POE to mimic hazardous drugs because it exhibits similar behaviour to other semi-volatile materials when they are released in liquid droplet form (which arguably represents the highest risk in terms of potential exposure). Unfortunately, because the hazardous drugs have such low chemical concentration in the vapour they are currently impossible to detect directly in the vapour using the NIOSH approach. By using a surrogate approach with a safety factor of 100 if we are detecting 1ppbv of the surrogate then in theory that might mean the hazardous drug release resulting from a similar release would be one hundredth of this value or 10 parts per trillion volume but this is a leap of faith and we cannot be sure that that would be the case. In simple terms if there is 2-POE detected from release when using the CSTD and this is above the lower limit for quantification (LLOQ) then we can compare this with releases when using an open system of needle and syringe which are also well above the LLOQ for 5 microlitre and 10 microlitre release volumes. This allows expression of how much better the CSTD is doing relatively compared with not using a CSTD or having an open system. When all of the release values are below the LLOQ then we cannot really comment except to say that the CSTD should offer significant improvement in operator protection as compared with an open system of needle and syringe – providing the hazardous drug materials behave in an analogous manner to the surrogate. The caveat at the end is important for surrogates that are very volatile because these materials do not behave analogous to hazardous drugs and releases involving isopropyl alcohol or ethanol will evaporate very quickly and achieve 100% of their theoretical saturated vapour concentration whereas we know that materials that are semi-volatile will not achieve much more than ~1% of their SVC. This could over-estimate the occupational risk when using volatile surrogates by another factor of 100 over the safety factor of 100 making the interpretation of results in terms of occupational exposure less meaningful. For liquid releases the exposure risk to healthcare workers when handling hazardous drug materials is from direct contact with the released material and absorption through the skin or secondary transfer followed by dermal exposure.

7. **Sample Size Justification**
The use of n=4 replicates is based on the NIOSH protocol but is not statistically justified. A power calculation or justification for the sample size should be provided, especially given that all results were below LOQ.

Author Response: The author thanks the reviewer for this interesting comment. The authors are not attempting to justify the sample size as this is taken directly from the 2016 Draft NIOSH universal test protocol. The authors study design was to follow this test protocol which we did. The difficulty in performing larger numbers of tests is that the testing would become prohibitively expensive to perform for third party researchers such as university students and one of the purposes of dissemination of the work is to allow a greater number of scientific researchers access to the protocols and methodology to perform their own independent testing. The authors do not think that a power calculation would be meaningful in this context and would break the link with performing the testing in accordance with the requirements of the 2016 Draft NIOSH universal test protocol. A further point is that as the number of tests increase the number of days over which the testing is performed increases and the background levels of various semi-volatile and volatile materials may fluctuate to a greater extent when increasing the overall time line for testing. This would impact the quality of the data and we might observe an increased trend in background when performing much larger replicates of tests in a single cohort. This can be observed in the 2018 publication in Plos one by the same authors, here the LLOQ level was almost twice that obtained in the more recent submitted study. This is an unintended consequence of higher background levels of 2-POE that are so subtle that they are not detected in the background performed immediately before test but that do contribute to an increase in the LLOQ (here by a factor of 2!). With an increased LLOQ it may prevent discrimination between two different CSTDs, one that is just above the LLOQ and one that remains below the LLOQ and so controlling the background levels of the surrogate 2-POE become incredibly important in this type of testing. The authors validated a cleaning protocol and performed robust cleaning of the NIOSH chamber in between each test but simply the fact that many more tests were performed around the time of the 2018 Plos one publication testing the LLOQ for 2-POE was double that presented in the current article.

8. **Incomplete Reporting of Methodological Details**
Key details (e.g., ATD-GC-MS parameters, column temperatures, desorption times) are relegated to Supplementary Materials but are critical for reproducibility. Essential parameters should be summarized in the main Methods section.

Author Response: The author thanks the reviewer for this comment. Please see above the justification for putting all of the details of the instrument program in the supplementary file. The article is already 30 pages in length and it is appropriate to include the more basic experimental detail in the main body of the methods section for such complex analyses. You will find all of the details for running the chromatography method in the supplementary file attached. Temperatures, gas flow rates etc.

9. **Potential Conflict of Interest**
The study was funded by ICU Medical, the manufacturer of two tested devices (ChemoLock™). Although the authors state the funder had no role in study design or analysis, the potential for bias should be more transparently discussed in the Limitations section.

Author Response: The author thanks the reviewer for this comment. The authors are experienced academics and are used to working on projects that have external funding. Funders never have anything to do with the studies. Funders also expect and demand that this is the case because they value the independence of the work being carried out by an external group otherwise they would simply perform the study internally. As with most scientific research projects there was some financial assistance from a commercial third party, but they did not have any input in the study design, execution, data analysis, or writing of the manuscript. The authors would also like to point out that the findings demonstrate equivalency of containment performance to other competitor devices which is not in the interests of the named commercial third party. I think this should resolve any concerns the reviewer might have in terms of bias.

10. **No Dedicated Limitations Section**
The manuscript lacks a structured Limitations section. Important limitations include the use of a single surrogate concentration, the absence of real-world validation, the small number of CSTD models tested, and the lack of exposure-risk correlation.

Author Response: The author thanks the reviewer for this comment. The experimental work presented follows on from work previously published in 2018 using 2-POE and more extensive work performed using the surrogate of 2-POE in water by the UK Health and Safety Executive (HSE) which is cited in the submitted manuscript. The choice of the single concentration of 2-POE is not seen as a limitation of the study. It does not limit the capability to detect microlitre and nanolitre scale liquid releases of surrogate and airborne chemical concentrations down to below one parts per billion volume (ppbv) which is state of the art for detection of semi-volatile materials in the air/ vapour. A key study design rule was not to exceed or approach the limit of solubility of the surrogate 2-POE but to provide highly sensitive measureable chemical vapour concentrations from microlitre scale liquid releases inside the NIOSH chamber down to sub parts per billion volume (ppbv) levels which is the state of the art in terms of both selectivity and sensitivity. Higher concentrations of the surrogate 2-POE may also cause incompatibility issues with the equipment under test just as 70% isopropyl alcohol has been observed to damage many different types of medical grade plastics used in the construction of medical devices. We have done some research (unpublished data) on this aspect and not found compatibility issues with 2-POE and CSTDs over a range of concentrations but have found serious issues with 70% IPA and other previously proposed NIOSH surrogates and so it is a key consideration when selecting the concentrations of a surrogate for use in NIOSH testing. A key experimental design rule is that you do not want the test surrogate to have an impact on the outcome of the testing – is should be inert and therefore play no part or influence the results. The authors are confident that at 2.5 % w/v 2-POE is a suitable surrogate, remains inert and provides robust scientific data in containment performance testing of CSTDs. It is also a relevant concentration in terms of drug concentration in aqueous solution. 70% IPA has however been proven to interact in deleterious ways with medical device equipment during testing and is therefore not a preferred challenge agent to use in testing of CSTDs.

In terms of exposure risk correlation, it is beyond the scope of the study to attempt to correlate the release data for 2-POE to exposure of healthcare workers when handling hazardous drugs. The study is aimed to demonstrate the performance in containment of two different types of CSTD when using 2-POE as surrogate in the 2016 Draft NIOSH universal test protocol which it does. There is a design safety factor of 100 in terms of theoretical SVC for 2-POE over thiotepa the most volatile hazardous drug and the relationship between surrogate and hazardous drugs in terms of theoretical SVC can provide a “best guess” at what a hazardous drug concentration might be in the vapour from a similar size liquid release of hazardous drug if you are able to achieve above LLOQ releases with 2-POE. With the study data we were always below LLOQ.
---

\### **10 MINOR COMMENTS**

1. **Abstract Clarity**
The Abstract should briefly state *why* 2-POE was chosen as the surrogate. Add one sentence on its semi-volatile properties and relevance to hazardous drugs.

Author Response: The author thanks the reviewer for this comment. We were previously asked by a reviewer to remove discussion around selection of 2-POE. Further, the choice of 2-POE is detailed in the 2016 Draft NIOSH universal test protocol for the reader to access if they wish to. The manuscript also builds on a previous publication and works using 2-POE in combination with the 2016 Draft NIOSH universal test protocol for assessment of containment performance and this is cited by the authors and provides the relevant justification for selection. The authors also discuss in the main body of the manuscript the work by Pengelly and Wheeler et al at the Health and Safety Executive (HSE) using 2-POE as a surrogate for hazardous pesticides in real-world experiment scenarios which again points in to the work already done to demonstrate the strengths of using semi-volatile material such as 2-POE as surrogate.

2. **Figure & Table Referencing**
Some figures (e.g., Figure 3) are not clearly referenced in the text. All figures and tables should be explicitly cited in the order they appear.

Author Response: The author thanks the reviewer for this comment. All figures are clearly referenced in the text. We apologise if you may be looking at an earlier version of the manuscript but this I can assure you has been fully addressed.

3. **Typos and Inconsistencies**
- Page 75, Conclusions: “NIOSH Task 2each” → “NIOSH Task 2 each.”
- Page 96: “was-based-on It is” → “It is.”
- Page 107: “In feetfact” → “In fact.”

Author Response: The author thanks the reviewer for this comment on typographical errors and these have been addressed in the latest manuscript.

4. **Units Consistency**
Use “ppbv” or “ppb” consistently. In some places, “ppb” is used without specifying volume.

Author Response: The author thanks the reviewer for this comment on typographical errors and these have been addressed in the latest manuscript. All changed to ppbv and LLOQ.

5. **Positive Control Reporting**
Positive controls were performed at 5 and 10 µL, but the text references an ability to quantify down to ~100 nL. Clarify the relationship between the reported LOQ (0.41 ppb) and the minimum quantifiable release volume.

Author Response: The author thanks the reviewer for this comment. The system suitability tests were performed at 5 microlitre and 10 microlitre volume levels as these are microlitre size releases and so are at an appropriately small level for demonstrating detection sensitivity and are practicable to be performed. The performance of the analytical method is subject to the background level of 2-POE analysed prior to each test (in duplicate), the chamber background values (n=85) were processed using standard methodology and used to calculate the LLOQ of 0.41 ppbv. This chamber concentration can then be directly related to a microlitre or nanolitre scale liquid release of 2-POE surrogate which equates to a 100 nL release at this concentration level.

6. **Reference Formatting**
Some references are incomplete (e.g., Ref. 30–33 are “private communication” or presentations). Replace or cite formally where possible.

Author Response: The author thanks the reviewer for this comment. There is no formalised way of citing these presentations/ communications.

7. **Repetitive Language in Introduction**
The Introduction repeats points about USP <800> and NIOSH protocols. Streamline to improve flow.

Author Response: The author thanks the reviewer for this comment and the manuscript has been streamlined considerably in terms of removing repeated comments and sections.

8. **Conclusion Restructuring**
The Conclusion is lengthy and reiterates the Discussion. Condense to a clear summary of findings, implications, and future research.

Author Response: The author thanks the reviewer for this comment. The conclusion has been revised and presents a concise summary of the key experimental data.

9. **Definition of LOQ/LLOQ**
The terms LOQ and LLOQ are used interchangeably. Use one term consistently.

Author Response: The author thanks the reviewer for this comment on typographical errors around LLOQ and LOQ and these have been addressed in the latest manuscript.

10. **Acknowledgments Section**
The funding statement is repeated in multiple places. Consolidate into a single Financial Disclosure section.

Author Response: The author thanks the reviewer for this comment. This has been addressed.
---

**Recommendation**
**Major Revision Required.** The manuscript presents valuable and robust containment data for CSTDs using an advanced NIOSH protocol. However, it requires significant condensation, improved statistical reporting, clearer data availability, and a sharper focus on the study’s findings and implications before it is suitable for publication in this journal.
Discussion Depth
The current discussion is somewhat superficial. Please provide a more in-depth interpretation of findings in relation to existing literature, integrating the updated references above.
I look forward to reviewing the revised manuscript. Please address all points comprehensively in your response letter and manuscript track changes.
Please update and ensure accurate, complete citations with DOIs. Incorporate recent literature to strengthen the discussion, such as:

DOI: 10.18502/ijph.v52i10.13854
DOI: 10.3923/rjmsci.2016.111.115
DOI: 10.3233/WOR-220077
doi: 10.3233/WOR-203165
---

Author Response: The author thanks the reviewer for this comment however the recommended citations are not relevant to this study. The author believes that all other matters have been addressed critically reduction in word count and length with removal of large sections of text and discussion and tightening of the manuscript to appeal to a wider readership.

Reviewer #4: The article mentions: "No care over of 2-POE was observed in the chamber following testing, and there is no additional cleaning of the chamber was required between tests”
However, there is no clear explanation here on how to verify 'no residue'. Is it through visual inspection, cabin air sampling, or surface wiping? If air sampling is used, is the detection limit low enough to ensure that there are no low-level residual effects on subsequent testing?
Improvement suggestion: Can you briefly explain the specific method for verifying "no residue" (e.g. "verifying by sampling the cabin with standard air and confirming that the 2-POE concentration is below the detection limit after testing") to enhance the credibility of this statement.

Author Response: The author thanks the reviewer for this comment. The stated detection limit (LOD) of the instrumental method demonstrates that the chamber blank samples were consistently below this figure and therefore acceptable. The authors did not observe an increase in chamber backgrounds, LOD or LLOQ with increasing numbers of tests which demonstrates that there is not an accumulation or carry over of 2-POE between tests.

Reviewer #5: Comments to the Author
1. Abstract
• The abstract clearly states objectives, methods, and key findings. However, it would benefit from briefly noting why 2.5% w/v 2-POE was selected over other surrogates, reinforcing the methodological rigor.

Author Response: The author thanks the reviewer for this comment but there is no room in the word count to go into selection criteria for the surrogate 2-POE. This could not be covered in a brief sentence. The author believes this aspect of the study design is covered in the main body of the manuscript as well as previous publications including from the same authors 2018 (Plos One) and in the 2016 Draft NIOSH Universal test protocol for containment performance of CSTDs published by the NIOSH. For brevity it is kept out of the abstract.

• Inclusion of “n=4 replicates” is appropriate, but specifying that results were below LOQ for all devices quickly highlights the high containment performance observed.

Author Response: The author thanks the reviewer for this comment but is unsure of the point the reviewer is trying to make here.

2. Introduction
• The background effectively establishes the importance of CSTDs and the evolution of NIOSH protocols. However, the section could be tightened to reduce repetition regarding surrogate volatility.
• The rationale for selecting 2-POE over IPA is well-supported by references to HSE and NIOSH documents, strengthening the scientific premise.
3. Methods
• The adherence to the 2016 NIOSH draft protocol and manufacturer IFUs is a strength, ensuring consistency and validity.
• Clarification on the chemical compatibility testing of 2.5% w/v 2-POE with CSTD materials would enhance methodological transparency.

Author Response: The author thanks the reviewer for this comment on compatibility, but the authors felt that this was outside of the scope of the manuscript. The compatibility aspect of surrogates is something that the NIOSH has covered in its town hall meeting in 2016 where ICU medical presented data on this demonstrating the compatibility of 2-POE with the plastics used in CSTD medical devices.

• While replicate number (n=4) is stated, a brief justification for this sample size in relation to statistical power would be useful.

Author Response: The author thanks the reviewer for this comment but the authors are simply replicating the protocols published by the NIOSH and are not making a comment on the appropriateness of the number of replicates within the protocol.

4. Results
• Data presentation in Table 4 is clear, showing all devices performed below LLOQ. The use of confidence intervals adds robustness.
• Positive and negative control results suitably demonstrate system suitability and low background interference.
• The ability to quantify releases down to ~650 nL is a notable technical achievement and should be emphasized as a key outcome.
5. Discussion and Conclusion
• The discussion appropriately interprets the parity between air-cleaning and barrier CSTDs, a significant finding for OHS practice.
• However, the practical implications for healthcare worker exposure risk are underdeveloped; linking LOQ values to potential hazard thresholds would strengthen relevance.

Author Response: The author thanks the reviewer for this comment on linkage between the study findings using 2-POE to assess containment performance and healthcare worker exposure risk. The authors believe that this discussion sits outside of the objectives of the study and manuscript but thanks the reviewer for raising this thought-provoking point. We are happy to report that of the four types of CSTD assessed in the study we could not quantify any release of 2-POE indicating good levels of containment of all devices tested.

• The conclusion rightly advocates for standardized semi-volatile surrogate use, but could more explicitly call for NIOSH to finalize the 2016 protocol based on this evidence.

Author Response: The author thanks the reviewer for this comment and it is a very good point indeed but the authors were keen to not get involved in any politics and stay within the scientific parameters and focus on the key findings of the study. We do agree that it would be extremely helpful if the NIOSH were to finalise a protocol and bring some clarity to the area. We in the mean time are happy to attempt to bring some robust scientific data to the pharmacy and nursing communities to help inform them when choosing an appropriate CSTD technology in their work flow and clinical practice.

6. References
• References are comprehensive and appropriate, covering key regulatory documents and prior research.
• Including a citation for the chemical compatibility claim between 2-POE and medical device plastics would further support the methods.

Author Response: The author thanks the reviewer for this comment. The author has added in a reference on chemical compatibility with different organic solvent materials.

Final Recommendation
Minor Revisions. The manuscript presents a technically sound, well-controlled study that advances the evaluation of CSTD containment performance. The methodology is robust, data support the conclusions, and the work has direct relevance to occupational health practice. Revisions should focus on refining the discussion of practical implications, clarifying methodological justifications, and minor editorial tightening to enhance clarity.

Author Response: The author thanks the reviewer for their extensive and comprehensive comments.

Reviewer #6: Ref.: PONE-D-25-12716R1
Title:
Head-to-head containment performance testing of four commercially available closed-system transfer devices using the 2016 Draft NIOSH test protocol and 2.5% w/v 2-phenoxyethanol as challenge agent
Dear editor
Greetings
Thank you for giving me the opportunity to review this manuscript. Thanks to the valuable efforts of the authors. This study is a practical study whose findings can lead to proper awareness for the society. This manuscript presents a head-to-head comparison of four closed-system transfer devices (CSTDs)—two physical barrier types (ChemoLock™ barrier and BD PhaSeal™) and two air-cleaning types (ChemoLock™ air-cleaning and Tevadaptor™)—using the 2016 Draft NIOSH universal protocol for assessing containment performance during hazardous drug compounding and administration. The challenge agent is 2.5% w/v 2-phenoxyethanol (2-POE) in water, analyzed via automated thermal desorption gas chromatography-mass spectrometry (ATD-GC-MS). The study concludes that all devices perform below the limit of quantification (LOQ; 0.41 ppb), with no significant differences between barrier and air-cleaning types, suggesting equivalent containment efficacy.
The research is technically sound, timely, and relevant to occupational health, particularly in oncology and pharmacy settings where CSTDs are mandated under USP <800> to minimize exposure to hazardous drugs. It builds on the authors' prior work (e.g., Wilkinson et al., 2018 in PLOS ONE) and addresses gaps in CSTD evaluation by emphasizing semi-volatile surrogates over volatile ones like isopropyl alcohol (IPA). The data support the conclusions, with appropriate controls, replicates (n=4 per task per device), and statistical analysis. However, minor issues in clarity, surrogate justification, and data presentation warrant revisions for improved readability and reproducibility.

Author Response: The author thanks the reviewer for this comment on recommended improvements. The Authors have attempted to address these areas in accordance with all reviewer comments in this round and previous rounds (three lots of duplicate reviewers, making six in total)

However, there are a number of comments and concerns about this manuscript, which are presented below:
1. The introduction need revision. The necessity of conducting the study and its innovation based on the importance of the topic are not clearly presented.

Author Response: The author thanks the reviewer for this comment. The authors have attempted to resolve these concerns. The necessity for conducting the study goes without saying in that pharmacists and importantly nurses need to have scientific evidence to base their selection of choosing a CSTD on to comply with USP<800>. The innovations were reported previously in 2018 in Plos One and in the 2016 Draft NIOSH universal test protocol in using ATD-GC-MS with a semi-volatile material to mimic and replace the use of hazardous drugs in testing for containment of CSTDs.

2. Depending on the type of study, it is necessary to create a section in the introduction titled Literature Review, review and collect the latest studies conducted in this field, and then highlight the necessity of conducting your research.
Here is a suggested structure and key elements to include in the introduction:
Opening Hook: Begin the introduction with a compelling statement, anecdote, or statistic that captures the reader's attention. This hook should generate interest and establish the relevance of the research topic.
Background and Context: Provide a concise overview of the association between your parameters.
Existing Research Gap: Highlight the gap in the current literature, emphasizing that previous research has predominantly focused on the related parameters.
Research Objective and Aim: Clearly state the objective and aim of the research. Explain that the study aims to fill the research gap by providing a holistic framework.
Core Concerns and Research Questions: Introduce the core concerns and research questions that the paper will address.
Significance and Implications: Discuss the significance and potential impact of the research. Highlight the potential benefits for industry practitioners, policymakers, and the broader society.
Overview of the Paper: Provide a brief overview of the paper's structure, indicating how subsequent sections will address each research question and contribute to the overall objective.
Remember to maintain a clear and concise writing style, using appropriate terminology and avoiding excessive jargon. The introduction should set the stage for the research, establishing its importance and creating anticipation for the subsequent sections of the paper.
3. Add a section titled Study Design at the beginning of the Methods section. In the study design section, please provide comprehensive information, including the study location, methods for determining the sample size, sampling method, inclusion and exclusion criteria, type of study, etc.
4. The discussion section is not written according to scientific principles. In the discussion section, a deep comparison with the results of other occupational studies has not been done. In this section, it is necessary to present a more detailed analysis of the results. For this purpose, you can use the following template:
Here are the key factors to address in a strong discussion:
Summary of Findings: Briefly recap the main findings and results of your research. Provide a concise overview of the key outcomes, trends, or patterns that emerged from your study. Ensure that you mention the most important aspects that contribute to the overall objective of your research.
Reiteration of Research Objective: Restate the objective or purpose of your research to remind the reader of the problem you aimed to address or the knowledge gap you aimed to fill. Emphasize how your findings have successfully addressed the research question or objective stated in the introduction.
Significance and Contribution: Highlight the significance and contribution of your research within the broader context of the field. Explain how your study has advanced knowledge, improved methodologies, or provided valuable insights into the topic. Discuss any implications your findings may have for theory, practice, or policy in the field.
Limitations: Acknowledge the limitations of your study. Address more constraints or challenges that may have affected the research process or the interpretation of results. This demonstrates your awareness of the study's boundaries and adds credibility to your work.
Recommendations for Future Work: Suggest areas for further research or specific directions that future studies could take based on the findings and limitations of your research. Highlight potential avenues for expanding or building upon your work to enhance understanding, address unanswered questions, or improve methodologies.
Conclusion Statement: Conclude your paper by summarizing the overall significance and impact of your research. Restate the main takeaway message or key points you want the reader to remember after reading your paper. Leave a lasting impression by reinforcing the importance of your work and its potential implications for the field.
Avoid Introducing New Information: Ensure that your conclusion does not introduce new data, arguments, or information that has not been discussed in the body of your paper. The conclusion should serve as a synthesis and reflection of the research you have already presented.

Author Response: The author thanks the reviewer and really appreciates the reviewers attempts to provide a very detailed analysis of how to write a scientific paper which has clearly taken some time. The authors believe that they have covered the key sections of a robust scientific publication and present some interesting and important scientific data on containment performance of CSTDs using the latest test protocols from the NIOSH. We have attempted to improve the manuscript and taken onboard the comments and recommendations from six reviewers and so with the greatest of respect to the reviewer on this occasion we are keen to keep the changes within what is achievable at this present time. The work represents a significant body of effort and has been in the review process for a few years and the authors would very much like to move forward to share this data with the scientific community and feel that it achieves the standard required for publication.

Other comment:
Surrogate Selection and Justification: While 2-POE is appropriately chosen for its semi-volatility (better simulating drugs like cyclophosphamide), the manuscript could better explain why 2.5% w/v specifically.

Author Response: The author thanks the reviewer for this comment. The authors have addressed this previously (see above). It is multifactorial but 2.5% w/v is a good concentration to represent clinically useful concentration of drug and also produce detectable concentrations in the vapour which is important when using ATD-GC-MS.

Is this the maximum soluble concentration without saturation, or clinically relevant (e.g., mimicking drug doses)? Compatibility testing with CSTD materials (e.g., no degradation of seals/tubing) is mentioned briefly in responses to reviewers but not in the main text—expand this, as solvents like IPA have known incompatibilities (e.g., per Simplivia white papers). Also, discuss potential limitations: 2-POE's aqueous solubility may not fully represent lipophilic drugs.

Author Response: The author thanks the reviewer for this comment. Again, as with the above the authors have sought to explain this in their responses to reviewers above but feel it sits outside of the scope of the main article. These areas have been covered in responses and submission to the federal docket in 2015 when the NIOSH was asking for input on the design of a new test protocol and the author(s) submitted their comments for this.

The key concept is that 2-POE is not attempting to be all things and represent every hazardous drug, it just has an appropriate chemical vapour concentration that provides a safety factor of at least 100 over the most volatile hazardous drug thiotepa. The work done by the UK Health and Safety Executive (Pengelly and Wheeler et al) is very important in helping inform on surrogate selection and their work is cited and provides the only real-world large scale testing of chemical concentrations of potential surrogates including 2-POE and so was borrowed to inform on the parameters for the NIOSH testing. In terms of the chemical compatibility work, the authors have done some work in this area (unpublished) and others including ICU medical (presented at the town hall meeting at the NIOSH in 2016) demonstrating the benefits of using 2-POE over other potential surrogates in terms of compatibility. There is definitely a need for more of this data to be formally published in a peer reviewed article and I would welcome this for one. 70% Isopropyl alcohol is highly inappropriate as a surrogate on many fronts not least its limited chemical compatibility with a lot of medical grade plastic materials. I think we have covered off most points and the 2-POE at 2.5% w/v does fit with some clinically useful concentrations of drugs but the main driver was not to exceed solubility and to achieve a suitable level of detection in the vapour which was achieved (sub one part per billion volume).

Clarity and Redundancy: The introduction and discussion repeat arguments on surrogate volatility (e.g., IPA vs. 2-POE) across sections, making the manuscript lengthy (as noted by Reviewer #1). Consolidate into a dedicated subsection.

Author Response: The author thanks the reviewer for this comment -most of this now redundant and removed.

Transitions between surrogate rationale and methods could be smoother (e.g., "Based on HSE and NIOSH guidelines, we selected 2.5% w/v 2-POE..."). Abstract lacks explicit mention of replicates (n=4) and statistical methods (e.g., ANOVA)—add for completeness.

Author Response: The author thanks the reviewer for this comment. The authors believe they have addressed concerns about additional statistical analysis of the data – see above. Abstract states n=4 and the author have added in to the abstract the work with replication (n=4) which is then stated in all sections of the article. ANOVA not included as the authors do not think that this approach is appropriate or justified.

Data Availability and Reproducibility: Raw CDS data (Chromeleon v7.3) are not manipulable outside the software, as noted, but provide access instructions or deposit processed data (e.g., peak areas, calibration curves) in a repository like Figshare.

Author Response: The author thanks the reviewer for this comment and was happy to investigate Figshare. Having now looked into this it is not compatible with CDS data from Chromeleon which does not surprise me. The author has addressed the comments on access to RAW data files and has provided a robust defence of why commercial grade CDS software was used for compliance purposes and to enable robust processing of the data without the ability to allow the data to be changed (accidentally) by other operators/ third party. As such the processed data made available was the only accessible data from Chromeleon reporting software. There is also a risk when copying RAW data that it becomes changed or compromised in some way which is why it was only handled within the CDS software environment. All processed data has been made available but the authors will keep looking at options as Figshare is not appropriate and cannot handle the CDS format/ extensions on files. Most cannot handle the MS data sizes.

Supplementary materials (e.g., detailed IFU adherence, chamber diagrams) are referenced but not fully described—ensure they include all protocols for replication.

Author Response: The author thanks the reviewer for this comment. All details are included and IFU was followed as per the manufacturer details. Chamber diagrams are contained in 2015 Draft NIOSH documentation and the revision in subsequent publication made by Wilkinson et al (main author).

Statistical Analysis: Appropriate (e.g., 95% CI for blanks; t-tests for comparisons), but clarify handling of Limitations Not Fully Addressed:

Author Response: The author thanks the reviewer for this comment. Not sure what the reviewer is referring to here in terms of limitation. We were conscious that having reduced the article down from 55 to 30 pages not to reintroduce a lot more supplementary material that was not germain or central to the thrust of the article.

o Chamber volume (125 L) is fixed, but real-world compounding occurs in variable environments (e.g., biological safety cabinets)—discuss generalizability.

Author Response: The author thanks the reviewer for this comment but this sits outside of the scope of the article. We are simply testing according to the NIOSH protocols and as such have to stick with the 125L chamber.

o No assessment of liquid spills or surface contamination; focus is vapor only—note this as a scope limitation.

Author Response: The author thanks the reviewer for this comment – the point of the protocol and use of semi-volatile surrogate is that irrespective of the presentation of the release – whether liquid, vapour or aerosol (which is micro-liquid droplets so liquid again) the surrogate will end up in the vapour and get mixed to a final concentration which is measured in the test. What the protocol does not allow for is discrimination between these different types of physical releases which could be interpreted as a limitation but if the releases are below LLOQ then it is not important. If the releases are large enough to be quantified and give large values then it indicates significant releases have taken place and almost certainly will have some liquid component and this is going to be the main concern in terms of route of exposure.

o Task 2 modification (100 mL bag) is justified, but quantify any impact on results (e.g., vs. standard NIOSH setup).

Author Response: The author thanks the reviewer for this comment - the IV bag is simply a receptacle for the displaced liquid and should be self-explanatory. Have added a change of wording to the methods section to explain. Benefits from no bag mean not making the “push” back into the central IV bag and over-pressurising it or having some additional forces on parts of the system that should not occur during clinical practice. We think the addition of the IV bag make the setup more clinically relevant and hence was included.

o Potential bias: Authors collaborated on the 2016 protocol development—disclose if any CSTD manufacturers influenced selection.

Author Response: The author thanks the reviewer for this comment. The authors are very transparent about their contribution to the 2016 Draft NIOSH universal test protocol but a sigificant input was actually from working with the UK Health and Safety Executive and Ian Pengelly who was credited in the 2018 Plos One publication. Anything involving public sector is done in a none bias way – it has to be that way. I cannot speak for the NIOSH but I would like to think adoption of the 2016 Draft NIOSH universal test protocol was based on its merits scientifically and the fact that it borrows fundamentally from published OSHA and existing OH standards used globally for testing exposure risk in work environments.

Minor Technical Issues:
o Units: Consistently use "ppb" (parts per billion) vs. "ppbv" (parts per billion by volume).
o Figures/Tables: Ensure all (e.g., Figure 3 copyright issue resolved) are high-resolution and labeled.

Author Response: The author thanks the reviewer for this comment – done all instances changed to ppbv and LLOQ.

o Language: Generally clear, but some sentences are dense (e.g., abstract methods)—break for readability.

Author Response: The author thanks the reviewer for this comment – done.

Specific Suggestions for Improvement
• Abstract: Add: "Based on four replicates per task/device, with ANOVA for comparisons..." and briefly justify 2-POE ("a semi-volatile surrogate mimicking hazardous drugs"). Author has added in replication and (n=4) but not referred to ANOVA as we still do not feel this statistical analysis is appropriate for the data type.

Author Response: The author thanks the reviewer for this comment – due to limitations on word count and a belief that the abstract is clear in the objectives, methods and results/ conclusions we will keep this on this occasion but thank you. I don’t think it is easy to briefly explain or justify 2-POE selection and certainly not sufficient to include in abstract. We have attempted this in the main body with references to the work of Pengelly and Wheeler et al at HSE.

• Introduction: Consolidate surrogate discussion (pp. 14-15) and add transition sentence as suggested in reviewer responses.

Author Response: The author thanks the reviewer for this comment – quite a few changes done in this section.

• Methods: Expand on 2-POE concentration (e.g., "2.5% w/v represents maximum aqueous solubility and clinical relevance without material incompatibility"). Confirm IFU adherence explicitly.

Author Response: The author thanks the reviewer for this comment – done “The selection of a surrogate 2-POE concentration of 2.5% w/v has been discussed by the authors previously [21] and represents a clinically relevant concentration of hazardous drug whilst maintaining adequate solubility for the surrogate in aqueous solution. It is also a concentration of surrogate that has been demonstrated to have no chemical incompatibility issues with different plastics employed in various medical devices including CSTDs.”

• Results/Discussion: Include a figure plotting release values (even if • Conclusions: Emphasize implications for USP <800> compliance and future NIOSH updates.

Author Response: The author thanks the reviewer for this comment – not sure what is being suggested as we already have this included in figure 2 for the positive liquid releases of 2-POE versus signal in ppbv and so have decided to keep the main body as it is. Hope this is okay?

• General: Shorten by ~10-15% by removing redundancies; aim for <8,000 words.

Author Response: The author thanks the reviewer for this comment – made a significant reduction in word count as per previous reviewers.

Additional Comments
• No ethical concerns (e.g., no human subjects; devices tested in vitro).
• No plagiarism detected; citations are appropriate (e.g., HSE MDHS 104, NIOSH docket).
• This work could influence policy—suggest authors submit to NIOSH for protocol refinement.

Author Response: The author thanks the reviewer for these comments and input – your input is very much appreciated.

Best Regards.

7. PLOS authors have the option to publish the peer review history of their article ([what does this mean?](https://journals.plos.org/plosone/s/editorial-and-peer-review-process#loc-peer-review-history)). If published, this will include your full peer review and any attached files.

If you choose “no”, your identity will remain anonymous but your review may still be made public.

**Do you want your identity to be public for this peer review?** For information about this choice, including consent withdrawal, please see our [Privacy Policy](https://www.plos.org/privacy-policy).

Reviewer #2: No

Reviewer #3: No

Reviewer #4: No

Reviewer #5: No

Reviewer #6: No

Author Response: The author would like to make all comments and reviews public.

[NOTE: If reviewer comments were submitted as an attachment file, they will be attached to this email and accessible via the submission site. Please log into your account, locate the manuscript record, and check for the action link "View Attachments". If this link does not appear, there are no attachment files.]

To ensure your figures meet our technical requirements, please review our figure guidelines: https://journals.plos.org/plosone/s/figures

You may also use PLOS’s free figure tool, NAAS, to help you prepare publication quality figures: https://journals.plos.org/plosone/s/figures#loc-tools-for-figure-preparation.

NAAS will assess whether your figures meet our technical requirements by comparing each figure against our figure specifications.
