# Supplementary material for: Head-to-head containment performance testing of four commercially available closed-system transfer devices using the 2016 Draft NIOSH test protocol and 2.5% w/v 2-phenoxyethanol as challenge agent
Source: PLoS One. 2026 Nov 12;21(11):e0337729. doi: 10.1371/journal.pone.0337729 (PMC13759118; doi:10.1371/journal.pone.0337729)
Supplement: Supplementary file 5 [file pone.0337729.s006.docx]

PONE-D-25-12716R1

Head-to-head containment performance testing of four commercially available closed-system transfer devices using the 2016 Draft NIOSH test protocol and 2.5% w/v 2-phenoxyethanol as challenge agent.

Dear PLOS One

Please find below the authors rebuttal responses to reviewers questions for the above manuscript.

Please find included the following items and files:

- A letter that responds to each point raised by the academic editor and reviewer(s). This letter is a separate file labelled 'Response to Reviewers'.
- A marked-up copy of the latest manuscript that highlights changes made to the original version. This file is labelled 'Revised Manuscript with Track Changes'.
- An unmarked version of the revised paper without tracked changes. This is a separate file labelled 'Manuscript'.

**Authors Responses to Reviewers Questions:**

Reviewer’s Comments – downloaded from portal on Plosone.

**OVERALL FIRST IMPRESSIONS**

**Tone & Audience Fit:** The tone is appropriately academic and aligns well with the intended audience—healthcare professionals, pharmacists, occupational safety experts, and researchers working in oncology and hazardous drug handling. The language is formal but not unnecessarily complex, which is suitable for a journal like *PLOS ONE*.

ASW Author Response: The authors thank the reviewer for this input. The authors have gone to significant lengths now over several reviews to refine the manuscript and appreciate the comments.

**Opening Hook:** The abstract begins effectively by stating the objective clearly: assessing the containment performance of four CSTDs using the 2016 Draft NIOSH protocol and 2-POE as a surrogate. However, the introduction could benefit from a more engaging opening sentence or paragraph that emphasizes the real-world stakes of inadequate CSTD performance (e.g., occupational exposure risks).

ASW Author Response: The authors thank the reviewer for this input. The authors have included the following paragraph to improve the emphasis on inadequate CSTD performance. – see below in Introduction – Background first page.

“A CSTD that does not demonstrate high levels of containment that could result in accidental releases of hazardous drugs may offer no improvement over use of an “open system” of needle and syringe or could lead to higher exposure of healthworkers over that of using an “open system”. This is why the science of CSTD testing and the use of robust validated scientific methods is vital for making informed decisions when purchasing a CSTD technology.”

**ABSTRACT REVIEW**

**Strengths:**

- Clear statement of objectives.
- Concise description of methodology.
- Summary of key findings is well articulated.
- Conclusions are directly tied to the study’s purpose.

ASW Author Response: The authors thank the reviewer for this input.

**Weaknesses:**

- Missing context on why 2.5% w/v 2-phenoxyethanol was selected: This should be briefly explained in the abstract, especially since it's central to the test method.

ASW Author Response: The authors thank the reviewer for this input but the abstract is already at the maximum word count and the authors have found it impossible to provide context in the abstract but this is more than covered in the main manuscript which the authors believe is the correct place for this discussion which is too complex to be covered in just a few words.

- Statistical significance vs. practical relevance: While statistical differences were noted between some CSTD types, the abstract does not clarify what those differences mean in practice (e.g., magnitude of exposure risk).

ASW Author Response: The authors thank the reviewer for this input. Again, the authors believe that the abstract is not the correct place and due to word count constraints cannot support a discussion on this topic. Also, the review appears to misunderstand the key findings – there was no difference observed in performance between the different CSTD tested in the study. In the main discussion the authors do discuss what the impact would be for healthworkers using a poor performance CSTD – higher exposure risk potentially greater than using an “open system” of needle and syringe.

- Lack of mention of sample size or replicates: Readers may want to know how many trials were conducted per device.

ASW Author Response: The authors thank the reviewer for this input. The number of replicates is clearly stated as n=4. This is consistent throughout the abstract and main body of the manuscript.

**Suggested Improvements:**

*“This study evaluated the containment performance of four commercially available closed-system transfer devices (CSTDs) using the 2016 Draft NIOSH universal test protocol and 2.5% w/v 2-phenoxyethanol (2-POE), a semi-volatile surrogate selected for its representative behaviour compared to hazardous drugs.”*

ASW Author Response: The authors thank the reviewer for this suggestion but have not been able to incorporate the suggested wording without exceeding the 500 word count for the abstract.

***Add:*** *“Results are based on [X] replicate tests per device, with statistical comparisons made using [method].”*

ASW Author Response: The authors thank the reviewer for this input. The authors believe that they have included all relevant information for replication. No statistical comparison is made between CSTDs only that they all demonstrated releases that were below LLOQ (not quantifiable).

**INTRODUCTION**

**Flow & Logical Development:**

The introduction builds up to the research question logically, starting with background on hazardous drugs, moving into CSTD classifications, then discussing prior protocols leading to the 2016 NIOSH draft.

**Strengths:**

- Solid foundation on the importance of CSTDs.
- Good explanation of the limitations of earlier testing methods.
- Clear rationale for adopting the 2016 NIOSH protocol.

ASW Author Response: The authors thank the reviewer for this input.

**Areas for Improvement:**

- Repetition of content: Some paragraphs repeat similar points about IPA volatility and surrogate selection across different sections. Consolidate these ideas for better flow.
- Need for clearer transition: Between the discussion of surrogate selection and the actual methodology used in this study. A sentence such as “Given these considerations, we selected 2-POE at a concentration of 2.5% w/v…” would help bridge the gap.

ASW Author Response: The authors thank the reviewer for this input.

**Suggested Edit:**

*“While IPA has been widely used historically, its high volatility makes it unsuitable for accurately simulating the vapor release characteristics of most hazardous drugs. In response, the 2016 Draft NIOSH protocol proposed the use of semi-volatile surrogates, including 2- phenoxyethanol (2-POE). Based on these guidelines, we selected 2.5% w/v 2-POE as the challenge agent for our comparative assessment of four CSTDs.”*

ASW Author Response: The authors thank the reviewer for this input and the above statement has now been incorporated into the introduction section. Thank you.

**METHODS SECTION**

**Overall Assessment:**

The methods section is generally thorough and follows the 2016 Draft NIOSH protocol closely. However, several areas require clarification or expansion to meet the standards expected in a peer-reviewed publication.

**Key Issues:**

**1. Surrogate Selection Justification**

- What led to the choice of 2.5% w/v? Is this concentration within the range recommended by NIOSH or derived empirically?
- Was there any pre-study compatibility testing with the tested CSTD materials? As noted in the literature (e.g., Simplivia white paper), some solvents can degrade tubing or seals.

ASW Author Response: The authors thank the reviewer for this input. The concentration was selected based on the maximum achievable concentration without exceeding the solubility limit. Yes there was pre-study compatibility testing performed both by ICU medical (published in the town hall meeting at NIOSH in 2016) and by the authors (in-house and not published or citable). The below text has now been added to the manuscript in the methods section.

“The selection of 2.5% w/v 2-POE was made so as to maximise the available signal from releases of 2-POE in chamber and hence increase sensitivity in the assay without exceeding the solubility limit for 2-POE.”

“Compatibility between aqueous solutions of 2-POE and the medical devices was assessed prior to test (in-house data) with no observable changes in performance in any of the four CSTDs evaluated in the study following extended contact with the surrogate 2-POE at a concentration of 2.5% w/v.

**2. Device Testing Protocol**

- Were all manipulations performed strictly according to IFUs? If so, specify whether deviations were recorded or monitored.
- Were negative controls included? For example, baseline chamber contamination levels before each test.

ASW Author Response: The authors thank the reviewer for this input. The CSTDs were all operated in strict accordance with the IFUs and no deviations were recorded. The operators conducting the tests used recording sheets to record contemporaneous notes and observations.

Negative controls as described above were conducted by the operators prior to each test. Both the laboratory background was observed on each day of test to record the amount of 2-POE occurring naturally in the background make up air and chamber blanks were performed in the chamber loaded with all of the test equipment prior to test to ensure that there was no 2-POE release prior to test. This is overt in the body of the manuscript and Methods section – see bullet point number 4 below.

“Dual blank chamber measurements were recorded prior to testing of a CSTD to ensure no contamination inside the chamber.”

**3. Analytical Methods**

- Clarify ATD-GC-MS parameters: What were the temperature settings, desorption times, column type, etc.? Without this detail, reproducibility is compromised.

ASW Author Response: The authors thank the reviewer for this input. The authors have now transposed those details from supplementary materials (S1 document) to the Methods section under “Sample analysis”. Specifically the following additional operating parameters and details have been included in the methods section of the manuscript.

“Stainless steel air sampling tubes (thermal desorption [TD tubes) were used with physical dimensions (89 mm (3.5”) long × 6.4 mm (¼”) outer diameter (OD) packed with 200 mg of Tenax TA sorbent, retained between two steel gauzes (Markes, UK). A TD tube conditioner (Markes TC-20, UK) was used to prepare tubes. After conditioning, the tubes were cooled to room temperature and capped with the metal end caps. The sampling tubes were used and immediately prior to analysis spiked with an accurately known mass of *d_8_-*toluene according to a 1mL addition from a 1 parts per million (ppm) standard prepared in four nines di-nitrogen balance gas (BOC, UK). Sampling pumps (Gil-Air, UK) certified to BS EN ISO 13137 and capable of maintaining a constant flow rate of 100 mL/min ± 5% were used. A calibrated flow meter with a measurement uncertainty of ± 2% was used to calibrate the flow rate of the air sampling pump prior to each use. Immediately prior to running a test sample, two chamber blank air samples were collected under conditions identical to the test samples. The blank samples were then used to calculate the experimental limit of detection (LOD) and lower limit of quantitation (LLOQ). A single negative control test was always performed for each closed system transfer device (CSTD) tested for both NIOSH task 1 and task 2. This test was performed following the same procedure as for the CSTD sample tests but using drug vials containing 100% deionized (Milli-Q) water rather than the 2.5% w/v POE challenge agent.

**Determination of breakthrough volume and recovery on TD tubes:**

Breakthrough tests with sample volumes of 3, 6, and 12 liters were performed to verify that no breakthrough and >99.9% recovery from the Tenax TA occurred. Eight test tubes were pre-spiked with internal standard and 5 µl of standard 1, giving tube loading of ~180 ng of 2-POE and approximately 100 ng of *d_8_-*toluene. Two tubes were analyzed by TD-GC-MS immediately and then re-analyzed for traces of residual 2-POE in the second analysis. In between the first and second analysis, a clean blank tube was analyzed to demonstrate no carryover of 2-POE within the instrument and confirmed the trap desorption efficiency. The remaining six tubes were connected to a backup tube, loaded with internal standard but free of 2-POE. Dry air at 100 mL/min was pumped through the tubes for 30 (tubes 3 and 4), 60 (tubes 5 and 6), or 120 minutes (tubes 7 and 8), giving air sampling volumes of 3, 6, and 12-liters, respectively. At the end of the test, all twelve tubes were analyzed by TD-GC-MS and the results were used to confirm the system performance and demonstrate absence of breakthrough for 2-POE.

**Instrument Settings:**

An automated Thermo Scientific Trace 1300 gas chromatograph fitted with a TD100-xr thermal desorption autosampler (Markes, UK) interfaced to a Thermo Scientific ISQ 7000 AEI mass spectrometer, a VOCOL capillary column (60 m length × 0.25 mm diameter × 1 µm film thickness), and data was acquired and processed using the CDS software supplied by Thermo Scientific (Chromeleon version 7.3). The automated thermal desorption (ATD) conditions were as follows: valve temperature 225°C, desorb temperature 300°C, purge time 1 minute, desorption time 10 minutes, desorption flow rate 70 mL/min, trap temperature (low) −30°C, trap temperature (high) 270°C, outlet split flow 30 mL/min, column flow 1.3 mL/min, transfer line temperature 200°C. GC conditions were as follows: initial oven temperature 100°C (hold for 5 minutes), temperature ramp 1: 5°C/min (to 150°C; hold for 0 minutes), temperature ramp 2: 10°C/min (to 200°C; hold for 10 minutes). Under these conditions the retention times of internal standard *d_8_-*toluene and 2-POE were around 9.79 and 22.97 minutes, respectively. The MS conditions were as follows: multiplier voltage = 350 volts, total ion mass (low) = 25, total ion mass (high) = 200, selected ion masses for detection of *d_8_*-toluene = 95 – 105 m/z, selected ion masses for detection of 2-POE = 77, 94, and 138 m/z.”

- Calibration curves: Were they generated fresh for each batch of samples? How was linearity confirmed?

ASW Author Response: The authors thank the reviewer for this input. The instrument was calibrated against a full set of standards for 2-POE on each day of test and the calibration curve data used to interpret the amounts of 2-POE found on tube for each day of test. Linear Regression analysis was performed inside the Chromeleon ver7.3 CDS software and produced a line of best fit with appropriate R2 value >0.99.

**4. Sample Size and Replication**

- How many replicates were run per device? This is essential for interpreting statistical confidence in results.
- Were outliers excluded? If yes, under what criteria?

ASW Author Response: The authors thank the reviewer for this input. The authors have now added the following text in the methods section.

“Each CSTD device test replicate was performed using duplicate TD tubes in each test and the test was performed with replication (n=4) providing in total 8 data points for each NIOSH task. No data points were assessed as being outliers or excluded in the analysis.”

**5. Statistical Methods**

- Which statistical tests were used? T-tests, ANOVA, non-parametric equivalents?
- Was multiple comparison correction applied? Given the number of pairwise comparisons, this should be addressed.
- Define what constitutes a statistically significant difference (e.g., p < 0.05).

ASW Author Response: The authors thank the reviewer for this input. The authors have on previous rounds of review for this manuscript rebutted this same question regarding the application of sophisticated statistical treatment to the data. No tests were deemed appropriate given that the data was below LLOQ and so not quantifiable. The only statistical methodology used was the linear regression analysis performed within Chromeleon CDS software for analysis of the peak area data and conversion in to an amount of 2-POE in nanograms. The statement below has now been added in the methods section of the manuscript.

“The only statistical methodology used was the linear regression analysis performed within Chromeleon v7.3 CDS software for analysis of the peak area data and conversion in to an amount of 2-POE in nanograms. As all data obtained for the CSTDS was below LLOQ no other statistical data treatments were deemed appropriate.”

- Was multiple comparison correction applied? Given the number of pairwise comparisons, this should be addressed.
- Define what constitutes a statistically significant difference (e.g., p < 0.05).

As stated above no pairwise comparisons were made as all data was below LLOQ. These tests would be meaningless. The author has reviewed the manuscript and removed the word “statistical” from any description of comparisons in the data to avoid further confusion.

**Suggested Additions:**

*“All testing procedures were performed strictly in accordance with manufacturer instructions for use (IFU) to ensure validity. Deviations, if any, were documented and reviewed for potential impact on containment integrity.”*

ASW Author Response: The authors thank the reviewer for this input and this statement has been added to the methods section. Thank you.

*“To assess repeatability, five replicate tests were conducted for each CSTD model. Outliers were identified using the Grubbs’ test and removed only when statistically justified.”*

ASW Author Response: The authors thank the reviewer for this input. No action necessary. No data was removed,

*“Chromatographic separation was achieved using a DB-5MS capillary column (30 m × 0.25 mm × 0.25 μm; Agilent Technologies) with helium as the carrier gas at a constant flow rate of 1.2 mL/min. Calibration was verified daily using freshly prepared standards.”*

ASW Author Response: The authors thank the reviewer for this input. The additional detail has now been added in full.

**RESULTS SECTION**

**Strengths:**

- Clear presentation of data.
- Tables summarize findings effectively.
- Statistical outcomes are reported where applicable.

ASW Author Response: The authors thank the reviewer for this input.

**Areas for Clarification:**

- Figure/Table Correspondence: Ensure every figure and table is explicitly referenced in the text and that their captions are self-explanatory.

ASW Author Response: The authors thank the reviewer for this input. This has been addressed previously by the authors and checked again for accuracy.

- Units and precision: Make sure units (e.g., ppbv) are consistent throughout and that decimal places reflect measurement precision.

ASW Author Response: The authors thank the reviewer for this input. This has been addressed previously by the authors and checked again for accuracy.

**Example:**

*“As shown in Table 2, ChemoLock™ barrier demonstrated significantly lower vapour leakage than Tevadaptor™ air-cleaning (p = 0.008).”*

ASW Author Response: The authors thank the reviewer for this input. I am not sure what this refers to as we never applied the p-test to our data, and we were unable to make such discrimination based on our data. I am concerned that the reviewer is referring to something that does not exist in our manuscript – or am I missing the point here. If the point is measurement precision, then I can assure the reviewer that we have addressed measurement precision in a very early edit of the manuscript and have again checked this detail. No action needed.

**DISCUSSION**

**Strengths:**

- Well-grounded in previous literature.
- Interpretation of results aligns with known properties of CSTD technologies.
- Acknowledges limitations of the current methodology.

ASW Author Response: The authors thank the reviewer for this input.

**Weaknesses:**

- Overemphasis on protocol validation: While important, the discussion should center more on the implications of the observed differences among the CSTDs.
- Limited interpretation of practical significance: Beyond statistical significance, what do the differences mean for healthcare worker exposure? Are the measured concentrations below regulatory thresholds?

ASW Author Response: The authors thank the reviewer for this input. The implications on the differences between the four CSTD assessed in the study are none because they all performed to an equivalent standard (below LLOQ). The protocol validation is important as with all of the validations and is what distinguishes the present work with other studies that did not apply robust methodology. The authors are trying to engage the reader with why these important analytical concepts need to be adhered to.

There are no regulatory thresholds for healthworker exposure to hazardous drugs and this is unfortunate when it comes to trying to persuade institutions to use better protection equipment such as CSTDs within their organisations. The authors have revisited this and attempted to highlight the potential dangers from the high positive control releases of 2-POE and what they might mean for hazardous drug releases in clinical practice and how this is avoided by adoption of any one of the four CSTDs assessed in the NIOSH tasks.

The following text has been added to the discussion section of the manuscript.

“It is not possible to draw any correlation between the results obtained using 2-POE as surrogate and healthcare worker exposure to hazardous drugs when handled using the four CSTD evaluated in this study. However, all four CSTDs contained the 2-POE which is a semi-volatile material and thus behaves in an analogous chemical manner to many hazardous drug materials to below LLOQ values. In contrast, when an “open-system” such as needle and syringe was used for positive control test performed significant vapour concentrations of 2-POE were detected at 3.13 ppbv and 6.59 ppbv respectively for release volumes of 5 and 10 microlitres of liquid surrogate solution. Whenever there are liquid releases of hazardous drugs during preparation or administration there is an increased likelihood of healthcare worker exposure by direct or secondary contact with the liquid. None of the four CSTDs assessed in the study produced observable liquid releases and the signal for 2-POE was significantly below those values associated with positive liquid releases of 2-POE and were below quantifiable limits (<LLOQ).”

**Suggested Revisions:**

*“The observed differences in vapour containment among the tested CSTDs suggest that not all systems offer equivalent protection against occupational exposure. These findings support the need for standardised, performance-based evaluation methods such as the 2016 Draft NIOSH protocol.”*

*“Further research is needed to correlate laboratory-measured vapour leakage with real-world exposure scenarios to determine whether these differences translate into clinically meaningful reductions in risk.”*

The following statement has been added to the end of the discussion section – the authors wish to thank the review for this helpful suggestion.

“Further research is needed to correlate laboratory-measured vapour leakage with real-world exposure scenarios to determine whether these differences translate into clinically meaningful reductions in risk.”

**CONCLUSION**

**Strengths:**

- Directly addresses the research objective.
- Succinct and clear.

ASW Author Response: The authors thank the reviewer for this input.

**Suggestion:**

Consider rephrasing the conclusion to emphasise the broader implication:

*“These findings underscore the importance of adopting standardised, semi-volatile surrogates and quantitative testing methodologies to ensure reliable and comparable assessment of CSTD performance in protecting healthcare workers.”*

ASW Author Response: The authors thank the reviewer for this input and have incorporated the suggested reviewers form of words in the conclusions. Thank you once again for this helpful suggestion.

**FIGURES AND TABLES**

**General Observations:**

- All tables appear clear and relevant.
- Figures (if present) must be checked for resolution and labelling consistency.

ASW Author Response: The authors thank the reviewer for this input.

**To Do:**

- Confirm that all figures are labelled with axis titles, units, and legends.

ASW Author Response: The authors thank the reviewer for this input. This has been done.

- Ensure that statistical annotations (e.g., asterisks for p-values) are consistent and explained in the caption.

ASW Author Response: The authors thank the reviewer for this input. No statistical treatments were used and so No Action here.

**LIMITATIONS SECTION**

A dedicated limitations section is missing. Include one after the discussion to address:

- Potential influence of surrogate concentration on device behaviour.
- Absence of real-world validation.
- Limited scope of CSTD models tested.
- Possible variability in operator technique despite adherence to IFUs.

ASW Author Response: The authors thank the reviewer for this input. This has been added thank you and is presented below for clarity.

**“LIMITATIONS SECTION**

The study presented has a number of potential limitations which should be recognized and discussed:

1. The concentration of surrogate 2-POE could have an influence on the performance of the CSTD tested through lack of chemical compatibility. The concentration of 2.5% w/v was chosen so as to both optimize the signal response in the analytical method and reduce the potential for deleterious changes in the CSTD due to exposure to 2-POE. 2-POE 2.5% w/v is also close to the maximum solubility for 2-POE in water and so cannot be increased further. The authors performed functional testing on the four CSTD assessed in the study to satisfy themselves that the use of 2.5% w/v 2-POE as surrogate did not impact negatively on the performance of the CSTD.
2. The study presented was performed deliberately in a laboratory environment where background concentrations of 2-POE were controlled and mitigated. This was important because of the impact that high background levels of 2-POE would have on the experimental LOD and LLOQ. Real-world testing and validation could offer greater assurances that the results obtained are applicable to healthcare worker occupational environments. The testing conducted by Pengelly et al. [26] and Wheeler et al. [27] using 2-POE was performed at scale to better represent worker environments and conditions and the vapour concentrations for 2-POE obtained from these “real-world” tests were the same as the concentrations of 2-POE obtained from the present laboratory scale study. This does provide greater assurance, but more “real-world” testing is required and ideally testing using actual hazardous drugs although this might present an ethical challenge.
3. Only four types of CSTD were evaluated in the present study. This limits the scope of the present study work but when taken together with previously published works of Wilkinson et al. [21,22] there is an increasing body of containment data for CSTDs evaluated using 2.5% w/v 2-POE as surrogate in combination with the 2016 Draft NIOSH universal test protocol. Provided more CSTD can be evaluated using the same scientifically robust quantitative testing methodology this will assist in the selection of suitable CSTD technology within healthcare.
4. Variability in operator during NIOSH testing. It is feasible that different operators whilst adhering to the manufacturer’s instructions for use (IFU) could produce different outcomes from the NIOSH tests. In the present study this was accounted for by employing the same operators for all testing and ensuring that all steps were performed with the same timings between tests and all details were recorded contemporaneously using recording sheets.
5. Four replicates were performed for each of the two NIOSH tasks 1 and 2. This was done to comply with the current 2016 Draft NIOSH universal test protocol as published. However, it is accepted that a greater number of replicates may provide more robust data at an increase in cost. It would therefore be beneficial for a single site to perform a larger cohort of testing of different CSTDs with increased replication and for the results to be published for comparison with smaller scale testing to see if any differences are observed in outcomes.
6. Lack of correlation between releases of 2-POE as surrogate and actual hazardous drug exposure risk. This remains an important limitation which is accepted by the author. What is important is that CSTD should be able to demonstrate containment that is better than that of an “open” system of needle and syringe and this may not be the case. In the present study positive control releases of the surrogate 2-POE were performed at two volume sizes and each produced quantifiable signals for 2-POE with parts per billion volume concentrations of 2-POE in the chamber. Liquid releases of hazardous drug present the greatest exposure risk to healthcare workers based on the amount of hazardous drug that can be contained in liquid compared with vapour. More work needs to be performed however on correlation between healthworker exposure to liquid releases of hazardous drugs in a controlled quantifiable environment and health risks. The study presented does not attempt to make any correlation but rather focuses on the physical containment aspects of four chosen commercial CSTDs in terms of liquid and vapour containment and attempts to compare containment performance across the different CSTD technologies assessed.”

**FINAL COMMENTS**

While the manuscript presents scientifically relevant data and follows a rigorous testing protocol, it currently falls short of PLOS ONE’s standards for clarity, methodological transparency, and ethical disclosure. The following critical issues must be addressed before publication is warranted:

**Major issues preventing recommendation for publication**

**1. Incomplete or Inconsistent Methods Section**

- Lack of reproducibility details: Key aspects such as GC-MS parameters (e.g., column type, temperature ramping), surrogate preparation, and calibration procedures are either missing or underdeveloped.

ASW Responses: This detail has now been added.

- Insufficient statistical reporting: It is unclear which tests were used to compare device performance (e.g., ANOVA, t-test), whether multiple comparisons corrections were applied, or how outliers were handled.

ASW Responses: This has now been clarified – no statistical techniques were applied other than the analytical regression analysis from calibration curves and this has been made more overt and references to statistical differences removed from the text.

Recommendation: Expand the Methods section with full technical specifications and statistical methodology. Ensure that another lab could replicate your work using only this description.

ASW Responses: This detail has now been addressed.

**2. Missing or Inadequate Limitations Section**

- The study lacks a dedicated limitations section, which is essential for contextualising findings and acknowledging constraints.
  No discussion is provided on: Whether the selected surrogate concentration (2.5% w/v 2-POE) affects CSTD integrity or behavior differently than actual hazardous drugs.
  Whether operator variability was controlled or monitored.
  The generalizability of findings beyond the four tested CSTDs.

ASW Responses: This detail has now been added.

Recommendation: Add a standalone Limitations section addressing these points and others related to internal and external validity.

**3. Inconsistent Data Reporting and Figure/Table Integration**

- Some figures and tables are referenced incorrectly or not at all in the main text.
- Units and formatting (e.g., ppbv, μL) are inconsistent across sections.
- There is no clear statement confirming whether raw data will be made publicly available, despite PLOS ONE’s strict open-data policy.

ASW Responses: This detail has now been added and concerns addressed including full disclosure of all data using Figshare as recommended.

**Citation:**

Wilkinson, Alan; Walker, Kate; Ozolina, Laima; Bon, Romana; Wallace, Andrew; Allwood, Michael Charles (2026). This is the data repository of Chromeleon CM7.3 chromatographic data for publication by Wilkinson et al. in Plosone journal.. figshare. Dataset. <https://doi.org/10.6084/m9.figshare.33890500.v1>

Recommendation: Conduct a comprehensive review of all figures and tables to ensure:

- Every one is explicitly cited in the text.
- Captions are self-explanatory.
- Units are consistent and SI-compliant.
- A Data Availability Statement is clearly included, indicating where data can be accessed.

ASW Responses: This detail has now been addressed.

**4. Ethical and Funding Disclosures Require Clarification**

- Although the authors state that “no competing interests exist,” they also disclose funding from ICU Medical, which provided materials and devices used in the study. This creates a potential conflict of interest that needs explicit acknowledgment.

ASW Responses: This detail has now been added

- There is ambiguity around the role of the funder: While the authors claim the funders had no influence, this should be stated more transparently.

ASW Responses: This detail has now been addressed.

Recommendation: Revise the Competing Interests and Funding Disclosure sections to fully align with PLOS ONE’s requirements. Consider including the following language:

“The funders had no role in study design, data collection and analysis, decision to publish, or preparation of the manuscript.”

ASW Responses: This detail has now been addressed.

**5. Abstract and Conclusions Need Tightening**

- The abstract lacks key information about sample size, statistical methods, and practical implications.
- The conclusion emphasizes protocol validation over real-world relevance.

ASW Responses: This detail has now been addressed.

Recommendation:

- Clarify what the differences between CSTD types mean for occupational safety.
- Emphasize how this study fills a gap in published evidence.
- Reframe conclusions to guide healthcare institutions in interpreting containment performance.

ASW Responses: This detail has now been addressed.

**Strengths that support eventual acceptance**

Despite these issues, the manuscript has several strengths that make it worth revising for eventual publication:

- Scientific merit: It addresses an important gap by comparing air-cleaning and barrier CSTDs using a semi-volatile surrogate under a standardised protocol.
- Rigorous methodology: Adherence to the 2016 Draft NIOSH protocol is commendable.
- Potential impact: Findings may inform purchasing decisions and regulatory guidance if interpreted carefully
