# Supplementary material for: Head-to-head containment performance testing of four commercially available closed-system transfer devices using the 2016 Draft NIOSH test protocol and 2.5% w/v 2-phenoxyethanol as challenge agent
Source: PLoS One. 2026 Nov 12;21(11):e0337729. doi: 10.1371/journal.pone.0337729 (PMC13759118; doi:10.1371/journal.pone.0337729)
Supplement: Peer review history

# Peer review: Decision Letter 0

**DOI:** 10.1371/journal.pone.0337729.r001

**Authors:** Antonio Peña-Fernández

26 Jun 2025

Dear Dr. Wilkinson,

Thank you for submitting your manuscript to PLOS ONE. After careful consideration, we feel that it has merit but does not fully meet PLOS ONE’s publication criteria as it currently stands. Therefore, we invite you to submit a revised version of the manuscript that addresses the points raised during the review process.

==============================

Your manuscript has now been peer reviewed. Both reviewers have provided detailed and constructive feedback. I concur with their assessment that addressing these comments will significantly strengthen your manuscript. We would be pleased to consider a revised version once you have incorporated their suggestions.

==============================

Please submit your revised manuscript by Aug 09 2025 11:59PM. If you will need more time than this to complete your revisions, please reply to this message or contact the journal office at plosone@plos.org. When you're ready to submit your revision, log on to https://www.editorialmanager.com/pone/ and select the 'Submissions Needing Revision' folder to locate your manuscript file.

- A rebuttal letter that responds to each point raised by the academic editor and reviewer(s). You should upload this letter as a separate file labeled 'Response to Reviewers'.
- A marked-up copy of your manuscript that highlights changes made to the original version. You should upload this as a separate file labeled 'Revised Manuscript with Track Changes'.
- An unmarked version of your revised paper without tracked changes. You should upload this as a separate file labeled 'Manuscript'.

If you would like to make changes to your financial disclosure, please include your updated statement in your cover letter. Guidelines for resubmitting your figure files are available below the reviewer comments at the end of this letter.

If applicable, we recommend that you deposit your laboratory protocols in protocols.io to enhance the reproducibility of your results. Protocols.io assigns your protocol its own identifier (DOI) so that it can be cited independently in the future. For instructions see: https://journals.plos.org/plosone/s/submission-guidelines#loc-laboratory-protocols. Additionally, PLOS ONE offers an option for publishing peer-reviewed Lab Protocol articles, which describe protocols hosted on protocols.io. Read more information on sharing protocols at https://plos.org/protocols?utm_medium=editorial-email&utm_source=authorletters&utm_campaign=protocols.

We look forward to receiving your revised manuscript.

Kind regards,

Antonio Peña-Fernández, PhD

Academic Editor

PLOS ONE

Journal requirements:

When submitting your revision, we need you to address these additional requirements.

1. Please ensure that your manuscript meets PLOS ONE's style requirements, including those for file naming. The PLOS ONE style templates can be found at

https://journals.plos.org/plosone/s/file?id=wjVg/PLOSOne_formatting_sample_main_body.pdf and

https://journals.plos.org/plosone/s/file?id=ba62/PLOSOne_formatting_sample_title_authors_affiliations.pdf

Additional Editor Comments:

Dear authors,

Your manuscript has now been peer reviewed. Both reviewers have provided detailed and constructive feedback. I concur with their assessment that addressing these comments will significantly strengthen your manuscript. Could you address their feedback and submit a revised version of your manuscript for our consideration, please?

Thank you,

Antonio

[Note: HTML markup is below. Please do not edit.]

Reviewers' comments:

Reviewer's Responses to Questions

**Comments to the Author**

1. Is the manuscript technically sound, and do the data support the conclusions?

Reviewer #1: Yes

Reviewer #2: Partly

**********

2. Has the statistical analysis been performed appropriately and rigorously?

Reviewer #1: Yes

Reviewer #2: Yes

**********

3. Have the authors made all data underlying the findings in their manuscript fully available?

The PLOS Data policy

Reviewer #1: Yes

Reviewer #2: No

**********

4. Is the manuscript presented in an intelligible fashion and written in standard English?

Reviewer #1: Yes

Reviewer #2: Yes

**********

Reviewer #1: The study is thoroughly done

Design is appropriate

Statistics suitable

Conclusions are in accordance with data and acceptable

No ethical problems or plagiarism seen

It is a long paper and could be shortened for faster readability

Reviewer #2: OVERALL FIRST IMPRESSIONS

Tone & Audience Fit:

The tone is appropriately academic and aligns well with the intended audience—healthcare professionals, pharmacists, occupational safety experts, and researchers working in oncology and hazardous drug handling. The language is formal but not unnecessarily complex, which is suitable for a journal like PLOS ONE.

Opening Hook:

The abstract begins effectively by stating the objective clearly: assessing the containment performance of four CSTDs using the 2016 Draft NIOSH protocol and 2-POE as a surrogate. However, the introduction could benefit from a more engaging opening sentence or paragraph that emphasizes the real-world stakes of inadequate CSTD performance (e.g., occupational exposure risks).

ABSTRACT REVIEW

Strengths:

• Clear statement of objectives.

• Concise description of methodology.

• Summary of key findings is well articulated.

• Conclusions are directly tied to the study’s purpose.

Weaknesses:

• Missing context on why 2.5% w/v 2-phenoxyethanol was selected: This should be briefly explained in the abstract, especially since it's central to the test method.

• Statistical significance vs. practical relevance: While statistical differences were noted between some CSTD types, the abstract does not clarify what those differences mean in practice (e.g., magnitude of exposure risk).

• Lack of mention of sample size or replicates: Readers may want to know how many trials were conducted per device.

Suggested Improvements:

“This study evaluated the containment performance of four commercially available closed-system transfer devices (CSTDs) using the 2016 Draft NIOSH universal test protocol and 2.5% w/v 2-phenoxyethanol (2-POE), a semi-volatile surrogate selected for its representative behaviour compared to hazardous drugs.”

Add:

“Results are based on [X] replicate tests per device, with statistical comparisons made using [method].”

INTRODUCTION

Flow & Logical Development:

The introduction builds up to the research question logically, starting with background on hazardous drugs, moving into CSTD classifications, then discussing prior protocols leading to the 2016 NIOSH draft.

Strengths:

• Solid foundation on the importance of CSTDs.

• Good explanation of the limitations of earlier testing methods.

• Clear rationale for adopting the 2016 NIOSH protocol.

Areas for Improvement:

• Repetition of content: Some paragraphs repeat similar points about IPA volatility and surrogate selection across different sections. Consolidate these ideas for better flow.

• Need for clearer transition: Between the discussion of surrogate selection and the actual methodology used in this study. A sentence such as “Given these considerations, we selected 2-POE at a concentration of 2.5% w/v…” would help bridge the gap.

Suggested Edit:

“While IPA has been widely used historically, its high volatility makes it unsuitable for accurately simulating the vapor release characteristics of most hazardous drugs. In response, the 2016 Draft NIOSH protocol proposed the use of semi-volatile surrogates, including 2-phenoxyethanol (2-POE). Based on these guidelines, we selected 2.5% w/v 2-POE as the challenge agent for our comparative assessment of four CSTDs.”

METHODS SECTION

Overall Assessment:

The methods section is generally thorough and follows the 2016 Draft NIOSH protocol closely. However, several areas require clarification or expansion to meet the standards expected in a peer-reviewed publication.

Key Issues:

1. Surrogate Selection Justification

• What led to the choice of 2.5% w/v? Is this concentration within the range recommended by NIOSH or derived empirically?

• Was there any pre-study compatibility testing with the tested CSTD materials? As noted in the literature (e.g., Simplivia white paper), some solvents can degrade tubing or seals.

2. Device Testing Protocol

• Were all manipulations performed strictly according to IFUs? If so, specify whether deviations were recorded or monitored.

• Were negative controls included? For example, baseline chamber contamination levels before each test.

3. Analytical Methods

• Clarify ATD-GC-MS parameters: What were the temperature settings, desorption times, column type, etc.? Without this detail, reproducibility is compromised.

• Calibration curves: Were they generated fresh for each batch of samples? How was linearity confirmed?

4. Sample Size and Replication

• How many replicates were run per device? This is essential for interpreting statistical confidence in results.

• Were outliers excluded? If yes, under what criteria?

5. Statistical Methods

• Which statistical tests were used? T-tests, ANOVA, non-parametric equivalents?

• Was multiple comparison correction applied? Given the number of pairwise comparisons, this should be addressed.

• Define what constitutes a statistically significant difference (e.g., p < 0.05).

Suggested Additions:

“All testing procedures were performed strictly in accordance with manufacturer instructions for use (IFU) to ensure validity. Deviations, if any, were documented and reviewed for potential impact on containment integrity.”

“To assess repeatability, five replicate tests were conducted for each CSTD model. Outliers were identified using the Grubbs’ test and removed only when statistically justified.”

“Chromatographic separation was achieved using a DB-5MS capillary column (30 m × 0.25 mm × 0.25 μm; Agilent Technologies) with helium as the carrier gas at a constant flow rate of 1.2 mL/min. Calibration was verified daily using freshly prepared standards.”

RESULTS SECTION

Strengths:

• Clear presentation of data.

• Tables summarize findings effectively.

• Statistical outcomes are reported where applicable.

Areas for Clarification:

• Figure/Table Correspondence: Ensure every figure and table is explicitly referenced in the text and that their captions are self-explanatory.

• Units and precision: Make sure units (e.g., ppbv) are consistent throughout and that decimal places reflect measurement precision.

Example:

“As shown in Table 2, ChemoLock™ barrier demonstrated significantly lower vapour leakage than Tevadaptor™ air-cleaning (p = 0.008).”

________________________________________

DISCUSSION

Strengths:

• Well-grounded in previous literature.

• Interpretation of results aligns with known properties of CSTD technologies.

• Acknowledges limitations of the current methodology.

Weaknesses:

• Overemphasis on protocol validation: While important, the discussion should center more on the implications of the observed differences among the CSTDs.

• Limited interpretation of practical significance: Beyond statistical significance, what do the differences mean for healthcare worker exposure? Are the measured concentrations below regulatory thresholds?

Suggested Revisions:

“The observed differences in vapour containment among the tested CSTDs suggest that not all systems offer equivalent protection against occupational exposure. These findings support the need for standardised, performance-based evaluation methods such as the 2016 Draft NIOSH protocol.”

“Further research is needed to correlate laboratory-measured vapour leakage with real-world exposure scenarios to determine whether these differences translate into clinically meaningful reductions in risk.”

CONCLUSION

Strengths:

• Directly addresses the research objective.

• Succinct and clear.

Suggestion:

Consider rephrasing the conclusion to emphasise the broader implication:

“These findings underscore the importance of adopting standardised, semi-volatile surrogates and quantitative testing methodologies to ensure reliable and comparable assessment of CSTD performance in protecting healthcare workers.”

FIGURES AND TABLES

General Observations:

• All tables appear clear and relevant.

• Figures (if present) must be checked for resolution and labelling consistency.

To Do:

• Confirm that all figures are labelled with axis titles, units, and legends.

• Ensure that statistical annotations (e.g., asterisks for p-values) are consistent and explained in the caption.

LIMITATIONS SECTION

A dedicated limitations section is missing. Include one after the discussion to address:

• Potential influence of surrogate concentration on device behaviour.

• Absence of real-world validation.

• Limited scope of CSTD models tested.

• Possible variability in operator technique despite adherence to IFUs.

**********

what does this mean?). If published, this will include your full peer review and any attached files.

If you choose “no”, your identity will remain anonymous but your review may still be made public.

**Do you want your identity to be public for this peer review?**  For information about this choice, including consent withdrawal, please see our Privacy Policy

Reviewer #1: No

Reviewer #2: No

**********

[NOTE: If reviewer comments were submitted as an attachment file, they will be attached to this email and accessible via the submission site. Please log into your account, locate the manuscript record, and check for the action link "View Attachments". If this link does not appear, there are no attachment files.]

While revising your submission, please upload your figure files to the Preflight Analysis and Conversion Engine (PACE) digital diagnostic tool, https://pacev2.apexcovantage.com/. PACE helps ensure that figures meet PLOS requirements. To use PACE, you must first register as a user. Registration is free. Then, login and navigate to the UPLOAD tab, where you will find detailed instructions on how to use the tool. If you encounter any issues or have any questions when using PACE, please email PLOS at figures@plos.org. Please note that Supporting Information files do not need this step.

Attachment Submitted filename: ReviewerComments.pdf

# Author comment: Author response to Decision Letter 1

**DOI:** 10.1371/journal.pone.0337729.r002

31 Oct 2025

31st October, 2025

Editor-in-Chief

Email: plosone@plos.org

Plos One

Dear Editor,

On behalf of myself and my co-authors, I respectfully request your consideration of the accompanying revised manuscript, entitled “Head-to-head containment performance testing of four commercially available closed-system transfer devices using the 2016 draft NIOSH test protocol and 2.5% w/v 2-phenoxyethanol as challenge agent,” for publication as an original research article in Plos One.

The authors previously published an article in Plos One based on methodology developed by the same authors in collaboration with the UK Health and Safety Executive (Ian Pengelly, HSE) and submitted to CDC NIOSH in 2015 which later became adopted by CDC NIOSH as the 2016 draft NIOSH protocol for assessment of containtment performance of CSTDs.

Wilkinson et al published original research in 2018 on the development, validation and application of the 2016 draft NIOSH test protocol for assessing the containment performance of four CSTDs using the NIOSH surrogate 2.5% w/v 2-phenoxyethanol (2-POE) in MilliQ water as diluent [7].

The manuscript submitted in January 2025 has previously been reviewed by two independent reviewers at Plos One and was accepted for publication following review. However, due to a technical issue with uploading a file to support copyright use of an image (figure 3) the manuscript was then withdrawn by Plos One and the authors were requested to make a re-submission. The attached revised manuscript is the re-submitted manuscript which has now been subjected to further review by two reviewers.

Please see below the responses to the latest reviewers’ questions:

1. Is the manuscript technically sound, and do the data support the conclusions?

The manuscript must describe a technically sound piece of scientific research with data that supports the conclusions. Experiments must have been conducted rigorously, with appropriate controls, replication, and sample sizes. The conclusions must be drawn appropriately based on the data presented.

Reviewer #1: Yes

Reviewer #2: Partly

Author Response: Review #2 Please provide further clarification to support your statement that the research is only partly robust.

The authors methodology is already published in Plos One (2018) and was adopted by the CDC NIOSH in 2016 forming what is now known as the 2016 draft NIOSH protocol for CSTDs. This methodology allows for quantitative measurement of a released challenge agent by employing time weighted average (TWA) collection of the surrogate 2-phenoxyethanol (2-POE) on to a sorbent tube from a closed chamber of fixed volume 125 Litres. The methodology is therefore superior to the predecessor 2015 draft NIOSH protocol proposed by the NIOSH which measured Isopropyl alocohol (IPA) surrogate in a flow-through setup which results in mixing and dilution of any released IPA material from the CSTDs tested in the chamber [5]. The authors methodology adopted by the CDC NIOSH in 2016 is a protocol that was based and therefore aligns with the UK Health and Safety Executive method mdhs104 (see reference 8 below) for assessment of volatile organic compounds using sorbent tubes, solvent desorption or thermal desorption and gas chromatography [8]. Air sampling methods form the basis of many standard analytical methods used by the United Kingdom (UK) Health and Safety Executive (HSE) and the US Occupational Safety and Health Administration (OSHA) for monitoring levels of volatile and semi-volatile materials in the environment and therefore has precedence in the literature and within regulated environments.

I hope that the above references to the methodology satisfy the reviewer and editor that this is scientifically robust and has passed many different levels of internal and external review by experts in the field. The authors developed the protocol in conjunction with the UK leading experts in occupational health and safety at the UK Health and Safety Executive (Ian Pengelly, HSE UK).

________________________________________

2. Has the statistical analysis been performed appropriately and rigorously?

Reviewer #1: Yes

Reviewer #2: Yes

Author Response: There is no response needed to this question.

________________________________________

3. Have the authors made all data underlying the findings in their manuscript fully available?

The PLOS Data policy requires authors to make all data underlying the findings described in their manuscript fully available without restriction, with rare exception (please refer to the Data Availability Statement in the manuscript PDF file). The data should be provided as part of the manuscript or its supporting information, or deposited to a public repository. For example, in addition to summary statistics, the data points behind means, medians and variance measures should be available. If there are restrictions on publicly sharing data—e.g. participant privacy or use of data from a third party—those must be specified.

Reviewer #1: Yes

Reviewer #2: No

Response: Review #2 This is not correct as all data has been made available. The authors used commercially available proprietary CDS software from Thermo Scientific (Chromeleon v7.3) to collect the RAW instrument data which could be made available if requested but is not readable outside of the Chromeleon v7.3 environment. Anyone that is skilled in the art would know that RAW instrument data can not be manipulated outside of the CDS software.

________________________________________

4. Is the manuscript presented in an intelligible fashion and written in standard English?

PLOS ONE does not copyedit accepted manuscripts, so the language in submitted articles must be clear, correct, and unambiguous. Any typographical or grammatical errors should be corrected at revision, so please note any specific errors here.

Reviewer #1: Yes

Reviewer #2: Yes

Author Response: There is no response needed to this question.

________________________________________Author Summary:

The author has satisfied all of the above reviewer requirements and provided a robust rebuttal to the Reviewer #2 question that the manuscript described a technically sound piece of scientific research based on published scientific methodology adopted by internationally recognized government agencies involved in occupational health and safety research – the CDC NIOSH (USA) and the UK HSE.

More detailed rebuttals (where relevant) are provided below

5. Review Comments to the Author

Please use the space provided to explain your answers to the questions above. You may also include additional comments for the author, including concerns about dual publication, research ethics, or publication ethics. (Please upload your review as an attachment if it exceeds 20,000 characters)

Reviewer #1: The study is thoroughly done

Design is appropriate

Statistics suitable

Conclusions are in accordance with data and acceptable

No ethical problems or plagiarism seen

It is a long paper and could be shortened for faster readability

Response: Review #1 The comment regarding the length of the article is a reasonable comment. The authors have made efforts to reduce the length of the article without removing key arguments and important information. The submitted manuscript is of extreme importance in the context of NIOSH testing of CSTD medical devices in providing the only published protocol that can generate quantitative release data and results on CSTD containment. There is still considerable misinformation and misunderstanding within the scientific community on what constitutes an appropriate surrogate to employ in the testing of CSTDs. Many researchers are still employing the 2015 draft NIOSH test protocol despite the CDC NIOSH stating that 70% IPA is not an appropriate surrogate to use. The authors have themselves provided comments to the CDC NIOSH regarding incompatibility between medical devices and 70% IPA (registered on the federal docket at CDC NIOSH). Given the importance of using an appropriate surrogate such as 2-POE to generate meaningful containment data, the authors felt the length of the manuscript was suggested by using it as a forum for wider discussion around surrogate selection and the criteria used to select an appropriate surrogate. Currently there is a paucity of data from researchers applying this latest 2016 draft protocol with most new published works being based on the 2015 draft NIOSH protocol which does not provide robust quantitative data. As such the authors believe that the manuscript length is justified as it makes a significant and valuable contribution to the discussion around appropriate surrogate selection for use in NIOSH testing of CSTDs and advances the understanding of science in this area.

Reviewer #2: OVERALL FIRST IMPRESSIONS

Tone & Audience Fit:

The tone is appropriately academic and aligns well with the intended audience—healthcare professionals, pharmacists, occupational safety experts, and researchers working in oncology and hazardous drug handling. The language is formal but not unnecessarily complex, which is suitable for a journal like PLOS ONE.

Opening Hook:

The abstract begins effectively by stating the objective clearly: assessing the containment performance of four CSTDs using the 2016 Draft NIOSH protocol and 2-POE as a surrogate. However, the introduction could benefit from a more engaging opening sentence or paragraph that emphasizes the real-world stakes of inadequate CSTD performance (e.g., occupational exposure risks).

Response: Review #2 The comment regarding the hook is a valid one, however there are many tens of scientific papers published on CSTDs and the occupational health risks around handing of hazardous drugs that the authors took a conscious decision that this area has already been covered extensively by other authors. Given the length of the manuscript is already considerable (see comment above review #1) the authors did not want to add additional words without significant justification. The authors have included words and language and made the case for the need for CSTD technology to prevent accidental exposure of health workers to hazardous drugs and readers familiar with this area will be aware of those cited articles. The manuscript is already lengthy and so for brevity reasons the authors would like to resist making further changes that could add to the word count but thank the reviewer for their comments.

ABSTRACT REVIEW

Strengths:

• Clear statement of objectives.

• Concise description of methodology.

• Summary of key findings is well articulated.

• Conclusions are directly tied to the study’s purpose.

Weaknesses:

• Missing context on why 2.5% w/v 2-phenoxyethanol was selected: This should be briefly explained in the abstract, especially since it's central to the test method.

Response: Review #2 In connection with the context on why 2.5% w/v 2POE was selected for use in the testing. This is covered elsewhere in the previous publication by the same authors in Plos One (2018) and published in the 2016 draft NIOSH protocol which is also cited by the authors and is an official US published method. As such the authors believe that the reviewer’s comment is not correct. The authors also cite the basis of selection of 2-POE as coming from the UK Health and Safety Executive research and cite this pivotal work which has received little attention to date. The authors make it quite clear why the selected 2-POE as surrogate as “it more closely resembles real hazardous drugs” in terms of volatility and vapour pressure values. This is a key message, and the poor suitability of other highly volatile NIOSH surrogates such as IPA and ethanol is also clearly addressed by the authors. The authors respectfully do not accept this criticism but thank the reviewer for their comment on this important point.

For further corroboration See Page 14

“It was largely on the basis of this work that Pengelly et al of the UK Health and Safety Executive and Wilkinson et al recommended to NIOSH the inclusion of 2-POE as a candidate for NIOSH to consider in its surrogate selection in the 2016 Draft NIOSH universal protocol [28,14].“

See references 28 and 14 in main manuscript document.

• Statistical significance vs. practical relevance: While statistical differences were noted between some CSTD types, the abstract does not clarify what those differences mean in practice (e.g., magnitude of exposure risk).

Response: Review #2 The quantitative data reported shows the level of containment of each CSTD as evaluated or put more simply how “closed” the CSTD technologies are when evaluated using the 2016 draft NIOSH protocol. The authors discuss how this data allows the end user, health worker and researchers to assess the different CSTD devices and rank them in a hierarchical order based on their release values for 2-POE. The acceptance criteria is provided by the authors and each CSTD is assessed as to whether it resulted in above or below measurable levels of released 2-POE. The authors state that it is not possible to correlate releases of 2-POE with an exposure risk from actual releases of hazardous drugs but there is a relationship between CSTDs that release more 2-POE and those that would release more hazardous drug during operation. As such the CSTDs with the lowest 2-POE release values represent the safest technologies to use and it is the objective of the manuscript to provide robust scientific data on the containment of different CSTD devices and the authors believe that goal is achieved in this publication. At this present time many researchers and healthcare workers are using data from the previous 2015 draft NIOSH protocol based on the releases of 70 IPA as surrogate which is not scientifically robust or quantitative data and as such the authors believe that the manuscript submitted provides better quality data for health workers to make an informed decision as to which CSTD technology to use in their institution.

The authors has included the new sentence in the introduction “However, not all CSTDs offer the same level of operator protection and it is therefore necessary to assess the containment of each CSTD technology using a scientific robust methodology.” To satisfy the reviewer requirements and the author would like to thank the reviewer for this input and suggested amendment.

• Lack of mention of sample size or replicates: Readers may want to know how many trials were conducted per device.

Response: Review #2 This is not correct as all data has been made available and clearly shows that all tests were repeated for each CSTD technology tested I replicate where n=4 (four tests for both Test 1 and Test 2).

Suggested Improvements:

“This study evaluated the containment performance of four commercially available closed-system transfer devices (CSTDs) using the 2016 Draft NIOSH universal test protocol and 2.5% w/v 2-phenoxyethanol (2-POE), a semi-volatile surrogate selected for its representative behaviour compared to hazardous drugs.”

Add:

“Results are based on [X] replicate tests per device, with statistical comparisons made using [method].”

Response: Review #2 This change has been made to satisfy the reviewer #2 in another location in the conclusion however the author has overtly stated four replicates were performed in the abstract objectives; the abstract methods section and now added to the conclusion for additional clarity. Also n=4 made more overt in method section of the manuscript. Four replicates are stated clearly in the results section and in Table 4 and in the conclusions. The authors hope that this satisfies the reviewers requirements and thanks them for their suggestion.

INTRODUCTION

Flow & Logical Development:

The introduction builds up to the research question logically, starting with background on hazardous drugs, moving into CSTD classifications, then discussing prior protocols leading to the 2016 NIOSH draft.

Strengths:

• Solid foundation on the importance of CSTDs.

• Good explanation of the limitations of earlier testing methods.

• Clear rationale for adopting the 2016 NIOSH protocol.

Author Response: Review #2 This is valuable feedback thank you. Nothing more to add here.

Areas for Improvement:

• Repetition of content: Some paragraphs repeat similar points about IPA volatility and surrogate selection across different sections. Consolidate these ideas for better flow.

Response: Review #2 The reviewer points out there is repetition of the arguments over surrogate selection specifically around the use of IPA based on volatility. This is required when providing the rationale for using 2-POE as surrogate (see earlier comments above). Volatility of surrogates is an important theme and hence is repeated to ensure a clear rationale for selection of a semi-volatile surrogate for more representative containment data in the 2016 draft NIOSH protocol. The authors felt that it was necessary to reinforce these fundamental arguments based on the physicochemical properties of different surrogates used in both the 2015 draft NIOSH protocol (IPA) and in the 2016 draft NIOSH protocol (2-POE for example). The arguments that underpin surrogate selection need to be made because the use of semi-volatile as opposed to highly volatile surrogates (such as IPA) is a major plank of the progress made when developing the most recent update to the 2016 draft NIOSH test protocol. The authors believe that in a few different locations it was necessary to revisit the issue of IPA volatility because it is a major scientific limitation in the 2015 draft NIOSH protocol which the CDC NIOSH discuss in detail in the updated 2016 draft NIOSH protocol. Unfortunately since 2016 a number of publications have appeared in the literature that continue to employ the high volatility 70% IPA surrogate and this has led to much confusion in the scientific community. The authors have made significant efforts to reinforce the arguments made by the CDC NIOSH in why IPA is not relevant or could lead to erroneous and incorrect outcomes when used in the NIOSH protocol for CSTDs.

• Need for clearer transition: Between the discussion of surrogate selection and the actual methodology used in this study. A sentence such as “Given these considerations, we selected 2-POE at a concentration of 2.5% w/v…” would help bridge the gap.

Suggested Edit:

“While IPA has been widely used historically, its high volatility makes it unsuitable for accurately simulating the vapor release characteristics of most hazardous drugs. In response, the 2016 Draft NIOSH protocol proposed the use of semi-volatile surrogates, including 2-phenoxyethanol (2-POE). Based on these guidelines, we selected 2.5% w/v 2-POE as the challenge agent for our comparative assessment of four CSTDs.”

Author Response: Review #2 This is a reasonable request and the authors accept the suggested change of wording. The Authors have inserted the below paragraph to satisfy the reviewer comment and thank the reviewer for this suggestion.

“IPA has been widely used in recent publications [34-39] its high volatility makes it unsuitable for accurately simulating the vapor release characteristics of most hazardous drugs. In response, the 2016 Draft NIOSH protocol proposed the use of semi-volatile surrogates, including 2-phenoxyethanol (2-POE) [15]. Based on these guidelines and previous work published on the use of 2-POE by the UK Health and Safety Executive (HSE) [21,28], we selected 2.5% w/v 2-POE as the challenge agent for our comparative assessment of four CSTDs.”

METHODS SECTION

Overall Assessment:

The methods section is generally thorough and follows the 2016 Draft NIOSH protocol closely. However, several areas require clarification or expansion to meet the standards expected in a peer-reviewed publication.

Response: Review #2 The methods are taken directly from the cited and published work of CDC NIOSH for the 2016 draft NIOSH test protocol without any major changes. The methods are also cited in the previous publication by the same authors in Plos one (2018) reference [21] in the manuscript. For reasons of brevity the details are in supplementary materials and again there is a reference to this in the methods section for readers who wish to find this additional information.

Key Issues:

1. Surrogate Selection Justification

• What led to the choice of 2.5% w/v? Is this concentration within the range recommended by NIOSH or derived empirically?

Response: Review #2 The comment on surrogate selection is dealt with elsewhere by the authors in thie rebuttal document and is not covered here in detail.

Regarding the concentration of 2-POE selected and whether this is justified – this level of information is not provided by the 2016 draft NIOSH test protocol document. The concentration selected was derived empirically and is the highest concentration of this surrogate that could be achieved in aqueous solution without the solution reaching saturation. The authors have added a form of words to explain and justify the selected concentration for 2-POE. It is also a concentration at which a small volume of liquid release circa ~100 nL can be detected and quantified using the developed analytical methods allowing sufficiently good sensitivity of detection of small liquid releases of 2-POE. The Authors wish to thank the reviewer for their comment here and hope that this amendment satisfies this requirement.

The authors have made the following insertion of text in the methods section of the manuscript:

“The selection of a surrogate 2-POE concentration of 2.5% w/v has been discussed by the authors previously [21] and represents a clinically relevant concentration of hazardous drug whilst maintaining adequate solubility for the surrogate in aqueous solution.”

• Was there any pre-study compatibility testing with the tested CSTD materials? As noted in the literature (e.g., Simplivia white paper), some solvents can degrade tubing or seals.

Response: Review #2 This is an excellent point made by the reviewers and yes the authors have considered chemical compatibility issues for not only 2.5% w/v 2-POE but also a range of other proposed surrogate materials. Notably empirical studies have been performed (not reported by the authors in the present study) and these have found that there is no detectable incompatibility between a solutions containing 2.5% w/v 2-POE in aqueous solution and a variety of different plastics used in the construction of medical devices including those CSTDs tested in the manuscript. This is relevant at the concentration of 2.5% w/v 2-POE as used in the study. This is in contrast with the use of 70% IPA(aq) and 100% Ethanol, both proposed surrogates for NIOSH testing and both of which are contra indicating demonstrating significant chemical incompatibility with medical devices used in the preparation and administration of hazardous drugs. The authors thank the reviewer for this highly relevant and important point and hope that this satisfies the requirements.

Page 23 – changes are as follows in the methos section

“The selection of a surrogate 2-POE concentration of 2.5% w/v has been discussed by the authors previously [21] and represents a clinically relevant concentration of hazardous drug whilst maintaining adequate solubility for the surrogate in aqueous solution. It is also a concentration of surrogate that has been demonstrated to have no chemical incompatibility issues with different plastics employed in various medical devices including CSTDs. In contrast the authors have observed significant chemical incompatibility issues with a range of polymeric materials and medical devices when used in conjunction with the surrogate 70% IPA (unpublished data).”

2. Device Testing Protocol

• Were all manipulations performed strictly according to IFUs? If so, specify whether deviations were recorded or monitored.

Response: Review #2 IFUs were followed for each device with no deviations.

• Were negative controls included? For example, baseline chamber contamination levels before each test.

Response: Review #2 Yes baseline monitoring was performed as well as more general background monitoring to ensure sufficiently low levels of 2-POE had been achieved and were consistently attained before each test. This is stated in the methods.

3. Analytical Methods

• Clarify ATD-GC-MS parameters: What were the temperature settings, desorption times, column type, etc.? Without this detail, reproducibility is compromised.

Response: Review #2 This data is available in the supplementary materials and in the previous publication cited by the authors reference [21].

• Calibration curves: Were they generated fresh for each batch of samples? How was linearity confirmed?

Response: Review #2 Fresh calibration curves were run in full for each batch of samples tested and linearity of response was confirmed using the CDS software Chromeleon v7.3. which is a gold standard within quality control and quality assurance laboratories and is regulated by strict internal software algorithms/ rules.

4. Sample Size and Replication

• How many replicates were run per device? This is essential for interpreting statistical confidence in results.

Response: Review #2 This is stated and there were four replicates tested for each device. Due to the cost and complexity of conducting these type of tests we performed the required number of replicates as required by 2016 draft NIOSH protocol however in an ideal world it would have been nice to have obtained more replicate data for improved confidence. Where the data was always below LOQ there is no need or relevance for applying further statistical treatments to the data.

• Were outliers excluded? If yes, under what criteria?

Response: Review #2 No outliers were found and all data was included.

5. Statistical Methods

• Which statistical tests were used? T-tests, ANOVA, non-parametric equivalents?

Response: Review #2 Linear regression was used to calculate concentrations of 2-POE using algorithms within the CDS software Chromeleon 7.3 software. The line of best fit was applied that had the optimum regression coefficient and when calibrators were injected provided suitable levels of recovery (90-110%). As the data was below LOQ then further statistical treatments of the data was not appropriate or relevant. All data is reported as being below LOQ or more properly below LLOQ.

• Was multiple comparison correction applied? Given the number of pairwise comparisons, this should be addressed.

• Define what constitutes a statistically significant difference (e.g., p < 0.05).

Suggested Additions:

“All testing procedures were performed strictly in accordance with manufacturer instructions for use (IFU) to ensure validity. Deviations, if any, were documented and reviewed for potential impact on containment integrity.”

“To assess repeatability, five replicate tests were conducted for each CSTD model. Outliers were identified using the Grubbs’ test and removed only when statistically justified.”

“Chromatographic separation was achieved using a DB-5MS capillary column (30 m × 0.25 mm × 0.25 μm; Agilent Technologies) with helium as the carrier gas at a constant flow rate of 1.2 mL/min. Calibration was verified daily using freshly prepared standards.”

Author Responses:

As stated above the data always reported below LLOQ data and so further statistical treatment was not appropriate. No pairwise test was performed because the numbers are below LLOQ and so not meaningful.

The authors have added the following bullet point in the methods section:

“All testing procedures were performed strictly in accordance with manufacturer instructions for use (IFU) for each CSTD technology evaluated to ensure validity.”

The authors have referred the reader to the supplementary materials section for full instrument operational details such as temperatures, columns, flow rates etc. This information is supplied in full.

RESULTS SECTION

Strengths:

• Clear presentation of data.

• Tables summarize findings effectively.

• Statistical outcomes are reported where applicable.

Areas for Clarification:

• Figure/Table Correspondence: Ensure every figure and table is explicitly referenced in the text and that their captions are self-explanatory.

Author Response: The figures and tables are explicitly references in the manuscript.

• Units and precision: Make sure units (e.g., ppbv) are consistent throughout and that decimal places reflect measurement precision.

Author Response: The figures are consistent in units and precision reported.

Example:

“As shown in Table 2, ChemoLock™ barrier demonstrated significantly lower vapour leakage than Tevadaptor™ air-cleaning (p = 0.008).”

Author Response: The authors politely do not know where this data is cited from. The main results are presented in table 4. Also the releases are not just vapour but a total release from liquid, aerosol and vapour. All data is presented to 2 decimal places or three significant figures.

DISCUSSION

Strengths:

• Well-grounded in previous literature.

• Interpretation of results aligns with known properties of CSTD technologies.

• Acknowledges limitations of the current methodology.

Weaknesses:

• Overemphasis on protocol validation: While important, the discussion should center more on the implications of the observed differences among the CSTDs.

Author Response: The authors didn’t wish to over interpret the data because all devices demonstrated below LOQ release data. The authors do discuss the implications in terms of even the air-cleaning Chemolock CSTD providing equivalent containment performance to the physical barrier device of ChemoLock showing that the air-cleaning technology is equally effective.

• Limited interpretation of practical significance: Beyond statistical significance, what do the differences mean for healthcare worker exposure? Are the measured concentrations below regulatory thresholds?

Author Response: There are no regulatory thresholds for exposure to hazardous drugs and this is part of the wider problem when it comes to deriving acceptance criteria for CSTD technologies and the authors accept this limitation. However, the authors are using a highly sensitive analytical method and procedures and are able to demonstrate below quantifiable release performance from a range of CSTDs which supports their use when handling hazardous drugs. In the absence of any other containment data this is a step in the right direction. The data offers significant advantage over similar data reported using 70% IPA as surrogate because those data could reflect chemical incompatibility between the surrogate and the item under test. The fundamental requirement of any scientific experiment or test should be that the test conditions or equipment do not influence or effect the outcome of the experiment. In the case of 70% IPA this is not the case but in the submitted manuscript the authors have confidence that the containment data is robust and reliable and does reflect how these CSTD technologies would perform when used to handle hazardous drugs. This is a significant contribution to this area of scientific research.

Suggested Revisions:

“The observed differences in vapour containment among the tested CSTDs suggest that not all systems offer equivalent protection against occupational exposure. These findings support the need for standardised, performance-based evaluation methods such as the 2016 Draft NIOSH protocol.”

“Further research is needed to correlate laboratory-measured vapour leakage with real-world exposure scenarios to determine whether these differences translate into clinically meaningful reductions in risk.”

Author Response: The authors thank the reviewer for the above discussion and helpful suggestion of revision and has taken this onboard and offers the below revision:

“The observed differences in containment of 2-POE among the tested CSTDs suggest that not all CSTD technologies offer equivalent protection against occupational exposure. These findings support the need for standardised, performance-based evaluation methods such as the 2016 Draft NIOSH protocol.

Further research is needed to attempt to correlate laboratory-measured releases of a semi-volatile surrogate such as 2-POE with real-world exposure scenarios to determine whether these differences translate into clinically meaningful reductions in risk. The authors suggest that this type of investigation must be performed in a controlled laboratory environment where background levels of 2-POE can be controlled and monitored. One of the major limitations of hospital pharmacy or ward-based measurements of hazardous drugs from surface wipe studies is the absence of any controls and lack of reliability when attempting to assess the impact of an intervention such as a CSTD when this is against an unknown level of background surface contamination (including cross contamination) from hazardous drugs used in the hospital environment. As such it is theoretically possible to conduct a surface wipe intervention study for employing a CSTD versus no CSTD and obtain data showing that a CSTD increases exposure to hazardous drugs based on the amount of background cross-contamination on surfaces of devices handled in the hospital.”

The authors hope that the revision meets with the satisfaction of the reviewer.

CONCLUSION

Strengths:

• Directly addresses the research objective.

• Succinct and clear.

Suggestion:

Consider rephrasing the conclusion to emphasise the broader implication:

“These findings underscore the importance of adopting standardised, semi-volatile surrogates and quantitative testing methodologies to ensure reliable and comparable assessment of CSTD performance in protecting healthcare workers.”

Author Response: The authors wish to thank the reviewer for their valued contribution and suggested change and have added the below sentence to the final conclusion.

“These findings underscore the importance of adopting standardised, semi-volatile surrogates as provided by the 2016 draft NIOSH protocol such as 2-POE and quantitative testing methodologies to ensure reliable and comparable assessment of CSTD performance in protecting healthcare workers.”

FIGURES AND TABLES

General Observations:

• All tables appear clear and relevant.

• Figures (if present) must be checked for resolution and labelling consistency.

To Do:

• Confirm that all figures are labelled with axis titles, units, and legends.

• Ensure that statistical annotations (e.g., asterisks for p-values) are consistent and explained in the caption.

Author Response: this is done.

LIMITATIONS SECTION

A dedicated limitations section is missing. Include one after the discussion to address:

• Potential influence of surrogate concentration on device behaviour.

Author Response: this is done.

• Absence of real-world validation.

Author Response: this is done.

• Limited scope of CSTD models tested.

Author Response: this is done.

• Possible variability in operator technique despite adherence to IFUs.

Author Response: this is done.

We the authors would like to once again thank the reviewers for their time and attention and confirm that all of the reviewers questions have been addressed in full and believe that the work described is now in a suitable form for consideration for publication as an original research article and this manuscript is approved by all authors for publication, has not been previously published, and is not under consideration for publication by another journal.

Thank you for your kind consideration.

There have been no significant developments in the area which this manuscript addresses since its original submission in 2022 and there is still no update from NIOSH on the finalization of the draft 2016 NIOSH protocol.

However, there is considerable confusion within the pharmacy community because the earlier draft 2015 NIOSH protocol is still available and being used to assess the vapour containment of CSTDs. There is a growing body of evidence that demonstrates a contra-indication between 70% IPA, the surrogate employed in the 2015 draft NIOSH protocol and a wide range of medical devices used in administration of oncolytic drugs which results in failure of those devices.

It is therefore the opinion of the authors that it is increasingly important to provide robust scientific evidence of an alternative test protocol such as the 2016 draft NIOSH protocol in conjunction with valid semi-volatile surrogates such as 2-POE with robust assessment for additional commercially available CSTDs. This is the purpose of the submitted revised manuscript.

Sincerely,

Alan-Shaun Wilkinson Ph.D

Biopharma Stability Testing Laboratory Ltd.

BioCity Nottingham,

Innovation R3,

Pennyfoot Street

Nottingham, NG1 1GF

United Kingdom

Email: alan@biopharmatesting.co.uk

Tel: (+)447841204528

________________________________________

Financial Disclosure Statement:

The author ASW received funding from ICU medical (SA) to support the research study including the supply of the commercially available closed system devices that were tested as part of the study.

"The funders had no role in study design, data collection and analysis, decision to publish, or preparation of the manuscript."

Attachment Submitted filename: Response to Reviewers.docx

# Peer review: Decision Letter 1

**DOI:** 10.1371/journal.pone.0337729.r003

**Authors:** Antonio Peña-Fernández

6 Jan 2026

Dear Dr. Wilkinson,

Thank you for submitting your manuscript to PLOS ONE. After careful consideration, we feel that it has merit but does not fully meet PLOS ONE’s publication criteria as it currently stands. Therefore, we invite you to submit a revised version of the manuscript that addresses the points raised during the review process.

==============================

Your revised manuscript has been evaluated by new reviewers. Based on their reports, substantial revisions are required before the manuscript can be considered for publication in our journal. We kindly ask you to address all of their comments and submit a thoroughly revised version of your manuscript for further consideration.

==============================

Please submit your revised manuscript by Feb 20 2026 11:59PM. If you will need more time than this to complete your revisions, please reply to this message or contact the journal office at plosone@plos.org. When you're ready to submit your revision, log on to https://www.editorialmanager.com/pone/ and select the 'Submissions Needing Revision' folder to locate your manuscript file.

- A letter that responds to each point raised by the academic editor and reviewer(s). You should upload this letter as a separate file labeled 'Response to Reviewers'.
- A marked-up copy of your manuscript that highlights changes made to the original version. You should upload this as a separate file labeled 'Revised Manuscript with Track Changes'.
- An unmarked version of your revised paper without tracked changes. You should upload this as a separate file labeled 'Manuscript'.

If you would like to make changes to your financial disclosure, please include your updated statement in your cover letter. Guidelines for resubmitting your figure files are available below the reviewer comments at the end of this letter.

If applicable, we recommend that you deposit your laboratory protocols in protocols.io to enhance the reproducibility of your results. Protocols.io assigns your protocol its own identifier (DOI) so that it can be cited independently in the future. For instructions see: https://journals.plos.org/plosone/s/submission-guidelines#loc-laboratory-protocols. Additionally, PLOS ONE offers an option for publishing peer-reviewed Lab Protocol articles, which describe protocols hosted on protocols.io. Read more information on sharing protocols at https://plos.org/protocols?utm_medium=editorial-email&utm_source=authorletters&utm_campaign=protocols.

We look forward to receiving your revised manuscript.

Kind regards,

Antonio Peña-Fernández, PhD

Academic Editor

PLOS One

Journal Requirements:

If the reviewer comments include a recommendation to cite specific previously published works, please review and evaluate these publications to determine whether they are relevant and should be cited. There is no requirement to cite these works unless the editor has indicated otherwise.

Additional Editor Comments:

Dear authors,

Your revised manuscript has been evaluated by new reviewers. Based on their reports, substantial revisions are required before the manuscript can be considered for publication in our journal. We kindly ask you to address all of their comments and submit a thoroughly revised version of your manuscript for further consideration.

Thank you,

Antonio

[Note: HTML markup is below. Please do not edit.]

Reviewers' comments:

Reviewer's Responses to Questions

**Comments to the Author**

Reviewer #2: All comments have been addressed

Reviewer #3: (No Response)

Reviewer #4: All comments have been addressed

Reviewer #5: All comments have been addressed

Reviewer #6: All comments have been addressed

**********

2. Is the manuscript technically sound, and do the data support the conclusions?

Reviewer #2: Yes

Reviewer #3: (No Response)

Reviewer #4: Yes

Reviewer #5: Yes

Reviewer #6: Yes

**********

3. Has the statistical analysis been performed appropriately and rigorously?

Reviewer #2: Yes

Reviewer #3: (No Response)

Reviewer #4: Yes

Reviewer #5: Yes

Reviewer #6: No

**********

4. Have the authors made all data underlying the findings in their manuscript fully available?

The PLOS Data policy

Reviewer #2: Yes

Reviewer #3: (No Response)

Reviewer #4: Yes

Reviewer #5: Yes

Reviewer #6: Yes

**********

5. Is the manuscript presented in an intelligible fashion and written in standard English?

Reviewer #2: Yes

Reviewer #3: (No Response)

Reviewer #4: Yes

Reviewer #5: Yes

Reviewer #6: Yes

**********

Reviewer #2: Thank you for addressing the comments. You have thoroughly addressed all prior concerns with clear, thoughtful revisions that significantly strengthen the manuscript. The introduction now provides a more cohesive rationale for the study, and the methods section has been expanded with essential technical details—particularly regarding analytical procedures, replication, and statistical analysis—ensuring reproducibility. The inclusion of a dedicated limitations section adds necessary context to the findings, enhancing transparency and scientific rigor. Clarifications around surrogate selection, data handling, and device testing protocols align well with PLOS ONE’s standards. The discussion appropriately interprets results in light of real-world implications for healthcare worker safety and CSTD performance evaluation. All figures and tables are now accurately referenced and clearly presented, with consistent units and annotations. Most importantly, the authors have maintained a balanced, evidence-based tone throughout, avoiding overstatement while still highlighting the study’s value in enabling direct comparison between air-cleaning and barrier CSTD technologies. The financial disclosure and competing interests statements are now fully transparent, confirming that the funders had no role in study design or data interpretation. These revisions have transformed the manuscript into a robust, well-executed study that makes a valuable contribution to occupational safety in healthcare settings

Reviewer #3: This manuscript presents a head-to-head containment performance comparison of four commercially available Closed System Transfer Devices (CSTDs) using the 2016 Draft NIOSH test protocol and 2.5% w/v 2-phenoxyethanol (2-POE) as a surrogate. The study is methodologically sound, builds upon previously published and NIOSH-adopted protocols, and provides quantitative containment data for both physical barrier and air-cleaning CSTDs. The conclusions are well-supported by the data. However, the manuscript is excessively long and repetitive, particularly in the Introduction and Discussion sections. Several clarifications and improvements in data presentation, statistical reporting, and focus are needed before publication in this journal.

Introduction

The introduction is generally sound but could be more focused. Currently, it provides extensive background on general populations before narrowing to the target group. I suggest tightening the narrative to more directly foreground the study population and research gap. Additionally, please incorporate recent, relevant references to strengthen the theoretical foundation, such as:

DOI: 10.18502/ijph.v52i10.13854

DOI: 10.3923/rjmsci.2016.111.115

DOI: 10.3233/WOR-220077

doi: 10.3233/WOR-203165

https://www.makhillpublications.co/public/files/published-files/mak-rjas/2016/7-547-553.pdf

At the continue 10 major and minor comment. :Major Comments

### **10 MAJOR COMMENTS**

1. **Excessive Length & Repetition**

The manuscript is unnecessarily long (over 120 pages), with significant repetition, especially regarding the rationale for surrogate selection and criticisms of the 2015 NIOSH protocol. The authors should consolidate repetitive sections (e.g., surrogate selection arguments) and move detailed methodological descriptions to Supplementary Materials.

2. **Lack of Clear Research Hypothesis & Objectives**

While the objective is stated in the Abstract, the main text does not clearly articulate a testable hypothesis. The study appears descriptive rather than hypothesis-driven. A clearer framing of the research question would strengthen the paper.

3. **Insufficient Statistical Detail**

The authors state that “no statistically significant difference was found,” but they do not report the specific statistical tests used, p-values, or effect sizes. Since all results were below the LOQ, the appropriateness of statistical comparisons should be justified or clearly stated that formal testing was not performed.

4. **Data Availability Statement is Inadequate**

The authors claim data are available “within the manuscript and Supporting Information,” but the raw instrument data (Chromeleon files) are not accessible. This violates PLOS ONE’s data policy. They should deposit raw data in a public repository or provide processed data in a usable format (e.g., CSV).

5. **Overemphasis on Protocol Validation**

A large portion of the Discussion is devoted to defending the 2016 NIOSH protocol and critiquing other methods. While context is important, the focus should shift toward interpreting the *results* of this study and their implications for CSTD selection and healthcare worker safety.

6. **Limited Discussion of Practical Significance**

The clinical or occupational health implications of the findings are underdeveloped. What does “below LOQ” mean in terms of real-world exposure risk? How should end-users interpret these results when selecting a CSTD?

7. **Sample Size Justification**

The use of n=4 replicates is based on the NIOSH protocol but is not statistically justified. A power calculation or justification for the sample size should be provided, especially given that all results were below LOQ.

8. **Incomplete Reporting of Methodological Details**

Key details (e.g., ATD-GC-MS parameters, column temperatures, desorption times) are relegated to Supplementary Materials but are critical for reproducibility. Essential parameters should be summarized in the main Methods section.

9. **Potential Conflict of Interest**

The study was funded by ICU Medical, the manufacturer of two tested devices (ChemoLock™). Although the authors state the funder had no role in study design or analysis, the potential for bias should be more transparently discussed in the Limitations section.

10. **No Dedicated Limitations Section**

The manuscript lacks a structured Limitations section. Important limitations include the use of a single surrogate concentration, the absence of real-world validation, the small number of CSTD models tested, and the lack of exposure-risk correlation.

---

### **10 MINOR COMMENTS**

1. **Abstract Clarity**

The Abstract should briefly state *why* 2-POE was chosen as the surrogate. Add one sentence on its semi-volatile properties and relevance to hazardous drugs.

2. **Figure & Table Referencing**

Some figures (e.g., Figure 3) are not clearly referenced in the text. All figures and tables should be explicitly cited in the order they appear.

3. **Typos and Inconsistencies**

- Page 75, Conclusions: “NIOSH Task 2each” → “NIOSH Task 2 each.”

- Page 96: “was-based-on It is” → “It is.”

- Page 107: “In feetfact” → “In fact.”

4. **Units Consistency**

Use “ppbv” or “ppb” consistently. In some places, “ppb” is used without specifying volume.

5. **Positive Control Reporting**

Positive controls were performed at 5 and 10 µL, but the text references an ability to quantify down to ~100 nL. Clarify the relationship between the reported LOQ (0.41 ppb) and the minimum quantifiable release volume.

6. **Reference Formatting**

Some references are incomplete (e.g., Ref. 30–33 are “private communication” or presentations). Replace or cite formally where possible.

7. **Repetitive Language in Introduction**

The Introduction repeats points about USP <800> and NIOSH protocols. Streamline to improve flow.

8. **Conclusion Restructuring**

The Conclusion is lengthy and reiterates the Discussion. Condense to a clear summary of findings, implications, and future research.

9. **Definition of LOQ/LLOQ**

The terms LOQ and LLOQ are used interchangeably. Use one term consistently.

10. **Acknowledgments Section**

The funding statement is repeated in multiple places. Consolidate into a single Financial Disclosure section.

---

**Recommendation**

**Major Revision Required.** The manuscript presents valuable and robust containment data for CSTDs using an advanced NIOSH protocol. However, it requires significant condensation, improved statistical reporting, clearer data availability, and a sharper focus on the study’s findings and implications before it is suitable for publication in this journal.

Discussion Depth

The current discussion is somewhat superficial. Please provide a more in-depth interpretation of findings in relation to existing literature, integrating the updated references above.

I look forward to reviewing the revised manuscript. Please address all points comprehensively in your response letter and manuscript track changes.

Please update and ensure accurate, complete citations with DOIs. Incorporate recent literature to strengthen the discussion, such as:

DOI: 10.18502/ijph.v52i10.13854

DOI: 10.3923/rjmsci.2016.111.115

DOI: 10.3233/WOR-220077

doi: 10.3233/WOR-203165

---

Reviewer #4: The article mentions: "No care over of 2-POE was observed in the chamber following testing, and there is no additional cleaning of the chamber was required between tests”

However, there is no clear explanation here on how to verify 'no residue'. Is it through visual inspection, cabin air sampling, or surface wiping? If air sampling is used, is the detection limit low enough to ensure that there are no low-level residual effects on subsequent testing?

Improvement suggestion: Can you briefly explain the specific method for verifying "no residue" (e.g. "verifying by sampling the cabin with standard air and confirming that the 2-POE concentration is below the detection limit after testing") to enhance the credibility of this statement.

Reviewer #5: Comments to the Author

1. Abstract

• The abstract clearly states objectives, methods, and key findings. However, it would benefit from briefly noting why 2.5% w/v 2-POE was selected over other surrogates, reinforcing the methodological rigor.

• Inclusion of “n=4 replicates” is appropriate, but specifying that results were below LOQ for all devices quickly highlights the high containment performance observed.

2. Introduction

• The background effectively establishes the importance of CSTDs and the evolution of NIOSH protocols. However, the section could be tightened to reduce repetition regarding surrogate volatility.

• The rationale for selecting 2-POE over IPA is well-supported by references to HSE and NIOSH documents, strengthening the scientific premise.

3. Methods

• The adherence to the 2016 NIOSH draft protocol and manufacturer IFUs is a strength, ensuring consistency and validity.

• Clarification on the chemical compatibility testing of 2.5% w/v 2-POE with CSTD materials would enhance methodological transparency.

• While replicate number (n=4) is stated, a brief justification for this sample size in relation to statistical power would be useful.

4. Results

• Data presentation in Table 4 is clear, showing all devices performed below LLOQ. The use of confidence intervals adds robustness.

• Positive and negative control results suitably demonstrate system suitability and low background interference.

• The ability to quantify releases down to ~650 nL is a notable technical achievement and should be emphasized as a key outcome.

5. Discussion and Conclusion

• The discussion appropriately interprets the parity between air-cleaning and barrier CSTDs, a significant finding for OHS practice.

• However, the practical implications for healthcare worker exposure risk are underdeveloped; linking LOQ values to potential hazard thresholds would strengthen relevance.

• The conclusion rightly advocates for standardized semi-volatile surrogate use, but could more explicitly call for NIOSH to finalize the 2016 protocol based on this evidence.

6. References

• References are comprehensive and appropriate, covering key regulatory documents and prior research.

• Including a citation for the chemical compatibility claim between 2-POE and medical device plastics would further support the methods.

Final Recommendation

Minor Revisions. The manuscript presents a technically sound, well-controlled study that advances the evaluation of CSTD containment performance. The methodology is robust, data support the conclusions, and the work has direct relevance to occupational health practice. Revisions should focus on refining the discussion of practical implications, clarifying methodological justifications, and minor editorial tightening to enhance clarity.

Reviewer #6: Ref.: PONE-D-25-12716R1

Title:

Head-to-head containment performance testing of four commercially available closed-system transfer devices using the 2016 Draft NIOSH test protocol and 2.5% w/v 2-phenoxyethanol as challenge agent

Dear editor

Greetings

Thank you for giving me the opportunity to review this manuscript. Thanks to the valuable efforts of the authors. This study is a practical study whose findings can lead to proper awareness for the society. This manuscript presents a head-to-head comparison of four closed-system transfer devices (CSTDs)—two physical barrier types (ChemoLock™ barrier and BD PhaSeal™) and two air-cleaning types (ChemoLock™ air-cleaning and Tevadaptor™)—using the 2016 Draft NIOSH universal protocol for assessing containment performance during hazardous drug compounding and administration. The challenge agent is 2.5% w/v 2-phenoxyethanol (2-POE) in water, analyzed via automated thermal desorption gas chromatography-mass spectrometry (ATD-GC-MS). The study concludes that all devices perform below the limit of quantification (LOQ; 0.41 ppb), with no significant differences between barrier and air-cleaning types, suggesting equivalent containment efficacy.

The research is technically sound, timely, and relevant to occupational health, particularly in oncology and pharmacy settings where CSTDs are mandated under USP <800> to minimize exposure to hazardous drugs. It builds on the authors' prior work (e.g., Wilkinson et al., 2018 in PLOS ONE) and addresses gaps in CSTD evaluation by emphasizing semi-volatile surrogates over volatile ones like isopropyl alcohol (IPA). The data support the conclusions, with appropriate controls, replicates (n=4 per task per device), and statistical analysis. However, minor issues in clarity, surrogate justification, and data presentation warrant revisions for improved readability and reproducibility.

However, there are a number of comments and concerns about this manuscript, which are presented below:

1. The introduction need revision. The necessity of conducting the study and its innovation based on the importance of the topic are not clearly presented.

2. Depending on the type of study, it is necessary to create a section in the introduction titled Literature Review, review and collect the latest studies conducted in this field, and then highlight the necessity of conducting your research.

Here is a suggested structure and key elements to include in the introduction:

Opening Hook: Begin the introduction with a compelling statement, anecdote, or statistic that captures the reader's attention. This hook should generate interest and establish the relevance of the research topic.

Background and Context: Provide a concise overview of the association between your parameters.

Existing Research Gap: Highlight the gap in the current literature, emphasizing that previous research has predominantly focused on the related parameters.

Research Objective and Aim: Clearly state the objective and aim of the research. Explain that the study aims to fill the research gap by providing a holistic framework.

Core Concerns and Research Questions: Introduce the core concerns and research questions that the paper will address.

Significance and Implications: Discuss the significance and potential impact of the research. Highlight the potential benefits for industry practitioners, policymakers, and the broader society.

Overview of the Paper: Provide a brief overview of the paper's structure, indicating how subsequent sections will address each research question and contribute to the overall objective.

Remember to maintain a clear and concise writing style, using appropriate terminology and avoiding excessive jargon. The introduction should set the stage for the research, establishing its importance and creating anticipation for the subsequent sections of the paper.

3. Add a section titled Study Design at the beginning of the Methods section. In the study design section, please provide comprehensive information, including the study location, methods for determining the sample size, sampling method, inclusion and exclusion criteria, type of study, etc.

4. The discussion section is not written according to scientific principles. In the discussion section, a deep comparison with the results of other occupational studies has not been done. In this section, it is necessary to present a more detailed analysis of the results. For this purpose, you can use the following template:

Here are the key factors to address in a strong discussion:

Summary of Findings: Briefly recap the main findings and results of your research. Provide a concise overview of the key outcomes, trends, or patterns that emerged from your study. Ensure that you mention the most important aspects that contribute to the overall objective of your research.

Reiteration of Research Objective: Restate the objective or purpose of your research to remind the reader of the problem you aimed to address or the knowledge gap you aimed to fill. Emphasize how your findings have successfully addressed the research question or objective stated in the introduction.

Significance and Contribution: Highlight the significance and contribution of your research within the broader context of the field. Explain how your study has advanced knowledge, improved methodologies, or provided valuable insights into the topic. Discuss any implications your findings may have for theory, practice, or policy in the field.

Limitations: Acknowledge the limitations of your study. Address more constraints or challenges that may have affected the research process or the interpretation of results. This demonstrates your awareness of the study's boundaries and adds credibility to your work.

Recommendations for Future Work: Suggest areas for further research or specific directions that future studies could take based on the findings and limitations of your research. Highlight potential avenues for expanding or building upon your work to enhance understanding, address unanswered questions, or improve methodologies.

Conclusion Statement: Conclude your paper by summarizing the overall significance and impact of your research. Restate the main takeaway message or key points you want the reader to remember after reading your paper. Leave a lasting impression by reinforcing the importance of your work and its potential implications for the field.

Avoid Introducing New Information: Ensure that your conclusion does not introduce new data, arguments, or information that has not been discussed in the body of your paper. The conclusion should serve as a synthesis and reflection of the research you have already presented.

Other comment:

Surrogate Selection and Justification: While 2-POE is appropriately chosen for its semi-volatility (better simulating drugs like cyclophosphamide), the manuscript could better explain why 2.5% w/v specifically. Is this the maximum soluble concentration without saturation, or clinically relevant (e.g., mimicking drug doses)? Compatibility testing with CSTD materials (e.g., no degradation of seals/tubing) is mentioned briefly in responses to reviewers but not in the main text—expand this, as solvents like IPA have known incompatibilities (e.g., per Simplivia white papers). Also, discuss potential limitations: 2-POE's aqueous solubility may not fully represent lipophilic drugs.

Clarity and Redundancy: The introduction and discussion repeat arguments on surrogate volatility (e.g., IPA vs. 2-POE) across sections, making the manuscript lengthy (as noted by Reviewer #1). Consolidate into a dedicated subsection. Transitions between surrogate rationale and methods could be smoother (e.g., "Based on HSE and NIOSH guidelines, we selected 2.5% w/v 2-POE..."). Abstract lacks explicit mention of replicates (n=4) and statistical methods (e.g., ANOVA)—add for completeness.

Data Availability and Reproducibility: Raw CDS data (Chromeleon v7.3) are not manipulable outside the software, as noted, but provide access instructions or deposit processed data (e.g., peak areas, calibration curves) in a repository like Figshare. Supplementary materials (e.g., detailed IFU adherence, chamber diagrams) are referenced but not fully described—ensure they include all protocols for replication.

Statistical Analysis: Appropriate (e.g., 95% CI for blanks; t-tests for comparisons), but clarify handling of <loq values="">Limitations Not Fully Addressed:

o Chamber volume (125 L) is fixed, but real-world compounding occurs in variable environments (e.g., biological safety cabinets)—discuss generalizability.

o No assessment of liquid spills or surface contamination; focus is vapor only—note this as a scope limitation.

o Task 2 modification (100 mL bag) is justified, but quantify any impact on results (e.g., vs. standard NIOSH setup).

o Potential bias: Authors collaborated on the 2016 protocol development—disclose if any CSTD manufacturers influenced selection.

Minor Technical Issues:

o Units: Consistently use "ppb" (parts per billion) vs. "ppbv" (parts per billion by volume).

o Figures/Tables: Ensure all (e.g., Figure 3 copyright issue resolved) are high-resolution and labeled.

o Language: Generally clear, but some sentences are dense (e.g., abstract methods)—break for readability.

Specific Suggestions for Improvement

• Abstract: Add: "Based on four replicates per task/device, with ANOVA for comparisons..." and briefly justify 2-POE ("a semi-volatile surrogate mimicking hazardous drugs").

• Introduction: Consolidate surrogate discussion (pp. 14-15) and add transition sentence as suggested in reviewer responses.

• Methods: Expand on 2-POE concentration (e.g., "2.5% w/v represents maximum aqueous solubility and clinical relevance without material incompatibility"). Confirm IFU adherence explicitly.

• Results/Discussion: Include a figure plotting release values (even if <loq) comparison.="" discuss="" for="" practical="" thresholds:="" visual="" what="">• Conclusions: Emphasize implications for USP <800> compliance and future NIOSH updates.

• General: Shorten by ~10-15% by removing redundancies; aim for <8,000 words.

Additional Comments

• No ethical concerns (e.g., no human subjects; devices tested in vitro).

• No plagiarism detected; citations are appropriate (e.g., HSE MDHS 104, NIOSH docket).

• This work could influence policy—suggest authors submit to NIOSH for protocol refinement.

Best Regards.</loq)></loq>

**********

what does this mean?). If published, this will include your full peer review and any attached files.

If you choose “no”, your identity will remain anonymous but your review may still be made public.

**Do you want your identity to be public for this peer review?**  For information about this choice, including consent withdrawal, please see our Privacy Policy

Reviewer #2: No

Reviewer #3: No

Reviewer #4: No

Reviewer #5: No

Reviewer #6: No

**********

[NOTE: If reviewer comments were submitted as an attachment file, they will be attached to this email and accessible via the submission site. Please log into your account, locate the manuscript record, and check for the action link "View Attachments". If this link does not appear, there are no attachment files.]

To ensure your figures meet our technical requirements, please review our figure guidelines: https://journals.plos.org/plosone/s/figures

You may also use PLOS’s free figure tool, NAAS, to help you prepare publication quality figures: https://journals.plos.org/plosone/s/figures#loc-tools-for-figure-preparation.

NAAS will assess whether your figures meet our technical requirements by comparing each figure against our figure specifications.

# Author comment: Author response to Decision Letter 2

**DOI:** 10.1371/journal.pone.0337729.r004

21 Jul 2026

PONE-D-25-12716R1

Head-to-head containment performance testing of four commercially available closed-system transfer devices using the 2016 Draft NIOSH test protocol and 2.5% w/v 2-phenoxyethanol as challenge agent.

Dear PLOS One

Please find below the authors rebuttal responses to reviewers questions for the above manuscript.

Please find included the following items and files:

• A letter that responds to each point raised by the academic editor and reviewer(s). This letter is a separate file labelled 'Response to Reviewers'.

• A marked-up copy of the latest manuscript that highlights changes made to the original version. This file is labelled 'Revised Manuscript with Track Changes'.

• An unmarked version of the revised paper without tracked changes. This is a separate file labelled 'Manuscript'.

Authors Responses to Reviewers Questions:

Comments to the Author (ASW)

1. If the authors have adequately addressed your comments raised in a previous round of review and you feel that this manuscript is now acceptable for publication, you may indicate that here to bypass the “Comments to the Author” section, enter your conflict of interest statement in the “Confidential to Editor” section, and submit your "Accept" recommendation.

Reviewer #2: All comments have been addressed

Reviewer #3: (No Response)

Reviewer #4: All comments have been addressed

Reviewer #5: All comments have been addressed

Reviewer #6: All comments have been addressed

ASW Response: Nothing to address here.

________________________________________

2. Is the manuscript technically sound, and do the data support the conclusions?

The manuscript must describe a technically sound piece of scientific research with data that supports the conclusions. Experiments must have been conducted rigorously, with appropriate controls, replication, and sample sizes. The conclusions must be drawn appropriately based on the data presented.

Reviewer #2: Yes

Reviewer #3: (No Response)

Reviewer #4: Yes

Reviewer #5: Yes

Reviewer #6: Yes

ASW Response: Nothing to address here.

________________________________________

3. Has the statistical analysis been performed appropriately and rigorously?

Reviewer #2: Yes

Reviewer #3: (No Response)

Reviewer #4: Yes

Reviewer #5: Yes

Reviewer #6: No

ASW Response: Nothing to address here. Reviewer 6 has responded No. The author respectively would like to respond to reviewer 6 to say that as all recorded values for 2-POE concentration were below the lower limit for quantitation (<LLOQ) then there is no valid statistical treatment of the data possible. The only statistical analysis that is pertinent and has been followed and included by the authors is the fitting of the analytical calibration data for 2-POE using linear regression analysis.

________________________________________

4. Have the authors made all data underlying the findings in their manuscript fully available?

The PLOS Data policy requires authors to make all data underlying the findings described in their manuscript fully available without restriction, with rare exception (please refer to the Data Availability Statement in the manuscript PDF file). The data should be provided as part of the manuscript or its supporting information, or deposited to a public repository. For example, in addition to summary statistics, the data points behind means, medians and variance measures should be available. If there are restrictions on publicly sharing data—e.g. participant privacy or use of data from a third party—those must be specified.

Reviewer #2: Yes

Reviewer #3: (No Response)

Reviewer #4: Yes

Reviewer #5: Yes

Reviewer #6: Yes

ASW Response: Nothing to address here.

________________________________________

5. Is the manuscript presented in an intelligible fashion and written in standard English?

PLOS ONE does not copyedit accepted manuscripts, so the language in submitted articles must be clear, correct, and unambiguous. Any typographical or grammatical errors should be corrected at revision, so please note any specific errors here.

Reviewer #2: Yes

Reviewer #3: (No Response)

Reviewer #4: Yes

Reviewer #5: Yes

Reviewer #6: Yes

ASW Response: Nothing to address here.

________________________________________

6. Review Comments to the Author

Please use the space provided to explain your answers to the questions above. You may also include additional comments for the author, including concerns about dual publication, research ethics, or publication ethics. (Please upload your review as an attachment if it exceeds 20,000 characters)

Reviewer #2: Thank you for addressing the comments. You have thoroughly addressed all prior concerns with clear, thoughtful revisions that significantly strengthen the manuscript. The introduction now provides a more cohesive rationale for the study, and the methods section has been expanded with essential technical details—particularly regarding analytical procedures, replication, and statistical analysis—ensuring reproducibility. The inclusion of a dedicated limitations section adds necessary context to the findings, enhancing transparency and scientific rigor. Clarifications around surrogate selection, data handling, and device testing protocols align well with PLOS ONE’s standards. The discussion appropriately interprets results in light of real-world implications for healthcare worker safety and CSTD performance evaluation. All figures and tables are now accurately referenced and clearly presented, with consistent units and annotations. Most importantly, the authors have maintained a balanced, evidence-based tone throughout, avoiding overstatement while still highlighting the study’s value in enabling direct comparison between air-cleaning and barrier CSTD technologies. The financial disclosure and competing interests statements are now fully transparent, confirming that the funders had no role in study design or data interpretation. These revisions have transformed the manuscript into a robust, well-executed study that makes a valuable contribution to occupational safety in healthcare settings

Author Response: Thank you Reviewer 2 for your thoughtful and balanced review of the manuscript and recent changes and for recommending this to go forward for publication. We appreciate your time in reading the revised manuscript and diligence in review.

Reviewer #3: This manuscript presents a head-to-head containment performance comparison of four commercially available Closed System Transfer Devices (CSTDs) using the 2016 Draft NIOSH test protocol and 2.5% w/v 2-phenoxyethanol (2-POE) as a surrogate. The study is methodologically sound, builds upon previously published and NIOSH-adopted protocols, and provides quantitative containment data for both physical barrier and air-cleaning CSTDs. The conclusions are well-supported by the data. However, the manuscript is excessively long and repetitive, particularly in the Introduction and Discussion sections. Several clarifications and improvements in data presentation, statistical reporting, and focus are needed before publication in this journal.

Author Response: Thank you Reviewer 3 for your balanced responses which are very supportive of this being a scientifically robust piece of research. Reviewer 3 comments regarding the length of the manuscript are on point and 100% aligned with a reviewer in the previous round of peer review. Unfortunately, although the excessive and repetitive sections were removed especially in the introduction and discussion sections along with clarifications and improvements in data presentation the version with these changes submitted does not appear to have been reviewed. The attached is a re-submission and so hopefully all of the changes that should have been included last time are now completed in this submission. We would like to thank you again for raising this point because it allowed us to identify this issue.

As mentioned above in our rebuttal to Reviewer 6 in terms of statistical reporting there is no appropriate statistical treatment that can be applied to the results data as all data is below the limit of quantitation (<LLOQ) and as such the numbers cannot be subject to any further statistical treatment. All results data were found to be below the limit at which the instrument can attribute a value using the external calibration curve. The author wishes to thank the reviewer once again for their time and consideration in reviewing the revised article.

Introduction

The introduction is generally sound but could be more focused. Currently, it provides extensive background on general populations before narrowing to the target group. I suggest tightening the narrative to more directly foreground the study population and research gap. Additionally, please incorporate recent, relevant references to strengthen the theoretical foundation, such as:

Author Response:

The author appreciates the reviewer recommendations and the revised manuscript has now been tightened up with significant removal of surplus text and discussion. We hope that this satisfies the reviewer. The below recommended articles suggested by the reviewer to strengthen the theoretical foundations are mostly all relating to Iranian oil field work and are not relevant to the subject matter for the submitted manuscript. One is related to shift work related disorders in a hospital and is therefore not considered relevant to the subject of the manuscript.

DOI: 10.18502/ijph.v52i10.13854

Iran J Public Health. 2023 Oct;52(10):2157-2168. doi: 10.18502/ijph.v52i10.13854.

Workplace Risk Factors Assessment in North-Azadegan Oil Field Based on Harmful Agents Risk Priority Index (HARPI)

DOI: 10.3923/rjmsci.2016.111.115

Phenol Removal from Aqueous Environment by Adsorption onto Pomegranate Peel Carbon

November 2016Electronic physician 8(11):3248-3256

DOI: 10.3233/WOR-220077

Semi-quantitative risk assessment for workers exposed to occupational harmful agents in an oilfield in Iran

doi: 10.3233/WOR-203165

Shift working disorders among nurses of Tehran hospital and its related factors in 2016

https://www.makhillpublications.co/public/files/published-files/mak-rjas/2016/7-547-553.pdf

Relationship between Job Content and Demographic Variables with Musculoskeletal Disorders among Nurses in a University Hospital, Qom Province, 2014

In summary the authors thank the reviewer for their input and recommendations and have tightened up the submitted manuscript but have decided not to include the suggested references on the grounds that they are not relevant to the subject matter.

At the continue 10 major and minor comment. : Major Comments

### **10 MAJOR COMMENTS**

1. **Excessive Length & Repetition**

The manuscript is unnecessarily long (over 120 pages), with significant repetition, especially regarding the rationale for surrogate selection and criticisms of the 2015 NIOSH protocol. The authors should consolidate repetitive sections (e.g., surrogate selection arguments) and move detailed methodological descriptions to Supplementary Materials.

Author Response: The revised manuscript has been significantly shortened with large sections of text and discussion removed especially on surrogate selection. The author previously made these alterations but for some reason the long version was reviewed. This is now addressed fully in the revised manuscript.

Hopefully the revised text meets the reviewers expectations for reduced word count and is now less than 30 pages in length. The author has removed details on surrogate selection and the critique of the 2015 draft NIOSH protocol. The authors view remains that as the 2015 Draft NIOSH protocol related publications are increasing at pace it is important to highlight the lack of scientific rigour and inability of this protocol to quantify releases of Isopropyl alcohol as surrogate because the results generated by researchers employing this protocol are meaningless and voided. This is actually quite dangerous for those making decisions based on containment performance from the 2015 Draft NIOSH test protocol. That said the author is happy to remove this critique and it is now removed from the manuscript.

There is some contradictory feedback between this reviewer and other reviewers with respect to placement of additional details for methodology with some reviewer comments requesting more detail within the main body of the article and some asking for this to be placed in supplementary materials which is where most of the details sit currently. The author view is that there is a good balance in detail supplied in the main body of the article to enable reproduction of the testing with references to additional sources of information included and the supplemental materials. It would be impossible to please all of the reviewers on this point but I hope that I have satisfied the current Reviewer in achieving the correct balance of detail for the methodology and thank them once again for their input here.

2. **Lack of Clear Research Hypothesis & Objectives**

While the objective is stated in the Abstract, the main text does not clearly articulate a testable hypothesis. The study appears descriptive rather than hypothesis-driven. A clearer framing of the research question would strengthen the paper.

Author Response: The author thanks the reviewer for their opinion and view on the strength of the objective however the objectives are clearly set out as being a head to head comparison of the containment performance of four commercially available closed system transfer devices which is very clear. The hypothesis is simply can each type of tested CSTD device contain the surrogate 2-POE. I do not think that this needs to be re-framed and this would not improve the article however we have added in the wording “The null hypothesis for the study is that the CSTDs under test are not able to completely contain the 2-POE surrogate whilst carrying out NIOSH Task 1 and Task 2..”

3. **Insufficient Statistical Detail**

The authors state that “no statistically significant difference was found,” but they do not report the specific statistical tests used, p-values, or effect sizes. Since all results were below the LOQ, the appropriateness of statistical comparisons should be justified or clearly stated that formal testing was not performed.

Author Response: The author thanks the reviewer for this comment and I believe is suggesting as does the author that as all values were recorded as being below LLOQ then there is no appropriate statistical treatment that can be applied to the results data. It is therefore fair to state that there was no difference found between the containment performance of the two types of CSTD evaluated because they both gave below LLOQ values. The only statistical tests employed in the study were linear regression for the calibration curve for 2-POE response versus amount and calculation of means of each cohort of replicates for each CSTD and NIOSH Task. Any other test such as ANOVA would only have been appropriate if the test data had produced above LLOQ i.e above quantifiable limits release data.

4. **Data Availability Statement is Inadequate**

The authors claim data are available “within the manuscript and Supporting Information,” but the raw instrument data (Chromeleon files) are not accessible. This violates PLOS ONE’s data policy. They should deposit raw data in a public repository or provide processed data in a usable format (e.g., CSV).

Author Response: The author thanks the reviewer for this comment. The Author has made all processed data available in the manuscript and as the reviewer has pointed out proprietary data handling software in Chromeleon CM7.3 (very high cost) was used to collect and process the data. This software is proprietary and is designed to work within a highly regulated laboratory environment under strict data handling protocols. As such it is not possible to access the RAW instrument data which would also be extremely difficult to handle or file transfer due to the extremely large file sizes involved in CDS mass spectrometry data. Attempts to copy the data may also lead to the possibility of corruption of the RAW data or inclusion of “mistakes” and this might lead to inaccuracies and for third party researchers to then perform compromised analysis on the compromised data. All processing was done using the internal algorithms inside of Chromeleon CM7.3 which is an industry standard rather than in third party programs such as excel. The authors therefore believe that sufficient sharing of the CDS processed data has performed and the data as it is, has been made available and put in the public domain in accordance with Plos one data policy. Also given that the data provides limited opportunity for re-processing due to it being below LLOQ and in many cases below LOD the lack of RAW data is not disadvantaging the scientific community engaged in this type of work from benefiting from the data. The authors have also checked all of the main scientific data repositories and the only ones available do not accept Chromeleon data files or GCMS data files unless linked to metabolics studies which are able to be filed.

5. **Overemphasis on Protocol Validation**

A large portion of the Discussion is devoted to defending the 2016 NIOSH protocol and critiquing other methods. While context is important, the focus should shift toward interpreting the *results* of this study and their implications for CSTD selection and healthcare worker safety.

Author Response: The author thanks the reviewer for this comment. This has been addressed and the text removed including any critique of the earlier 2015 Draft NIOSH test protocol and a focus more on the actual study results data and implications for healthcare workers. We would like to thank the reviewer for their comment on this aspect of the manuscript.

6. **Limited Discussion of Practical Significance**

The clinical or occupational health implications of the findings are underdeveloped. What does “below LOQ” mean in terms of real-world exposure risk? How should end-users interpret these results when selecting a CSTD?

Author Response: The author thanks the reviewer for this comment which makes a valid point. Unfortunately it is impossible for the author to comment on the exposure risk to healthcare workers when below LLOQ concentrations of 2-POE (the surrogate for hazardous drug materials) are reported. The fact that we do not yet have instrumentation capable of detecting at significantly below parts per billion volume levels (ppbv) does not mean that these levels could not be causing harm. We simply do not know. The key conclusions the authors make are that with the four different CSTDs evaluated in the study they all gave below LLOQ results and so can be regarded as being equally capable of offering a level of protection to healthcare workers when the CSTDs tested are used with hazardous drugs as compared with an open system of needle and syringe. We thank the reviewer for this very thought provoking commentary and on another platform/ forum, if word count and circumstance allowed there should be a larger discussion around what these studies mean in terms of protection and real-world exposure risk involving hazardous drugs. The authors are keen to point out that there is a good rationale for using a semi-volatile material such as 2-POE to mimic hazardous drugs because it exhibits similar behaviour to other semi-volatile materials when they are released in liquid droplet form (which arguably represents the highest risk in terms of potential exposure). Unfortunately, because the hazardous drugs have such low chemical concentration in the vapour they are currently impossible to detect directly in the vapour using the NIOSH approach. By using a surrogate approach with a safety factor of 100 if we are detecting 1ppbv of the surrogate then in theory that might mean the hazardous drug release resulting from a similar release would be one hundredth of this value or 10 parts per trillion volume but this is a leap of faith and we cannot be sure that that would be the case. In simple terms if there is 2-POE detected from release when using the CSTD and this is above the lower limit for quantification (LLOQ) then we can compare this with releases when using an open system of needle and syringe which are also well above the LLOQ for 5 microlitre and 10 microlitre release volumes. This allows an elementary way of expressing how much better the CSTD is doing compared with no CSTD or having an open system. When all of the release values are below the LLOQ then we cannot really comment except to say that the CSTD should offer significant improvement in operator protection as compared with an open system of needle and syringe – providing the hazardous drug materials behave in an analogous manner to the surrogate. The caveat at the end is important for surrogates that are very volatile because these materials do not behave analogous to hazardous drugs and releases involving isopropyl alcohol or ethanol will evaporate very quickly and achieve 100% of their theoretical saturated vapour concentration whereas we know that materials that are semi-volatile will not achieve much more than ~1% of their SVC. This could over-estimate the occupational risk when using volatile surrogates by another factor of 100 over the safety factor of 100 making the interpretation of results in terms of occupational exposure less meaningful.

7. **Sample Size Justification**

The use of n=4 replicates is based on the NIOSH protocol but is not statistically justified. A power calculation or justification for the sample size should be provided, especially given that all results were below LOQ.

Author Response: The author thanks the reviewer for this interesting comment. The authors are not attempting to justify the sample size as this is taken directly from the 2016 Draft NIOSH universal test protocol. The authors study design was to follow this test protocol which we did. The difficulty in performing larger numbers of tests is that the testing would become prohibitively expensive to perform for third party researchers such as university students and one of the purposes of dissemination of the work is to allow a greater number of scientific researchers access to the protocols and methodology to perform their own independent testing. The authors do not think that a power calculation would be meaningful in this context and would break the link with performing the testing in accordance with the requirements of the 2016 Draft NIOSH universal test protocol. A further point is that as the number of tests increase the number of days over which the testing is performed increases and the background levels of various semi-volatile and volatile materials may fluctuate to a greater extent when increasing the overall time line for testing. This would impact the quality of the data and we might observe an increased trend in background when performing much larger replicates of tests in a single cohort. This can be observed in the 2018 publication in Plos one by the same authors, here the LLOQ level was almost twice that obtained in the more recent submitted study. This is an unintended consequence of higher background levels of 2-POE that are so subtle that they are not detected in the background performed immediately before test but that do contribute to an increase in the LLOQ (here by a factor of 2!). With an increased LLOQ it may prevent discrimination between two different CSTDs, one that is just above the LLOQ and one that remains below the LLOQ and so controlling the background levels of the surrogate 2-POE become incredibly important in this type of testing. The authors validated a cleaning protocol and performed robust cleaning of the NIOSH chamber in between each test but simply the fact that many more tests were performed around the time of the 2018 Plos one publication testing the LLOQ for 2-POE was double that presented in the current article.

8. **Incomplete Reporting of Methodological Details**

Key details (e.g., ATD-GC-MS parameters, column temperatures, desorption times) are relegated to Supplementary Materials but are critical for reproducibility. Essential parameters should be summarized in the main Methods section.

Author Response: The author thanks the reviewer for this comment. Please see above the justification for putting all of the details of the instrument program in the supplementary file. The article is already 30 pages in length and it is appropriate to include the more basic experimental detail in the main body of the methods section for such complex analyses. You will find all of the details for running the chromatography method in the supplementary file attached. Temperatures, gas flow rates etc.

9. **Potential Conflict of Interest**

The study was funded by ICU Medical, the manufacturer of two tested devices (ChemoLock™). Although the authors state the funder had no role in study design or analysis, the potential for bias should be more transparently discussed in the Limitations section.

Author Response: The author thanks the reviewer for this comment. The authors are experienced academics and are used to working on projects that have external funding. Funders never have anything to do with the studies. Funders also expect and demand that this is the case because they value the independence of the work being carried out by an external group otherwise they would simply perform the study internally. As with most scientific research projects there was some financial assistance from a commercial third party, but they did not have any input in the study design, execution, data analysis, or writing of the manuscript. The authors would also like to point out that the findings demonstrate equivalency of containment performance to other competitor devices which is not in the interests of the named commercial third party. I think this should resolve any concerns the reviewer might have in terms of bias.

10. **No Dedicated Limitations Section**

The manuscript lacks a structured Limitations section. Important limitations include the use of a single surrogate concentration, the absence of real-world validation, the small number of CSTD models tested, and the lack of exposure-risk correlation.

Author Response: The author thanks the reviewer for this comment. The experimental work presented follows on from work previously published in 2018 using 2-POE and more extensive work performed using the surrogate of 2-POE in water by the UK Health and Safety Executive (HSE) which is cited in the submitted manuscript. The choice of the single concentration of 2-POE is not seen as a limitation of the study. It does not limit the capability to detect microlitre and nanolitre scale liquid releases of surrogate and airborne chemical concentrations down to below one parts per billion volume (ppbv) which is state of the art for detection of semi-volatile materials in the air/ vapour. A key study design rule was not to exceed or approach the limit of solubility of the surrogate 2-POE but to provide highly sensitive measureable chemical vapour concentrations from microlitre scale liquid releases inside the NIOSH chamber down to sub parts per billion volume (ppbv) levels which is the state of the art in terms of both selectivity and sensitivity. Higher concentrations of the surrogate 2-POE may also cause incompatibility issues with the equipment under test just as 70% isopropyl alcohol has been observed to damage many different types of medical grade plastics used in the construction of medical devices. We have done some research (unpublished data) on this aspect and not found compatibility issues with 2-POE and CSTDs over a range of concentrations but have found serious issues with 70% IPA and other previously proposed NIOSH surrogates and so it is a key consideration when selecting the concentrations of a surrogate for use in NIOSH testing. The golden rule is that you do not want the test surrogate to have an impact on the outcome of the testing – is should be inert and therefore play no part or influence the results. The authors are confident that at 2.5 % w/v 2-POE is a suitable surrogate, remains inert and provides robust scientific data in containment performance testing of CSTDs. It is also a relevant concentration in terms of drug concentration in aqueous solution. 70% IPA is not!

In terms of exposure risk correlation, it is beyond the scope of the study to attempt to correlate the release data for 2-POE to exposure of healthcare workers when handling hazardous drugs. The study is aimed to demonstrate the performance in containment of two different types of CSTD when using 2-POE as surrogate in the 2016 Draft NIOSH universal test protocol which it does. There is a design safety factor of 100 in terms of theoretical SVC for 2-POE over thiotepa the most volatile hazardous drug and the relationship between surrogate and hazardous drugs in terms of theoretical SVC can provide a “guess” at what a hazardous drug concentration might be in the vapour from a similar size liquid release of hazardous drug if you are able to achieve above LLOQ releases with 2-POE. With the study data we were always below LLOQ.

---

### **10 MINOR COMMENTS**

1. **Abstract Clarity**

The Abstract should briefly state *why* 2-POE was chosen as the surrogate. Add one sentence on its semi-volatile properties and relevance to hazardous drugs.

Author Response: The author thanks the reviewer for this comment. We were previously asked by a reviewer to remove discussion around selection of 2-POE. Further, the choice of 2-POE is detailed in the 2016 Draft NIOSH universal test protocol for the reader to access if they wish to. The manuscript also builds on a previous publication and works using 2-POE in combination with the 2016 Draft NIOSH universal test protocol for assessment of containment performance and this is cited by the authors and provides the relevant justification for selection. The authors also discuss in the main body of the manuscript the work by Pengelly and Wheeler et al at the Health and Safety Executive (HSE) using 2-POE as a surrogate for hazardous pesticides in real-world experiment scenarios which again points in to the work already done to demonstrate the strengths of using semi-volatile material such as 2-POE as surrogate.

2. **Figure & Table Referencing**

Some figures (e.g., Figure 3) are not clearly referenced in the text. All figures and tables should be explicitly cited in the order they appear.

Author Response: The author thanks the reviewer for this comment. All figures are clearly referenced in the text. We apologise if you may be looking at an earlier version of the manuscript but this I can assure you has been fully addressed.

3. **Typos and Inconsistencies**

- Page 75, Conclusions: “NIOSH Task 2each” → “NIOSH Task 2 each.”

- Page 96: “was-based-on It is” → “It is.”

- Page 107: “In feetfact” → “In fact.”

Author Response: The author thanks the reviewer for this comment on typographical errors and these have been addressed in the latest manuscript.

4. **Units Consistency**

Use “ppbv” or “ppb” consistently. In some places, “ppb” is used without specifying volume.

Author Response: The author thanks the reviewer for this comment on typographical errors and these have been addressed in the latest manuscript. All changed to ppbv and LLOQ.

5. **Positive Control Reporting**

Positive controls were performed at 5 and 10 µL, but the text references an ability to quantify down to ~100 nL. Clarify the relationship between the reported LOQ (0.41 ppb) and the minimum quantifiable release volume.

Author Response: The author thanks the reviewer for this comment. The system suitability tests were performed at 5 microlitre and 10 microlitre volume levels as these are microlitre size releases and so are at an appropriately small level for demonstrating detection sensitivity and are practicable to be performed. The performance of the analytical method is subject to the background level of 2-POE analysed prior to each test (in duplicate), the chamber background values (n=85) were processed using standard methodology and used to calculate the LLOQ of 0.41 ppbv. This chamber concentration can then be directly related to a microlitre or nanolitre scale liquid release of 2-POE surrogate which equates to a 100 nL release at this concentration level.

6. **Reference Formatting**

Some references are incomplete (e.g., Ref. 30–33 are “private communication” or presentations). Replace or cite formally where possible.

Author Response: The author thanks the reviewer for this comment. There is no formalised way of citing these presentations/ communications.

7. **Repetitive Language in Introduction**

The Introduction repeats points about USP <800> and NIOSH protocols. Streamline to improve flow.

Author Response: The author thanks the reviewer for this comment and the manuscript has been streamlined considerably in terms of removing repeated comments and sections.

8. **Conclusion Restructuring**

The Conclusion is lengthy and reiterates the Discussion. Condense to a clear summary of findings, implications, and future research.

Author Response: The author thanks the reviewer for this comment. The conclusion has been revised and presents a concise summary of the key experimental data.

9. **Definition of LOQ/LLOQ**

The terms LOQ and LLOQ are used interchangeably. Use one term consistently.

Author Response: The author thanks the reviewer for this comment on typographical errors around LLOQ and LOQ and these have been addressed in the latest manuscript.

10. **Acknowledgments Section**

The funding statement is repeated in multiple places. Consolidate into a single Financial Disclosure section.

Author Response: The author thanks the reviewer for this comment. This has been addressed.

---

**Recommendation**

**Major Revision Required.** The manuscript presents valuable and robust containment data for CSTDs using an advanced NIOSH protocol. However, it requires significant condensation, improved statistical reporting, clearer data availability, and a sharper focus on the study’s findings and implications before it is suitable for publication in this journal.

Discussion Depth

The current discussion is somewhat superficial. Please provide a more in-depth interpretation of findings in relation to existing literature, integrating the updated references above.

I look forward to reviewing the revised manuscript. Please address all points comprehensively in your response letter and manuscript track changes.

Please update and ensure accurate, complete citations with DOIs. Incorporate recent literature to strengthen the discussion, such as:

DOI: 10.18502/ijph.v52i10.13854

DOI: 10.3923/rjmsci.2016.111.115

DOI: 10.3233/WOR-220077

doi: 10.3233/WOR-203165

---

Author Response: The author thanks the reviewer for this comment however the recommended citations are not relevant to this study. The author believes that all other matters have been addressed critically reduction in word count and length with removal of large sections of text and discussion and tightening of the manuscript to appeal to a wider readership.

Reviewer #4: The article mentions: "No care over of 2-POE was observed in the chamber following testing, and there is no additional cleaning of the chamber was required between tests”

However, there is no clear explanation here on how to verify 'no residue'. Is it through visual inspection, cabin air sampling, or surface wiping? If air sampling is used, is the detection limit low enough to ensure that there are no low-level residual effects on subsequent testing?

Improvement suggestion: Can you briefly explain the specific method for verifying "no residue" (e.g. "verifying by sampling the cabin with standard air and confirming that the 2-POE concentration is below the detection limit after testing") to enhance the credibility of this statement.

Author Response: The author thanks the reviewer for this comment. The stated detection limit (LOD) of the instrumental method demonstrates that the chamber blank samples were consistently below this figure and therefore acceptable. The authors did not observe an increase in chamber backgrounds, LOD or LLOQ with increasing numbers of tests which demonstrates that there is not an accumulation or carry over of 2-POE between tests.

Reviewer #5: Comments to the Author

1. Abstract

• The abstract clearly states objectives, methods, and key findings. However, it would benefit from briefly noting why 2.5% w/v 2-POE was selected over other surrogates, reinforcing the methodological rigor.

Author Response: The author thanks the reviewer for this comment but there is no room in the word count to go into selection criteria for the surrogate 2-POE. This could not be covered in a brief sentence. The author believes this aspect of the study design is covered in the main body of the manuscript as well as previous publications including from the same authors 2018 (Plos One) and in the 2016 Draft NIOSH Universal test protocol for containment performance of CSTDs published by the NIOSH. For brevity it is kept out of the abstract.

• Inclusion of “n=4 replicates” is appropriate, but specifying that results were below LOQ for all devices quickly highlights the high containment performance observed.

Author Response: The author thanks the reviewer for this comment but is unsure of the point the reviewer is trying to make here.

2. Introduction

• The background effectively establishes the importance of CSTDs and the evolution of NIOSH protocols. However, the section could be tightened to reduce repetition regarding surrogate volatility.

• The rationale for selecting 2-POE over IPA is well-supported by references to HSE and NIOSH documents, strengthening the scientific premise.

3. Methods

• The adherence to the 2016 NIOSH draft protocol and manufacturer IFUs is a strength, ensuring consistency and validity.

• Clarification on the chemical compatibility testing of 2.5% w/v 2-POE with CSTD materials would enhance methodological transparency.

Author Response: The author thanks the reviewer for this comment on compatibility, but the authors felt that this was outside of the scope of the manuscript. The compatibility aspect of surrogates is something that the NIOSH has covered in its town hall meeting in 2016 where ICU medical presented data on this demonstrating the compatibility of 2-POE with the plastics used in CSTD medical devices.

• While replicate number (n=4) is stated, a brief justification for this sample size in relation to statistical power would be useful.

Author Response: The author thanks the reviewer for this comment but the authors are simply replicating the protocols published by the NIOSH and are not making a comment on the appropriateness of the number of replicates within the protocol.

4. Results

• Data presentation in Table 4 is clear, showing all devices performed below LLOQ. The use of confidence intervals adds robustness.

• Positive and negative control results suitably demonstrate system suitability and low background interference.

• The ability to quantify releases down to ~650 nL is a notable technical achievement and should be emphasized as a key outcome.

5. Discussion and Conclusion

• The discussion appropriately interprets the parity between air-cleaning and barrier CSTDs, a significant finding for OHS practice.

• However, the practical implications for healthcare worker exposure risk are underdeveloped; linking LOQ values to potential hazard thresholds would strengthen relevance.

Author Response: The author thanks the reviewer for this comment on linkage between the study findings using 2-POE to assess containment performance and healthcare worker exposure risk. The authors believe that this discussion sits outside of the objectives of the study and manuscript but thanks the reviewer for raising this thought-provoking point. We are happy to report that of the four types of CSTD assessed in the study we could not quantify any release of 2-POE indicating good levels of containment of all devices tested.

• The conclusion rightly advocates for standardized semi-volatile surrogate use, but could more explicitly call for NIOSH to finalize the 2016 protocol based on this evidence.

Author Response: The author thanks the reviewer for this comment and it is a very good point indeed but the authors were keen to not get involved in any politics and stay within the scientific parameters and focus on the key findings of the study. We do agree that it would be extremely helpful if the NIOSH were to finalise a protocol and bring some clarity to the area. We in the mean time are happy to attempt to bring some robust scientific data to the pharmacy and nursing communities to help inform them when choosing an appropriate CSTD technology in their work flow and clinical practice.

6. References

• References are comprehensive and appropriate, covering key regulatory documents and prior research.

• Including a citation for the chemical compatibility claim between 2-POE and medical device plastics would further support the methods.

Author Response: The author thanks the reviewer for this comment. The author has added in a reference on chemical compatibility with different organic solvent materials.

Final Recommendation

Minor Revisions. The manuscript presents a technically sound, well-controlled study that advances the evaluation of CSTD containment performance. The methodology is robust, data support the conclusions, and the work has direct relevance to occupational health practice. Revisions should focus on refining the discussion of practical implications, clarifying methodological justifications, and minor editorial tightening to enhance clarity.

Author Response: The author thanks the reviewer for their extensive and comprehensive comments.

Reviewer #6: Ref.: PONE-D-25-12716R1

Title:

Head-to-head containment performance testing of four commercially available closed-system transfer devices using the 2016 Draft NIOSH test protocol and 2.5% w/v 2-phenoxyethanol as challenge agent

Dear editor

Greetings

Thank you for giving me the opportunity to review this manuscript. Thanks to the valuable efforts of the authors. This study is a practical study whose findings can lead to proper awareness for the society. This manuscript presents a head-to-head comparison of four closed-system transfer devices (CSTDs)—two physical barrier types (ChemoLock™ barrier and BD PhaSeal™) and two air-cleaning types (ChemoLock™ air-cleaning and Tevadaptor™)—using the 2016 Draft NIOSH universal protocol for assessing containment performance during hazardous drug compounding and administration. The challenge agent is 2.5% w/v 2-phenoxyethanol (2-POE) in water, analyzed via automated thermal desorption gas chromatography-mass spectrometry (ATD-GC-MS). The study concludes that all devices perform below the limit of quantification (LOQ; 0.41 ppb), with no significant differences between barrier and air-cleaning types, suggesting equivalent containment efficacy.

The research is technically sound, timely, and relevant to occupational health, particularly in oncology and pharmacy settings where CSTDs are mandated under USP <800> to minimize exposure to hazardous drugs. It builds on the authors' prior work (e.g., Wilkinson et al., 2018 in PLOS ONE) and addresses gaps in CSTD evaluation by emphasizing semi-volatile surrogates over volatile ones like isopropyl alcohol (IPA). The data support the conclusions, with appropriate controls, replicates (n=4 per task per device), and statistical analysis. However, minor issues in clarity, surrogate justification, and data presentation warrant revisions for improved readability and reproducibility.

Author Response: The author thanks the reviewer for this comment on recommended improvements. The Authors have attempted to address these areas in accordance with all reviewer comments in this round and previous rounds (three lots of duplicate reviewers, making six in total)

However, there are a number of comments and concerns about this manuscript, which are presented below:

1. The introduction need revision. The necessity of conducting the study and its innovation based on the importance of the topic are not clearly presented.

Author Response: The author thanks the reviewer for this comment. The authors have attempted to resolve these concerns. The necessity for conducting the study goes without saying in that pharmacists and importantly nurses need to have scientific evidence to base their selection of choosing a CSTD on to comply with USP<800>. The innovations were reported previously in 2018 in Plos One and in the 2016 Draft NIOSH universal test protocol in using ATD-GC-MS with a semi-volatile material to mimic and replace the use of hazardous drugs in testing for containment of CSTDs.

2. Depending on the type of study, it is necessary to create a section in the introduction titled Literature Review, review and collect the latest studies conducted in this field, and then highlight the necessity of conducting your research.

Here is a suggested structure and key elements to include in the introduction:

Opening Hook: Begin the introduction with a compelling statement, anecdote, or statistic that captures the reader's attention. This hook should generate interest and establish the relevance of the research topic.

Background and Context: Provide a concise overview of the association between your parameters.

Existing Research Gap: Highlight the gap in the current literature, emphasizing that previous research has predominantly focused on the related parameters.

Research Objective and Aim: Clearly state the objective and aim of the research. Explain that the study aims to fill the research gap by providing a holistic framework.

Core Concerns and Research Questions: Introduce the core concerns and research questions that the paper will address.

Significance and Implications: Discuss the significance and potential impact of the research. Highlight the potential benefits for industry practitioners, policymakers, and the broader society.

Overview of the Paper: Provide a brief overview of the paper's structure, indicating how subsequent sections will address each research question and contribute to the overall objective.

Remember to maintain a clear and concise writing style, using appropriate terminology and avoiding excessive jargon. The introduction should set the stage for the research, establishing its importance and creating anticipation for the subsequent sections of the paper.

3. Add a section titled Study Design at the beginning of the Methods section. In the study design section, please provide comprehensive information, including the study location, methods for determining the sample size, sampling method, inclusion and exclusion criteria, type of study, etc.

4. The discussion section is not written according to scientific principles. In the discussion section, a deep comparison with the results of other occupational studies has not been done. In this section, it is necessary to present a more detailed analysis of the results. For this purpose, you can use the following template:

Here are the key factors to address in a strong discussion:

Summary of Findings: Briefly recap the main findings and results of your research. Provide a concise overview of the key outcomes, trends, or patterns that emerged from your study. Ensure that you mention the most important aspects that contribute to the overall objective of your research.

Reiteration of Research Objective: Restate the objective or purpose of your research to remind the reader of the problem you aimed to address or the knowledge gap you aimed to fill. Emphasize how your findings have successfully addressed the research question or objective stated in the introduction.

Significance and Contribution: Highlight the significance and contribution of your research within the broader context of the field. Explain how your study has advanced knowledge, improved methodologies, or provided valuable insights into the topic. Discuss any implications your findings may have for theory, practice, or policy in the field.

Limitations: Acknowledge the limitations of your study. Address more constraints or challenges that may have affected the research process or the interpretation of results. This demonstrates your awareness of the study's boundaries and adds credibility to your work.

Recommendations for Future Work: Suggest areas for further research or specific directions that future studies could take based on the findings and limitations of your research. Highlight potential avenues for expanding or building upon your work to enhance understanding, address unanswered questions, or improve methodologies.

Conclusion Statement: Conclude your paper by summarizing the overall significance and impact of your research. Restate the main takeaway message or key points you want the reader to remember after reading your paper. Leave a lasting impression by reinforcing the importance of your work and its potential implications for the field.

Avoid Introducing New Information: Ensure that your conclusion does not introduce new data, arguments, or information that has not been discussed in the body of your paper. The conclusion should serve as a synthesis and reflection of the research you have already presented.

Author Response: The author thanks the reviewer and really appreciates the reviewers attempts to provide a very detailed analysis of how to write a scientific paper which has clearly taken some time. The authors believe that they have covered the key sections of a robust scientific publication and present some interesting and important scientific data on containment performance of CSTDs using the latest test protocols from the NIOSH. We have attempted to improve the manuscript and taken onboard the comments and recommendations from six reviewers and so with the greatest of respect to the reviewer on this occasion we are keen to keep the changes within what is achievable at this present time. The work represents a significant body of effort and has been in the review process for a few years and the authors would very much like to move forward to share this data with the scientific community and feel that it achieves the standard required for publication.

Other comment:

Surrogate Selection and Justification: While 2-POE is appropriately chosen for its semi-volatility (better simulating drugs like cyclophosphamide), the manuscript could better explain why 2.5% w/v specifically.

Author Response: The author thanks the reviewer for this comment. The authors have addressed this previously (see above). It is multifactorial but 2.5% w/v is a good concentration to represent clinically useful concentration of drug and also produce detectable concentrations in the vapour which is important when using ATD-GC-MS.

Is this the maximum soluble concentration without saturation, or clinically relevant (e.g., mimicking drug doses)? Compatibility testing with CSTD materials (e.g., no degradation of seals/tubing) is mentioned briefly in responses to reviewers but not in the main text—expand this, as solvents like IPA have known incompatibilities (e.g., per Simplivia white papers). Also, discuss potential limitations: 2-POE's aqueous solubility may not fully represent lipophilic drugs.

Author Response: The author thanks the reviewer for this comment. Again, as with the above the authors have sought to explain this in their responses to reviewers above but feel it sits outside of the scope of the main article. These areas have been covered in responses and submission to the federal docket in 2015 when the NIOSH was asking for input on the design of a new test protocol and the author(s) submitted their comments for this.

The key concept is that 2-POE is not attempting to be all things and represent every hazardous drug, it just has an appropriate chemical vapour concentration that provides a safety factor of at least 100 over the most volatile hazardous drug thiotepa. The work done by the UK Health and Safety Executive (Pengelly and Wheeler et al) is very important in helping inform on surrogate selection and their work is cited and provides the only real-world large scale testing of chemical concentrations of potential surrogates including 2-POE and so was borrowed to inform on the parameters for the NIOSH testing. In terms of the chemical compatibility work, the authors have done some work in this area (unpublished) and others including ICU medical (presented at the town hall meeting at the NIOSH in 2016) demonstrating the benefits of using 2-POE over other potential surrogates in terms of compatibility. There is definitely a need for more of this data to be formally published in a peer reviewed article and I would welcome this for one. 70% Isopropyl alcohol is highly inappropriate as a surrogate on many fronts not least its limited chemical compatibility with a lot of medical grade plastic materials. I think we have covered off most points and the 2-POE at 2.5% w/v does fit with some clinically useful concentrations of drugs but the main driver was not to exceed solubility and to achieve a suitable level of detection in the vapour which was achieved (sub one part per billion volume).

Clarity and Redundancy: The introduction and discussion repeat arguments on surrogate volatility (e.g., IPA vs. 2-POE) across sections, making the manuscript lengthy (as noted by Reviewer #1). Consolidate into a dedicated subsection.

Author Response: The author thanks the reviewer for this comment -most of this now redundant and removed.

Transitions between surrogate rationale and methods could be smoother (e.g., "Based on HSE and NIOSH guidelines, we selected 2.5% w/v 2-POE..."). Abstract lacks explicit mention of replicates (n=4) and statistical methods (e.g., ANOVA)—add for completeness.

Author Response: The author thanks the reviewer for this comment. The authors believe they have addressed concerns about additional statistical analysis of the data – see above. Abstract states n=4 and the author have added in to the abstract the work with replication (n=4) which is then stated in all sections of the article. ANOVA not included as the authors do not think that this approach is appropriate or justified.

Data Availability and Reproducibility: Raw CDS data (Chromeleon v7.3) are not manipulable outside the software, as noted, but provide access instructions or deposit processed data (e.g., peak areas, calibration curves) in a repository like Figshare.

Author Response: The author thanks the reviewer for this comment and was happy to investigate Figshare. Having now looked into this it is not compatible with CDS data from Chromeleon which does not surprise me. The author has addressed the comments on access to RAW data files and has provided a robust defence of why commercial grade CDS software was used for compliance purposes and to enable robust processing of the data without the ability to allow the data to be changed (accidentally) by other operators/ third party. As such the processed data made available was the only accessible data from Chromeleon reporting software. There is also a risk when copying RAW data that it becomes changed or compromised in some way which is why it was only handled within the CDS software environment. All processed data has been made available but the authors will keep looking at options as Figshare is not appropriate and cannot handle the CDS format/ extensions on files. Most cannot handle the MS data sizes.

Supplementary materials (e.g., detailed IFU adherence, chamber diagrams) are referenced but not fully described—ensure they include all protocols for replication.

Author Response: The author thanks the reviewer for this comment. All details are included and IFU was followed as per the manufacturer details. Chamber diagrams are contained in 2015 Draft NIOSH documentation and the revision in subsequent publication made by Wilkinson et al (main author).

Statistical Analysis: Appropriate (e.g., 95% CI for blanks; t-tests for comparisons), but clarify handling of Limitations Not Fully Addressed:

Author Response: The author thanks the reviewer for this comment. Not sure what the reviewer is referring to here in terms of limitation. We were conscious that having reduced the article down from 55 to 30 pages not to reintroduce a lot more supplementary material that was not germain or central to the thrust of the article.

o Chamber volume (125 L) is fixed, but real-world compounding occurs in variable environments (e.g., biological safety cabinets)—discuss generalizability.

Author Response: The author thanks the reviewer for this comment but this sits outside of the scope of the article. We are simply testing according to the NIOSH protocols and as such have to stick with the 125L chamber.

o No assessment of liquid spills or surface contamination; focus is vapor only—note this as a scope limitation.

Author Response: The author thanks the reviewer for this comment – the point of the protocol and use of semi-volatile surrogate is that irrespective of the presentation of the release – whether liquid, vapour or aerosol (which is micro-liquid droplets so liquid again) the surrogate will end up in the vapour and get mixed to a final concentration which is measured in the test. What the protocol does not allow for is discrimination between these different types of physical releases which could be interpreted as a limitation but if the releases are below LLOQ then it is not important. If the releases are large enough to be quantified and give large values then it indicates significant releases have taken place and almost certainly will have some liquid component and this is going to be the main concern in terms of route of exposure.

o Task 2 modification (100 mL bag) is justified, but quantify any impact on results (e.g., vs. standard NIOSH setup).

Author Response: The author thanks the reviewer for this comment - the IV bag is simply a receptacle for the displaced liquid and should be self-explanatory. Have added a change of wording to the methods section to explain. Benefits from no bag mean not making the “push” back into the central IV bag and over-pressurising it or having some additional forces on parts of the system that should not occur during clinical practice. We think the addition of the IV bag make the setup more clinically relevant and hence was included.

o Potential bias: Authors collaborated on the 2016 protocol development—disclose if any CSTD manufacturers influenced selection.

Author Response: The author thanks the reviewer for this comment. The authors are very transparent about their contribution to the 2016 Draft NIOSH universal test protocol but a sigificant input was actually from working with the UK Health and Safety Executive and Ian Pengelly who was credited in the 2018 Plos One publication. Anything involving public sector is done in a none bias way – it has to be that way. I cannot speak for the NIOSH but I would like to think adoption of the 2016 Draft NIOSH universal test protocol was based on its merits scientifically and the fact that it borrows fundamentally from published OSHA and existing OH standards used globally for testing exposure risk in work environments.

Minor Technical Issues:

o Units: Consistently use "ppb" (parts per billion) vs. "ppbv" (parts per billion by volume).

o Figures/Tables: Ensure all (e.g., Figure 3 copyright issue resolved) are high-resolution and labeled.

Author Response: The author thanks the reviewer for this comment – done all instances changed to ppbv and LLOQ.

o Language: Generally clear, but some sentences are dense (e.g., abstract methods)—break for readability.

Author Response: The author thanks the reviewer for this comment – done.

Specific Suggestions for Improvement

• Abstract: Add: "Based on four replicates per task/device, with ANOVA for comparisons..." and briefly justify 2-POE ("a semi-volatile surrogate mimicking hazardous drugs"). Author has added in replication and (n=4) but not referred to ANOVA as we still do not feel this statistical analysis is appropriate for the data type.

Author Response: The author thanks the reviewer for this comment – due to limitations on word count and a belief that the abstract is clear in the objectives, methods and results/ conclusions we will keep this on this occasion but thank you. I don’t think it is easy to briefly explain or justify 2-POE selection and certainly not sufficient to include in abstract. We have attempted this in the main body with references to the work of Pengelly and Wheeler et al at HSE.

• Introduction: Consolidate surrogate discussion (pp. 14-15) and add transition sentence as suggested in reviewer responses.

Author Response: The author thanks the reviewer for this comment – quite a few changes done in this section.

• Methods: Expand on 2-POE concentration (e.g., "2.5% w/v represents maximum aqueous solubility and clinical relevance without material incompatibility"). Confirm IFU adherence explicitly.

Author Response: The author thanks the reviewer for this comment – done “The selection of a surrogate 2-POE concentration of 2.5% w/v has been discussed by the authors previously [21] and represents a clinically relevant concentration of hazardous drug whilst maintaining adequate solubility for the surrogate in aqueous solution. It is also a concentration of surrogate that has been demonstrated to have no chemical incompatibility issues with different plastics employed in various medical devices including CSTDs.”

• Results/Discussion: Include a figure plotting release values (even if • Conclusions: Emphasize implications for USP <800> compliance and future NIOSH updates.

Author Response: The author thanks the reviewer for this comment – not sure what is being suggested as we already have this included in figure 2 for the positive liquid releases of 2-POE versus signal in ppbv and so have decided to keep the main body as it is. Hope this is okay?

• General: Shorten by ~10-15% by removing redundancies; aim for <8,000 words.

Author Response: The author thanks the reviewer for this comment – made a significant reduction in word count as per previous reviewers.

Additional Comments

• No ethical concerns (e.g., no human subjects; devices tested in vitro).

• No plagiarism detected; citations are appropriate (e.g., HSE MDHS 104, NIOSH docket).

• This work could influence policy—suggest authors submit to NIOSH for protocol refinement.

Author Response: The author thanks the reviewer for these comments and input – your input is very much appreciated.

Best Regards.

________________________________________

7. PLOS authors have the option to publish the peer review history of their article (what does this mean?). If published, this will include your full peer review and any attached files.

If you choose “no”, your identity will remain anonymous but your review may still be made public.

Do you want your identity to be public for this peer review? For information about this choice, including consent withdrawal, please see our Privacy Policy.

Reviewer #2: No

Reviewer #3: No

Reviewer #4: No

Reviewer #5: No

Reviewer #6: No

Author Response: The author would like to make all comments and reviews public.

Attachment Submitted filename: Reviewer Responses.docx

# Peer review: Decision Letter 2

**DOI:** 10.1371/journal.pone.0337729.r005

**Authors:** Antonio Peña-Fernández

4 Aug 2026

Dear Dr. Wilkinson,

Thank you for submitting your manuscript to PLOS ONE. After careful consideration, we feel that it has merit but does not fully meet PLOS ONE’s publication criteria as it currently stands. Therefore, we invite you to submit a revised version of the manuscript that addresses the points raised during the review process.

==============================

Thank you for submitting the revised version of your manuscript. It has now been reassessed by the reviewers, who have recommended a number of further revisions. We would be grateful if you could consider their comments carefully and submit a further revised version for our consideration.

==============================

Please submit your revised manuscript by Sep 18 2026 11:59PM. If you will need more time than this to complete your revisions, please reply to this message or contact the journal office at plosone@plos.org. When you're ready to submit your revision, log on to https://www.editorialmanager.com/pone/ and select the 'Submissions Needing Revision' folder to locate your manuscript file.

- A letter that responds to each point raised by the academic editor and reviewer(s). You should upload this letter as a separate file labeled 'Response to Reviewers'.
- A marked-up copy of your manuscript that highlights changes made to the original version. You should upload this as a separate file labeled 'Revised Manuscript with Track Changes'.
- An unmarked version of your revised paper without tracked changes. You should upload this as a separate file labeled 'Manuscript'.

If you would like to make changes to your financial disclosure, please include your updated statement in your cover letter. Guidelines for resubmitting your figure files are available below the reviewer comments at the end of this letter.

If applicable, we recommend that you deposit your laboratory protocols in protocols.io to enhance the reproducibility of your results. Protocols.io assigns your protocol its own identifier (DOI) so that it can be cited independently in the future. For instructions see: https://journals.plos.org/plosone/s/submission-guidelines#loc-laboratory-protocols. Additionally, PLOS ONE offers an option for publishing peer-reviewed Lab Protocol articles, which describe protocols hosted on protocols.io. Read more information on sharing protocols at https://plos.org/protocols?utm_medium=editorial-email&utm_source=authorletters&utm_campaign=protocols.

As the corresponding author, your ORCID iD is verified in the submission system and will appear in the published article. PLOS supports the use of ORCID, and we encourage all coauthors to register for an ORCID iD and use it as well. Please encourage your coauthors to verify their ORCID iD within the submission system before final acceptance, as unverified ORCID iDs will not appear in the published article. *Only*  the individual author can complete the verification step; PLOS staff *cannot*  verify ORCID iDs on behalf of authors.

We look forward to receiving your revised manuscript.

Kind regards,

Antonio Peña-Fernández, PhD

Academic Editor

PLOS One

Journal Requirements:

If the reviewer comments include a recommendation to cite specific previously published works, please review and evaluate these publications to determine whether they are relevant and should be cited. There is no requirement to cite these works unless the editor has indicated otherwise.

Please review your reference list to ensure that it is complete and correct. If you have cited papers that have been retracted, please include the rationale for doing so in the manuscript text, or remove these references and replace them with relevant current references. Any changes to the reference list should be mentioned in the rebuttal letter that accompanies your revised manuscript. If you need to cite a retracted article, indicate the article’s retracted status in the References list and also include a citation and full reference for the retraction notice.

Additional Editor Comments (if provided):

Dear Authors,

Thank you for submitting the revised version of your manuscript. It has now been reassessed by the reviewers, who have recommended a number of further revisions. We would be grateful if you could consider their comments carefully and submit a further revised version for our consideration.

Kind regards,

Antonio

[Note: HTML markup is below. Please do not edit.]

Reviewers' comments:

Reviewer's Responses to Questions

**Comments to the Author**

Reviewer #4: All comments have been addressed

Reviewer #5: All comments have been addressed

Reviewer #6: (No Response)

**********

2. Is the manuscript technically sound, and do the data support the conclusions?

Reviewer #4: Yes

Reviewer #5: Yes

Reviewer #6: (No Response)

**********

3. Has the statistical analysis been performed appropriately and rigorously?

Reviewer #4: Yes

Reviewer #5: Yes

Reviewer #6: (No Response)

**********

4. Have the authors made all data underlying the findings in their manuscript fully available?

The PLOS Data policy

Reviewer #4: Yes

Reviewer #5: Yes

Reviewer #6: (No Response)

**********

5. Is the manuscript presented in an intelligible fashion and written in standard English?

Reviewer #4: Yes

Reviewer #5: Yes

Reviewer #6: (No Response)

**********

Reviewer #4: (No Response)

Reviewer #5: Detailed Comments by Section

• Abstract

1. Strengths: Clearly states objectives, methods, and key findings. The inclusion of replication (n=4) and the use of positive/negative controls is appropriate.

2. Areas for Improvement:

- Briefly state why 2-POE was chosen as the surrogate (e.g., "a semi-volatile surrogate mimicking hazardous drugs")—this would reinforce methodological rigor.

- The abstract is somewhat lengthy (448 words). Consider condensing while maintaining key details.

3. Recommendation: Add one sentence on the rationale for 2-POE selection (semi-volatile properties, relevance to hazardous drugs).

• Introduction

1. Strengths: Establishes the importance of CSTDs and the evolution of NIOSH protocols. References to USP 800 and regulatory requirements are appropriate.

2. Minor Concerns:

- Excessive length and repetition: The introduction is disproportionately long and repeats points about surrogate volatility, criticisms of the 2015 NIOSH protocol, and the rationale for 2-POE selection across multiple paragraphs. This should be condensed significantly.

- Lack of focus: The narrative provides extensive background before narrowing to the specific research question. The research gap and study objectives should be foregrounded more directly.

- Literature review: While referencing is comprehensive, there is no structured literature review section summarizing the latest studies in this field and clearly articulating the necessity of the present study.

3. Recommended Revisions:

- Consolidate the surrogate selection discussion into a dedicated, concise subsection.

- Reduce repetition regarding IPA vs. 2-POE volatility arguments.

- Add a transition sentence such as: "Based on HSE and NIOSH guidelines, we selected 2.5% w/v 2-POE as the challenge agent for this study."

- Explicitly state the null hypothesis in the introduction (authors added this in revision but could be clearer).

• Methods

1. Strengths:

- Adherence to the 2016 Draft NIOSH universal test protocol and manufacturer IFUs ensures consistency and validity.

- Use of ATD-GC-MS with SIM detection is the gold standard for volatile/semi-volatile analysis.

- Appropriate controls (positive controls at 5 and 10 µL, negative controls with Milli-Q water, chamber blanks).

- The addition of a 100 mL empty IV bag in Task 2 to avoid pressurization is a practical improvement.

2. Minor Concerns:

- Sample size justification: The use of n=4 replicates is based solely on the NIOSH protocol without statistical power calculation or justification. Given that all results were below LLOQ, the authors should justify why n=4 is sufficient and whether a power analysis was considered.

- Methodological details: Key parameters (ATD-GC-MS conditions, column temperatures, desorption times, gas flow rates) are in the supplementary materials. Essential parameters should be summarized in the main Methods section for reproducibility without needing to consult supplementary files.

- Data availability: Raw instrument data (Chromeleon files) are not accessible, which violates PLOS ONE's data policy. The authors state that proprietary software and large file sizes prevent sharing, but they should provide processed data (e.g., peak areas, calibration curves) in a usable format (e.g., CSV, Excel) deposited in a public repository like Figshare.

- "No residue" verification: The statement "No carryover of 2-POE was observed in the chamber" requires clarification. How was this verified? Air sampling? Visual inspection? The authors should specify the method (e.g., "confirmed by chamber blank air samples showing 2-POE concentrations below LOD").

3. Minor Concerns:

- The statement that 2.5% w/v 2-POE has "no chemical incompatibility issues" with CSTD plastics is mentioned but not supported by a reference. A citation or explanation of compatibility testing would strengthen the methods.

- Units: Use "ppbv" consistently throughout (some instances of "ppb" without specifying volume).

4. Recommended Revisions:

- Add a brief justification for n=4 (e.g., "as specified in the 2016 Draft NIOSH protocol, and consistent with previous studies to enable comparison").

- Summarize key ATD-GC-MS parameters in the main text, with full details in supplementary.

- Clarify the residue verification method.

- Address data availability concerns.

• Results

1. Strengths:

- Data presentation in Table 2 (now Table 4 in revised version) is clear, showing mean concentrations, 95% CIs, and LLOQ.

- Positive control results (3.13 ppbv and 6.59 ppbv for 5 and 10 µL releases) demonstrate system suitability.

- Negative controls show values indistinguishable from background, confirming no contamination.

- The ability to quantify down to ~650 nL liquid release is a notable technical achievement.

2. Areas for Improvement:

- Since all results are below LLOQ, the statement "no statistically significant difference was found" is technically correct but requires clarification. The authors should explicitly state that formal statistical testing (e.g., ANOVA) was not performed because all values were below the limit of quantification, making such comparisons inappropriate.

- The authors state the LLOQ in the present study (0.41 ppbv) is half the previous value (0.88 ppbv). This improvement should be emphasized more clearly.

- Some figures (e.g., Figure 3 showing the NIOSH chamber) are not clearly referenced in the text—ensure all figures are cited in order of appearance.

3. Recommended Revisions:

- Clarify that statistical comparison was not performed due to all data being LLOQ, and that the conclusion of "no difference" is based on all devices being below the same LLOQ threshold.

- Add a figure or mention in text showing the proportional response between liquid release volume and 2-POE signal (Figure 2 effectively illustrates this).

• Discussion and Conclusion

1. Strengths:

- Appropriately highlights the significance of finding parity between air-cleaning and barrier CSTDs.

- Discusses the advantages of using semi-volatile surrogates over volatile ones (IPA) with reference to Pengelly et al.'s work.

- Emphasizes the importance of standardized testing methods.

2. Minor Concerns:

- Overemphasis on protocol defense: A significant portion of the discussion is devoted to defending the 2016 NIOSH protocol, criticizing the 2015 protocol, and responding to other researchers' approaches (Doepke, Westbrook). This detracts from interpreting the study's own findings.

- Limited discussion of practical significance: The clinical/occupational health implications are underdeveloped. What does "below LLOQ" mean in terms of real-world exposure risk? How should healthcare organizations interpret these findings when selecting a CSTD? The authors should address these questions more directly.

- No dedicated Limitations section: Important limitations are not systematically addressed:

- Use of a single surrogate concentration (2.5% w/v)

- Absence of real-world validation (the study is laboratory-based)

- Small number of CSTD models tested (only 4)

- Lack of exposure-risk correlation

- Potential healthy worker effect (not applicable here, but generalizability)

- The 2016 NIOSH protocol remains a draft, and there is no pass/fail criterion

- Discussion depth: The discussion is somewhat superficial in comparing findings with existing literature. A deeper comparison with other CSTD studies would strengthen the paper.

3. Minor Concerns:

- The conclusion is lengthy and reiterates points from the discussion. Condense to a clear summary of findings, implications, and future research.

- The funding statement and potential conflicts of interest should be consolidated into a single Financial Disclosure section (already addressed in revision).

4. Recommended Revisions:

- Shift discussion focus from protocol defense to interpretation of results and practical implications.

- Add a structured Limitations section covering the points mentioned above.

- Condense the conclusion to a concise summary (3-4 paragraphs maximum).

- Discuss the implications for USP 800 compliance more explicitly.

• References

1. Strengths:

- Comprehensive coverage of key regulatory documents (NIOSH, USP 800, HSE).

- Relevant methodological references (ISO standards, OSHA methods).

- Appropriate citation of prior CSTD studies.

2. Areas for Improvement:

- Some references are "private communication" or presentations (Refs. 30–33). Replace with formally published sources where possible, or clearly state why formal citation is not possible.

- The chemical compatibility claim (2-POE with medical plastics) lacks a supporting reference. Add a citation.

- Some references have formatting inconsistencies (e.g., some have DOIs, others not). Ensure all references are complete and consistently formatted according to PLOS ONE style.

- The reference numbering appears to have duplicates or inconsistencies in the revised version (e.g., Refs. 24 and 25, 26 and 27 appear to duplicate content). Carefully check and correct.

3. Recommended Revisions:

- Verify all references for completeness, accuracy, and consistency.

- Add a reference for the chemical compatibility claim.

- Replace "private communication" citations with published sources where feasible.

Final Recommendation for Authors

Recommendation: Minor Revision

This manuscript addresses an important and timely topic: the containment performance of closed system transfer devices (CSTDs) used to protect healthcare workers from hazardous drug exposure. The study employs a rigorous methodology based on the 2016 Draft NIOSH universal test protocol, uses appropriate analytical techniques (ATD-GC-MS), and provides valuable comparative data on both physical barrier and air-cleaning CSTDs. The finding that all four tested devices performed below the limit of quantification (LLOQ) is significant for healthcare organizations making purchasing decisions and complying with USP 800.

However, the manuscript has several significant limitations that require attention before publication:

Must Address:

1. Excessive Length and Repetition: The manuscript is far too long (120 pages in the original version, reduced to ~30 pages in the revised version but still lengthy). The introduction and discussion contain extensive repetition, particularly regarding surrogate selection rationale, criticisms of the 2015 NIOSH protocol, and defense of the 2016 protocol. These sections should be condensed and focused.

2. Focus on Protocol Defense Rather Than Results: A large portion of the discussion is devoted to defending the 2016 NIOSH protocol and critiquing other methods. While context is important, the focus should shift toward interpreting the results of this study and their implications for CSTD selection and healthcare worker safety.

3. Lack of Dedicated Limitations Section: Important limitations are not systematically addressed, including:

- Use of a single surrogate concentration (2.5% w/v) and one surrogate material

- Absence of real-world validation (laboratory-only study)

- Small number of CSTD models tested (only 4 devices, each from a limited set)

- Lack of correlation between surrogate release and actual hazardous drug exposure risk

- The 2016 NIOSH protocol remains a draft with no established pass/fail criterion

- The use of n=4 replicates based on the protocol without statistical justification

4. Underdeveloped Practical Implications: The discussion of what "below LLOQ" means in terms of real-world exposure risk for healthcare workers is insufficient. How should end-users interpret these results when selecting a CSTD? The authors should address this more directly.

5. Data Availability: Raw instrument data (Chromeleon files) are not accessible. The authors should provide processed data (e.g., peak areas, calibration curves) in a usable format deposited in a public repository to comply with PLOS ONE's data policy.

6. Statistical Reporting: The statement "no statistically significant difference was found" is used, but no specific statistical test, p-value, or effect size is reported. Since all values are below LLOQ, the authors should clearly state that formal statistical testing (e.g., ANOVA) was not performed because all measurements were below the limit of quantification, making such comparisons inappropriate. Then explain why the conclusion of "no difference" is valid.

7. Conflict of Interest Transparency: The study was funded by ICU Medical, which manufactures two of the four tested devices. Although the authors state the funder had no role in study design or analysis, this potential bias should be more transparently discussed in the Limitations section.

Recommended Revisions:

1. Condense the introduction and discussion by removing repetitive sections, particularly those focused on surrogate selection debates and protocol critiques. Move detailed historical context to supplementary materials if necessary.

2. Add a structured Limitations section comprehensively addressing the points mentioned above.

3. Strengthen the discussion of practical implications:

- What does "below LLOQ" mean in terms of healthcare worker exposure risk?

- How should healthcare organizations interpret these findings when selecting a CSTD?

- What are the implications for USP 800 compliance?

4. Clarify statistical reporting:

- Explicitly state that formal statistical testing was not performed because all values were LLOQ.

- Explain why the conclusion of equivalent performance is valid (all devices below the same LLOQ threshold).

5. Address data availability:

- Deposit processed data (e.g., peak areas, calibration curves) in a public repository.

- Provide instructions on how to access the data.

6. Summarize key ATD-GC-MS parameters in the main Methods section (not just in supplementary materials).

7. Clarify the "no residue" verification method (how was no carryover confirmed?).

8. Correct reference numbering and ensure all references are complete and consistently formatted.

Strengths to Maintain:

- Rigorous methodology based on the 2016 Draft NIOSH universal test protocol

- Use of ATD-GC-MS with SIM detection—the gold standard for volatile/semi-volatile analysis

- Appropriate controls (positive, negative, blanks)

- Clear and well-organized data presentation (tables and figures)

- Transparent disclosure of funding and potential conflicts of interest

- Important practical implications for healthcare worker safety

Overall Assessment:

The study makes a useful contribution to the field of CSTD containment performance evaluation, and the findings are valuable for healthcare organizations. However, the manuscript requires Minor revision to address the issues of length, focus, practical implications, limitations, and data availability. With these revisions, the manuscript would be suitable for publication in PLOS ONE and would make a meaningful contribution to the occupational health literature on protecting healthcare workers from hazardous drug exposure.

Reviewer #6: Ref.: PONE-D-25-12716R2

Title: Head-to-head containment performance testing of four commercially available closed-system transfer devices using the 2016 Draft NIOSH test protocol and 2.5% w/v 2-phenoxyethanol as challenge agent

Dear Editor,

Thank you for the opportunity to review this revised manuscript.

This study presents a technically sound and well-designed comparison of four commercially available closed-system transfer devices (CSTDs) using the 2016 Draft NIOSH universal test protocol with 2.5% (w/v) 2-phenoxyethanol (2-POE) as the surrogate challenge agent. The analytical methodology is robust, the experimental procedures are carefully controlled, and the manuscript addresses an important question in occupational health and pharmacy practice regarding the comparative containment performance of barrier and air-cleaning CSTD technologies.

The authors have addressed many of the concerns raised during previous rounds of review, and the manuscript has improved substantially. Nevertheless, several issues remain that should be addressed before publication.

Major Comments

1. Manuscript Organization and Redundancy

Although the revised version is improved, portions of the Introduction and Discussion remain unnecessarily detailed and repetitive, particularly regarding the historical development of the NIOSH protocols and the justification for selecting 2-POE as the surrogate.

The manuscript would benefit from further condensation, allowing greater emphasis on the present findings rather than revisiting previously published methodological debates. Discussions regarding earlier NIOSH protocols may be shortened or moved to supplementary material.

2. Interpretation of Results Below the LLOQ

All reported measurements were below the lower limit of quantification (LLOQ), which represents an important characteristic of the dataset.

The manuscript should explicitly state that formal inferential statistical comparisons were not performed because all measured concentrations were below the LLOQ. Consequently, the conclusion that barrier and air-cleaning CSTDs demonstrated equivalent containment performance should be interpreted as a descriptive observation based on the available analytical sensitivity rather than as statistical evidence of equivalence.

Furthermore, the Discussion should acknowledge that the absence of measurable differences does not necessarily demonstrate identical performance between devices; rather, it indicates that any potential differences were below the detection capability of the analytical method employed.

3. Data Availability

The current Data Availability Statement could be strengthened.

Although proprietary Chromeleon software may limit sharing of raw instrument files, the processed analytical data (e.g., calibration data, calculated concentrations, instrumental responses, and summary datasets) could be deposited in an openly accessible repository such as Figshare, Zenodo, or Dryad.

Providing these processed datasets would substantially improve reproducibility while remaining fully compatible with the PLOS ONE Data Availability Policy.

4. Practical Interpretation of Findings

The Discussion would benefit from a clearer explanation of the practical implications of reporting concentrations below the LLOQ.

Specifically, the manuscript should help readers understand what "<lloq" and="" clinical="" containment="" cstd="" drug="" findings="" for="" handling.="" hazardous="" how="" in="" influence="" interpreting="" performance="" practice="" routine="" selection="" should="" signifies="" these="" when="">Even if direct exposure-risk estimation is beyond the scope of the study, a concise discussion of the practical meaning of these results would considerably strengthen the manuscript.

5. Study Limitations

A dedicated Limitations section should explicitly acknowledge several important constraints, including:

• the inability to distinguish performance differences when all measurements fall below the analytical quantification limit;

• evaluation of only four commercially available devices;

• reliance on a surrogate rather than hazardous drugs;

• absence of correlation between surrogate release measurements and real-world occupational exposure.

Providing these limitations would improve transparency and assist readers in interpreting the study appropriately.

Minor Comments

1. The Abstract could be slightly condensed and briefly indicate the rationale for selecting 2-POE as the surrogate.

2. The manuscript should consistently use the terminology LLOQ throughout the text instead of alternating between LOQ and LLOQ.

3. Units should be standardized to ppbv throughout the manuscript.

4. The authors should ensure that all figures and tables are cited sequentially within the text.

5. The rationale for adopting n = 4 replicates (as specified in the Draft NIOSH protocol) could be briefly stated in the Methods section for completeness.

6. The confidence intervals presented for measurements below the LLOQ should be clearly described as reflecting instrumental responses rather than quantified concentrations.

7. Several references should be reviewed to ensure complete bibliographic information and DOI formatting where available.</lloq">

**********

what does this mean?). If published, this will include your full peer review and any attached files.

If you choose “no”, your identity will remain anonymous but your review may still be made public.

**Do you want your identity to be public for this peer review?**  For information about this choice, including consent withdrawal, please see our Privacy Policy

Reviewer #4: No

Reviewer #5: No

Reviewer #6: No

**********

[NOTE: If reviewer comments were submitted as an attachment file, they will be attached to this email and accessible via the submission site. Please log into your account, locate the manuscript record, and check for the action link "View Attachments". If this link does not appear, there are no attachment files.]

To ensure your figures meet our technical requirements, please review our figure guidelines: https://journals.plos.org/plosone/s/figures

You may also use PLOS’s free figure tool, NAAS, to help you prepare publication quality figures: https://journals.plos.org/plosone/s/figures#loc-tools-for-figure-preparation.

NAAS will assess whether your figures meet our technical requirements by comparing each figure against our figure specifications.

# Author comment: Author response to Decision Letter 3

**DOI:** 10.1371/journal.pone.0337729.r006

18 Sep 2026

PONE-D-25-12716R1

Head-to-head containment performance testing of four commercially available closed-system transfer devices using the 2016 Draft NIOSH test protocol and 2.5% w/v 2-phenoxyethanol as challenge agent.

Dear PLOS One

Please find below the authors rebuttal responses to reviewers questions for the above manuscript.

Please find included the following items and files:

• A letter that responds to each point raised by the academic editor and reviewer(s). This letter is a separate file labelled 'Response to Reviewers'.

• A marked-up copy of the latest manuscript that highlights changes made to the original version. This file is labelled 'Revised Manuscript with Track Changes'.

• An unmarked version of the revised paper without tracked changes. This is a separate file labelled 'Manuscript'.

Authors Responses to Reviewers Questions:

Reviewer’s Comments – downloaded from portal on Plosone.

OVERALL FIRST IMPRESSIONS

Tone & Audience Fit: The tone is appropriately academic and aligns well with the intended audience—healthcare professionals, pharmacists, occupational safety experts, and researchers working in oncology and hazardous drug handling. The language is formal but not unnecessarily complex, which is suitable for a journal like PLOS ONE.

ASW Author Response: The authors thank the reviewer for this input. The authors have gone to significant lengths now over several reviews to refine the manuscript and appreciate the comments.

Opening Hook: The abstract begins effectively by stating the objective clearly: assessing the containment performance of four CSTDs using the 2016 Draft NIOSH protocol and 2-POE as a surrogate. However, the introduction could benefit from a more engaging opening sentence or paragraph that emphasizes the real-world stakes of inadequate CSTD performance (e.g., occupational exposure risks).

ASW Author Response: The authors thank the reviewer for this input. The authors have included the following paragraph to improve the emphasis on inadequate CSTD performance. – see below in Introduction – Background first page.

“A CSTD that does not demonstrate high levels of containment that could result in accidental releases of hazardous drugs may offer no improvement over use of an “open system” of needle and syringe or could lead to higher exposure of healthworkers over that of using an “open system”. This is why the science of CSTD testing and the use of robust validated scientific methods is vital for making informed decisions when purchasing a CSTD technology.”

ABSTRACT REVIEW

Strengths:

• Clear statement of objectives.

• Concise description of methodology.

• Summary of key findings is well articulated.

• Conclusions are directly tied to the study’s purpose.

ASW Author Response: The authors thank the reviewer for this input.

Weaknesses:

• Missing context on why 2.5% w/v 2-phenoxyethanol was selected: This should be briefly explained in the abstract, especially since it's central to the test method.

ASW Author Response: The authors thank the reviewer for this input but the abstract is already at the maximum word count and the authors have found it impossible to provide context in the abstract but this is more than covered in the main manuscript which the authors believe is the correct place for this discussion which is too complex to be covered in just a few words.

• Statistical significance vs. practical relevance: While statistical differences were noted between some CSTD types, the abstract does not clarify what those differences mean in practice (e.g., magnitude of exposure risk).

ASW Author Response: The authors thank the reviewer for this input. Again, the authors believe that the abstract is not the correct place and due to word count constraints cannot support a discussion on this topic. Also, the review appears to misunderstand the key findings – there was no difference observed in performance between the different CSTD tested in the study. In the main discussion the authors do discuss what the impact would be for healthworkers using a poor performance CSTD – higher exposure risk potentially greater than using an “open system” of needle and syringe.

• Lack of mention of sample size or replicates: Readers may want to know how many trials were conducted per device.

ASW Author Response: The authors thank the reviewer for this input. The number of replicates is clearly stated as n=4. This is consistent throughout the abstract and main body of the manuscript.

Suggested Improvements:

“This study evaluated the containment performance of four commercially available closed-system transfer devices (CSTDs) using the 2016 Draft NIOSH universal test protocol and 2.5% w/v 2-phenoxyethanol (2-POE), a semi-volatile surrogate selected for its representative behaviour compared to hazardous drugs.”

ASW Author Response: The authors thank the reviewer for this suggestion but have not been able to incorporate the suggested wording without exceeding the 500 word count for the abstract.

Add: “Results are based on [X] replicate tests per device, with statistical comparisons made using [method].”

ASW Author Response: The authors thank the reviewer for this input. The authors believe that they have included all relevant information for replication. No statistical comparison is made between CSTDs only that they all demonstrated releases that were below LLOQ (not quantifiable).

INTRODUCTION

Flow & Logical Development:

The introduction builds up to the research question logically, starting with background on hazardous drugs, moving into CSTD classifications, then discussing prior protocols leading to the 2016 NIOSH draft.

Strengths:

• Solid foundation on the importance of CSTDs.

• Good explanation of the limitations of earlier testing methods.

• Clear rationale for adopting the 2016 NIOSH protocol.

ASW Author Response: The authors thank the reviewer for this input.

Areas for Improvement:

• Repetition of content: Some paragraphs repeat similar points about IPA volatility and surrogate selection across different sections. Consolidate these ideas for better flow.

• Need for clearer transition: Between the discussion of surrogate selection and the actual methodology used in this study. A sentence such as “Given these considerations, we selected 2-POE at a concentration of 2.5% w/v…” would help bridge the gap.

ASW Author Response: The authors thank the reviewer for this input.

Suggested Edit:

“While IPA has been widely used historically, its high volatility makes it unsuitable for accurately simulating the vapor release characteristics of most hazardous drugs. In response, the 2016 Draft NIOSH protocol proposed the use of semi-volatile surrogates, including 2- phenoxyethanol (2-POE). Based on these guidelines, we selected 2.5% w/v 2-POE as the challenge agent for our comparative assessment of four CSTDs.”

ASW Author Response: The authors thank the reviewer for this input and the above statement has now been incorporated into the introduction section. Thank you.

METHODS SECTION

Overall Assessment:

The methods section is generally thorough and follows the 2016 Draft NIOSH protocol closely. However, several areas require clarification or expansion to meet the standards expected in a peer-reviewed publication.

Key Issues:

1. Surrogate Selection Justification

• What led to the choice of 2.5% w/v? Is this concentration within the range recommended by NIOSH or derived empirically?

• Was there any pre-study compatibility testing with the tested CSTD materials? As noted in the literature (e.g., Simplivia white paper), some solvents can degrade tubing or seals.

ASW Author Response: The authors thank the reviewer for this input. The concentration was selected based on the maximum achievable concentration without exceeding the solubility limit. Yes there was pre-study compatibility testing performed both by ICU medical (published in the town hall meeting at NIOSH in 2016) and by the authors (in-house and not published or citable). The below text has now been added to the manuscript in the methods section.

“The selection of 2.5% w/v 2-POE was made so as to maximise the available signal from releases of 2-POE in chamber and hence increase sensitivity in the assay without exceeding the solubility limit for 2-POE.”

“Compatibility between aqueous solutions of 2-POE and the medical devices was assessed prior to test (in-house data) with no observable changes in performance in any of the four CSTDs evaluated in the study following extended contact with the surrogate 2-POE at a concentration of 2.5% w/v.

2. Device Testing Protocol

• Were all manipulations performed strictly according to IFUs? If so, specify whether deviations were recorded or monitored.

• Were negative controls included? For example, baseline chamber contamination levels before each test.

ASW Author Response: The authors thank the reviewer for this input. The CSTDs were all operated in strict accordance with the IFUs and no deviations were recorded. The operators conducting the tests used recording sheets to record contemporaneous notes and observations.

Negative controls as described above were conducted by the operators prior to each test. Both the laboratory background was observed on each day of test to record the amount of 2-POE occurring naturally in the background make up air and chamber blanks were performed in the chamber loaded with all of the test equipment prior to test to ensure that there was no 2-POE release prior to test. This is overt in the body of the manuscript and Methods section – see bullet point number 4 below.

“Dual blank chamber measurements were recorded prior to testing of a CSTD to ensure no contamination inside the chamber.”

3. Analytical Methods

• Clarify ATD-GC-MS parameters: What were the temperature settings, desorption times, column type, etc.? Without this detail, reproducibility is compromised.

ASW Author Response: The authors thank the reviewer for this input. The authors have now transposed those details from supplementary materials (S1 document) to the Methods section under “Sample analysis”. Specifically the following additional operating parameters and details have been included in the methods section of the manuscript.

“Stainless steel air sampling tubes (thermal desorption [TD tubes) were used with physical dimensions (89 mm (3.5”) long × 6.4 mm (¼”) outer diameter (OD) packed with 200 mg of Tenax TA sorbent, retained between two steel gauzes (Markes, UK). A TD tube conditioner (Markes TC-20, UK) was used to prepare tubes. After conditioning, the tubes were cooled to room temperature and capped with the metal end caps. The sampling tubes were used and immediately prior to analysis spiked with an accurately known mass of d8-toluene according to a 1mL addition from a 1 parts per million (ppm) standard prepared in four nines di-nitrogen balance gas (BOC, UK). Sampling pumps (Gil-Air, UK) certified to BS EN ISO 13137 and capable of maintaining a constant flow rate of 100 mL/min ± 5% were used. A calibrated flow meter with a measurement uncertainty of ± 2% was used to calibrate the flow rate of the air sampling pump prior to each use. Immediately prior to running a test sample, two chamber blank air samples were collected under conditions identical to the test samples. The blank samples were then used to calculate the experimental limit of detection (LOD) and lower limit of quantitation (LLOQ). A single negative control test was always performed for each closed system transfer device (CSTD) tested for both NIOSH task 1 and task 2. This test was performed following the same procedure as for the CSTD sample tests but using drug vials containing 100% deionized (Milli-Q) water rather than the 2.5% w/v POE challenge agent.

Determination of breakthrough volume and recovery on TD tubes:

Breakthrough tests with sample volumes of 3, 6, and 12 liters were performed to verify that no breakthrough and >99.9% recovery from the Tenax TA occurred. Eight test tubes were pre-spiked with internal standard and 5 µl of standard 1, giving tube loading of ~180 ng of 2-POE and approximately 100 ng of d8-toluene. Two tubes were analyzed by TD-GC-MS immediately and then re-analyzed for traces of residual 2-POE in the second analysis. In between the first and second analysis, a clean blank tube was analyzed to demonstrate no carryover of 2-POE within the instrument and confirmed the trap desorption efficiency. The remaining six tubes were connected to a backup tube, loaded with internal standard but free of 2-POE. Dry air at 100 mL/min was pumped through the tubes for 30 (tubes 3 and 4), 60 (tubes 5 and 6), or 120 minutes (tubes 7 and 8), giving air sampling volumes of 3, 6, and 12-liters, respectively. At the end of the test, all twelve tubes were analyzed by TD-GC-MS and the results were used to confirm the system performance and demonstrate absence of breakthrough for 2-POE.

Instrument Settings:

An automated Thermo Scientific Trace 1300 gas chromatograph fitted with a TD100-xr thermal desorption autosampler (Markes, UK) interfaced to a Thermo Scientific ISQ 7000 AEI mass spectrometer, a VOCOL capillary column (60 m length × 0.25 mm diameter × 1 µm film thickness), and data was acquired and processed using the CDS software supplied by Thermo Scientific (Chromeleon version 7.3). The automated thermal desorption (ATD) conditions were as follows: valve temperature 225°C, desorb temperature 300°C, purge time 1 minute, desorption time 10 minutes, desorption flow rate 70 mL/min, trap temperature (low) −30°C, trap temperature (high) 270°C, outlet split flow 30 mL/min, column flow 1.3 mL/min, transfer line temperature 200°C. GC conditions were as follows: initial oven temperature 100°C (hold for 5 minutes), temperature ramp 1: 5°C/min (to 150°C; hold for 0 minutes), temperature ramp 2: 10°C/min (to 200°C; hold for 10 minutes). Under these conditions the retention times of internal standard d8-toluene and 2-POE were around 9.79 and 22.97 minutes, respectively. The MS conditions were as follows: multiplier voltage = 350 volts, total ion mass (low) = 25, total ion mass (high) = 200, selected ion masses for detection of d8-toluene = 95 – 105 m/z, selected ion masses for detection of 2-POE = 77, 94, and 138 m/z.”

• Calibration curves: Were they generated fresh for each batch of samples? How was linearity confirmed?

ASW Author Response: The authors thank the reviewer for this input. The instrument was calibrated against a full set of standards for 2-POE on each day of test and the calibration curve data used to interpret the amounts of 2-POE found on tube for each day of test. Linear Regression analysis was performed inside the Chromeleon ver7.3 CDS software and produced a line of best fit with appropriate R2 value >0.99.

4. Sample Size and Replication

• How many replicates were run per device? This is essential for interpreting statistical confidence in results.

• Were outliers excluded? If yes, under what criteria?

ASW Author Response: The authors thank the reviewer for this input. The authors have now added the following text in the methods section.

“Each CSTD device test replicate was performed using duplicate TD tubes in each test and the test was performed with replication (n=4) providing in total 8 data points for each NIOSH task. No data points were assessed as being outliers or excluded in the analysis.”

5. Statistical Methods

• Which statistical tests were used? T-tests, ANOVA, non-parametric equivalents?

• Was multiple comparison correction applied? Given the number of pairwise comparisons, this should be addressed.

• Define what constitutes a statistically significant difference (e.g., p < 0.05).

ASW Author Response: The authors thank the reviewer for this input. The authors have on previous rounds of review for this manuscript rebutted this same question regarding the application of sophisticated statistical treatment to the data. No tests were deemed appropriate given that the data was below LLOQ and so not quantifiable. The only statistical methodology used was the linear regression analysis performed within Chromeleon CDS software for analysis of the peak area data and conversion in to an amount of 2-POE in nanograms. The statement below has now been added in the methods section of the manuscript.

“The only statistical methodology used was the linear regression analysis performed within Chromeleon v7.3 CDS software for analysis of the peak area data and conversion in to an amount of 2-POE in nanograms. As all data obtained for the CSTDS was below LLOQ no other statistical data treatments were deemed appropriate.”

• Was multiple comparison correction applied? Given the number of pairwise comparisons, this should be addressed.

• Define what constitutes a statistically significant difference (e.g., p < 0.05).

As stated above no pairwise comparisons were made as all data was below LLOQ. These tests would be meaningless. The author has reviewed the manuscript and removed the word “statistical” from any description of comparisons in the data to avoid further confusion.

Suggested Additions:

“All testing procedures were performed strictly in accordance with manufacturer instructions for use (IFU) to ensure validity. Deviations, if any, were documented and reviewed for potential impact on containment integrity.”

ASW Author Response: The authors thank the reviewer for this input and this statement has been added to the methods section. Thank you.

“To assess repeatability, five replicate tests were conducted for each CSTD model. Outliers were identified using the Grubbs’ test and removed only when statistically justified.”

ASW Author Response: The authors thank the reviewer for this input. No action necessary. No data was removed,

“Chromatographic separation was achieved using a DB-5MS capillary column (30 m × 0.25 mm × 0.25 μm; Agilent Technologies) with helium as the carrier gas at a constant flow rate of 1.2 mL/min. Calibration was verified daily using freshly prepared standards.”

ASW Author Response: The authors thank the reviewer for this input. The additional detail has now been added in full.

RESULTS SECTION

Strengths:

• Clear presentation of data.

• Tables summarize findings effectively.

• Statistical outcomes are reported where applicable.

ASW Author Response: The authors thank the reviewer for this input.

Areas for Clarification:

• Figure/Table Correspondence: Ensure every figure and table is explicitly referenced in the text and that their captions are self-explanatory.

ASW Author Response: The authors thank the reviewer for this input. This has been addressed previously by the authors and checked again for accuracy.

• Units and precision: Make sure units (e.g., ppbv) are consistent throughout and that decimal places reflect measurement precision.

ASW Author Response: The authors thank the reviewer for this input. This has been addressed previously by the authors and checked again for accuracy.

Example:

“As shown in Table 2, ChemoLock™ barrier demonstrated significantly lower vapour leakage than Tevadaptor™ air-cleaning (p = 0.008).”

ASW Author Response: The authors thank the reviewer for this input. I am not sure what this refers to as we never applied the p-test to our data, and we were unable to make such discrimination based on our data. I am concerned that the reviewer is referring to something that does not exist in our manuscript – or am I missing the point here. If the point is measurement precision, then I can assure the reviewer that we have addressed measurement precision in a very early edit of the manuscript and have again checked this detail. No action needed.

DISCUSSION

Strengths:

• Well-grounded in previous literature.

• Interpretation of results aligns with known properties of CSTD technologies.

• Acknowledges limitations of the current methodology.

ASW Author Response: The authors thank the reviewer for this input.

Weaknesses:

• Overemphasis on protocol validation: While important, the discussion should center more on the implications of the observed differences among the CSTDs.

• Limited interpretation of practical significance: Beyond statistical significance, what do the differences mean for healthcare worker exposure? Are the measured concentrations below regulatory thresholds?

ASW Author Response: The authors thank the reviewer for this input. The implications on the differences between the four CSTD assessed in the study are none because they all performed to an equivalent standard (below LLOQ). The protocol validation is important as with all of the validations and is what distinguishes the present work with other studies that did not apply robust methodology. The authors are trying to engage the reader with why these important analytical concepts need to be adhered to.

There are no regulatory thresholds for healthworker exposure to hazardous drugs and this is unfortunate when it comes to trying to persuade institutions to use better protection equipment such as CSTDs within their organisations. The authors have revisited this and attempted to highlight the potential dangers from the high positive control releases of 2-POE and what they might mean for hazardous drug releases in clinical practice and how this is avoided by adoption of any one of the four CSTDs assessed in the NIOSH tasks.

The following text has been added to the discussion section of the manuscript.

“It is not possible to draw any correlation between the results obtained using 2-POE as surrogate and healthcare worker exposure to hazardous drugs when handled using the four CSTD evaluated in this study. However, all four CSTDs contained the 2-POE which is a semi-volatile material and thus behaves in an analogous chemical manner to many hazardous drug materials to below LLOQ values. In contrast, when an “open-system” such as needle and syringe was used for positive control test performed significant vapour concentrations of 2-POE were detected at 3.13 ppbv and 6.59 ppbv respectively for release volumes of 5 and 10 microlitres of liquid surrogate solution. Whenever there are liquid releases of hazardous drugs during preparation or administration there is an increased likelihood of healthcare worker exposure by direct or secondary contact with the liquid. None of the four CSTDs assessed in the study produced observable liquid releases and the signal for 2-POE was significantly below those values associated with positive liquid releases of 2-POE and were below quantifiable limits (<LLOQ).”

Suggested Revisions:

“The observed differences in vapour containment among the tested CSTDs suggest that not all systems offer equivalent protection against occupational exposure. These findings support the need for standardised, performance-based evaluation methods such as the 2016 Draft NIOSH protocol.”

“Further research is needed to correlate laboratory-measured vapour leakage with real-world exposure scenarios to determine whether these differences translate into clinically meaningful reductions in risk.”

The following statement has been added to the end of the discussion section – the authors wish to thank the review for this helpful suggestion.

“Further research is needed to correlate laboratory-measured vapour leakage with real-world exposure scenarios to determine whether these differences translate into clinically meaningful reductions in risk.”

CONCLUSION

Strengths:

• Directly addresses the research objective.

• Succinct and clear.

ASW Author Response: The authors thank the reviewer for this input.

Suggestion:

Consider rephrasing the conclusion to emphasise the broader implication:

“These findings underscore the importance of adopting standardised, semi-volatile surrogates and quantitative testing methodologies to ensure reliable and comparable assessment of CSTD performance in protecting healthcare workers.”

ASW Author Response: The authors thank the reviewer for this input and have incorporated the suggested reviewers form of words in the conclusions. Thank you once again for this helpful suggestion.

FIGURES AND TABLES

General Observations:

• All tables appear clear and relevant.

• Figures (if present) must be checked for resolution and labelling consistency.

ASW Author Response: The authors thank the reviewer for this input.

To Do:

• Confirm that all figures are labelled with axis titles, units, and legends.

ASW Author Response: The authors thank the reviewer for this input. This has been done.

• Ensure that statistical annotations (e.g., asterisks for p-values) are consistent and explained in the caption.

ASW Author Response: The authors thank the reviewer for this input. No statistical treatments were used and so No Action here.

LIMITATIONS SECTION

A dedicated limitations section is missing. Include one after the discussion to address:

• Potential influence of surrogate concentration on device behaviour.

• Absence of real-world validation.

• Limited scope of CSTD models tested.

• Possible variability in operator technique despite adherence to IFUs.

ASW Author Response: The authors thank the reviewer for this input. This has been added thank you and is presented below for clarity.

“LIMITATIONS SECTION

The study presented has a number of potential limitations which should be recognized and discussed:

1. The concentration of surrogate 2-POE could have an influence on the performance of the CSTD tested through lack of chemical compatibility. The concentration of 2.5% w/v was chosen so as to both optimize the signal response in the analytical method and reduce the potential for deleterious changes in the CSTD due to exposure to 2-POE. 2-POE 2.5% w/v is also close to the maximum solubility for 2-POE in water and so cannot be increased further. The authors performed functional testing on the four CSTD assessed in the study to satisfy themselves that the use of 2.5% w/v 2-POE as surrogate did not impact negatively on the performance of the CSTD.

2. The study presented was performed deliberately in a laboratory environment where background concentrations of 2-POE were controlled and mitigated. This was important because of the impact that high background levels of 2-POE would have on the experimental LOD and LLOQ. Real-world testing and validation could offer greater assurances that the results obtained are applicable to healthcare worker occupational environments. The testing conducted by Pengelly et al. [26] and Wheeler et al. [27] using 2-POE was performed at scale to better represent worker environments and conditions and the vapour concentrations for 2-POE obtained from these “real-world” tests were the same as the concentrations of 2-POE obtained from the present laboratory scale study. This does provide greater assurance, but more “real-world” testing is required and ideally testing using actual hazardous drugs although this might present an ethical challenge.

3. Only four types of CSTD were evaluated in the present study. This limits the scope of the present study work but when taken together with previously published works of Wilkinson et al. [21,22] there is an increasing body of containment data for CSTDs evaluated using 2.5% w/v 2-POE as surrogate in combination with the 2016 Draft NIOSH universal test protocol. Provided more CSTD can be evaluated using the same scientifically robust quantitative testing methodology this will assist in the selection of suitable CSTD technology within healthcare.

4. Variability in operator during NIOSH testing. It is feasible that different operators whilst adhering to the manufacturer’s instructions for use (IFU) could produce different outcomes from the NIOSH tests. In the present study this was accounted for by employing the same operators for all testing and ensuring that all steps were performed with the same timings between tests and all details were recorded contemporaneously using recording sheets.

5. Four replicates were performed for each of the two NIOSH tasks 1 and 2. This was done to comply with the current 2016 Draft NIOSH universal test protocol as published. However, it is accepted that a greater number of replicates may provide more robust data at an increase in cost. It would therefore be beneficial for a single site to perform a larger cohort of testing of different CSTDs with increased replication and for the results to be published for comparison with smaller scale testing to see if any differences are observed in outcomes.

6. Lack of correlation between releases of 2-POE as surrogate and actual hazardous drug exposure risk. This remains an important limitation which is accepted by the author. What is important is that CSTD should be able to demonstrate containment that is better than that of an “open” system of needle and syringe and this may not be the case. In the present study positive control releases of the surrogate 2-POE were performed at two volume sizes and each produced quantifiable signals for 2-POE with parts per billion volume concentrations of 2-POE in the chamber. Liquid releases of hazardous drug present the greatest exposure risk to healthcare workers based on the amount of hazardous drug that can be contained in liquid compared with vapour. More work needs to be performed however on correlation between healthworker exposure to liquid releases of hazardous drugs in a controlled quantifiable environment and health risks. The study presented does not attempt to make any correlation but rather focuses on the physical containment aspects of four chosen commercial CSTDs in terms of liquid and vapour containment and attempts to compare containment performance across the different CSTD technologies assessed.”

FINAL COMMENTS

While the manuscript presents scientifically relevant data and follows a rigorous testing protocol, it currently falls short of PLOS ONE’s standards for clarity, methodological transparency, and ethical disclosure. The following critical issues must be addressed before publication is warranted:

Major issues preventing recommendation for publication

1. Incomplete or Inconsistent Methods Section

• Lack of reproducibility details: Key aspects such as GC-MS parameters (e.g., column type, temperature ramping), surrogate preparation, and calibration procedures are either missing or underdeveloped.

ASW Responses: This detail has now been added.

• Insufficient statistical reporting: It is unclear which tests were used to compare device performance (e.g., ANOVA, t-test), whether multiple comparisons corrections were applied, or how outliers were handled.

ASW Responses: This has now been clarified – no statistical techniques were applied other than the analytical regression analysis from calibration curves and this has been made more overt and references to statistical differences removed from the text.

Recommendation: Expand the Methods section with full technical specifications and statistical methodology. Ensure that another lab could replicate your work using only this description.

ASW Responses: This detail has now been addressed.

2. Missing or Inadequate Limitations Section

• The study lacks a dedicated limitations section, which is essential for contextualising findings and acknowledging constraints.

No discussion is provided on: Whether the selected surrogate concentration (2.5% w/v 2-POE) affects CSTD integrity or behavior differently than actual hazardous drugs.

Whether operator variability was controlled or monitored.

The generalizability of findings beyond the four tested CSTDs.

ASW Responses: This detail has now been added.

Recommendation: Add a standalone Limitations section addressing these points and others related to internal and external validity.

3. Inconsistent Data Reporting and Figure/Table Integration

• Some figures and tables are referenced incorrectly or not at all in the main text.

• Units and formatting (e.g., ppbv, μL) are inconsistent across sections.

• There is no clear statement confirming whether raw data will be made publicly available, despite PLOS ONE’s strict open-data policy.

ASW Responses: This detail has now been added and concerns addressed including full disclosure of all data using Figshare as recommended.

Citation:

Wilkinson, Alan; Walker, Kate; Ozolina, Laima; Bon, Romana; Wallace, Andrew; Allwood, Michael Charles (2026). This is the data repository of Chromeleon CM7.3 chromatographic data for publication by Wilkinson et al. in Plosone journal.. figshare. Dataset. https://doi.org/10.6084/m9.figshare.33890500.v1

Recommendation: Conduct a comprehensive review of all figures and tables to ensure:

• Every one is explicitly cited in the text.

• Captions are self-explanatory.

• Units are consistent and SI-compliant.

• A Data Availability Statement is clearly included, indicating where data can be accessed.

ASW Responses: This detail has now been addressed.

4. Ethical and Funding Disclosures Require Clarification

• Although the authors state that “no competing interests exist,” they also disclose funding from ICU Medical, which provided materials and devices used in the study. This creates a potential conflict of interest that needs explicit acknowledgment.

ASW Responses: This detail has now been added

• There is ambiguity around the role of the funder: While the authors claim the funders had no influence, this should be stated more transparently.

ASW Responses: This detail has now been addressed.

Recommendation: Revise the Competing Interests and Funding Disclosure sections to fully align with PLOS ONE’s requirements. Consider including the following language:

“The funders had no role in study design, data collection and analysis, decision to publish, or preparation of the manuscript.”

ASW Responses: This detail has now been addressed.

5. Abstract and Conclusions Need Tightening

• The abstract lacks key information about sample size, statistical methods, and practical implications.

• The conclusion emphasizes protocol validation over real-world relevance.

ASW Responses: This detail has now been addressed.

Recommendation:

• Clarify what the differences between CSTD types mean for occupational safety.

• Emphasize how this study fills a gap in published evidence.

• Reframe conclusions to guide healthcare institutions in interpreting containment performance.

ASW Responses: This detail has now been addressed.

Strengths that support eventual acceptance

Despite these issues, the manuscript has several strengths that make it worth revising for eventual publication:

• Scientific merit: It addresses an important gap by comparing air-cleaning and barrier CSTDs using a semi-volatile surrogate under a standardised protocol.

• Rigorous methodology: Adherence to the 2016 Draft NIOSH protocol is commendable.

• Potential impact: Findings may inform purchasing decisions and regulatory guidance if interpreted carefully

Attachment Submitted filename: Response_to_Reviewers_auresp_3.docx

# Editor report: Decision Letter 3

**DOI:** 10.1371/journal.pone.0337729.r007

**Authors:** Antonio Peña-Fernández

24 Sep 2026

<p>Head-to-head containment performance testing of four commercially available closed-system transfer devices using the 2016 Draft NIOSH test protocol and 2.5% w/v 2-phenoxyethanol as challenge agent.

PONE-D-25-12716R3

Dear Dr. Wilkinson,

We’re pleased to inform you that your manuscript has been judged scientifically suitable for publication and will be formally accepted for publication once it meets all outstanding technical requirements.

Within one week, you’ll receive an e-mail detailing the required amendments. When these have been addressed, you’ll receive a formal acceptance letter and your manuscript will be scheduled for publication.

An invoice will be generated when your article is formally accepted. Please note, if your institution has a publishing partnership with PLOS and your article meets the relevant criteria, all or part of your publication costs will be covered. Please make sure your user information is up-to-date by logging into Editorial Manager at Editorial Manager® and clicking the ‘Update My Information' link at the top of the page. For questions related to billing, please contact billing support.

If your institution or institutions have a press office, please notify them about your upcoming paper to help maximize its impact. If they’ll be preparing press materials, please inform our press team as soon as possible -- no later than 48 hours after receiving the formal acceptance. Your manuscript will remain under strict press embargo until 2 pm Eastern Time on the date of publication. For more information, please contact onepress@plos.org.

Kind regards,

Antonio Peña-Fernández, PhD

Academic Editor

PLOS One

Additional Editor Comments (optional):

Dear Authors,

Thank you for submitting the revised version of your manuscript and for addressing the reviewers’ comments.

The manuscript has been satisfactorily revised, and no further changes are required from my side.

Best wishes,

Antonio

Reviewers' comments:

# Editor report: Acceptance letter

**DOI:** 10.1371/journal.pone.0337729.r008

**Authors:** Antonio Peña-Fernández

PONE-D-25-12716R3

PLOS One

Dear Dr. Wilkinson,

I'm pleased to inform you that your manuscript has been deemed suitable for publication in PLOS One. Congratulations! Your manuscript is now being handed over to our production team.

At this stage, our production department will prepare your paper for publication. This includes ensuring the following:

* All references, tables, and figures are properly cited

* All relevant supporting information is included in the manuscript submission,

* There are no issues that prevent the paper from being properly typeset

You will receive further instructions from the production team, including instructions on how to review your proof when it is ready. Please keep in mind that we are working through a large volume of accepted articles, so please give us a few days to review your paper and let you know the next and final steps.

Lastly, if your institution or institutions have a press office, please let them know about your upcoming paper now to help maximize its impact. If they'll be preparing press materials, please inform our press team within the next 48 hours. Your manuscript will remain under strict press embargo until 2 pm Eastern Time on the date of publication. For more information, please contact onepress@plos.org.

You will receive an invoice from PLOS for your publication fee after your manuscript has reached the completed accept phase. If you receive an email requesting payment before acceptance or for any other service, this may be a phishing scheme. Learn how to identify phishing emails and protect your accounts at https://explore.plos.org/phishing.

If we can help with anything else, please email us at customercare@plos.org.

Thank you for submitting your work to PLOS One and supporting open access.

Kind regards,

PLOS One Editorial Office Staff

on behalf of

Dr. Antonio Peña-Fernández

Academic Editor

PLOS One
